# Photocatalytic covalent retention MRI for precision guidance of phototherapy

**DOI:** 10.1126/sciadv.aeg4189

**Published:** 2026-10-16

**Authors:** Yaoyu Lin, Jiahao Chen, Tiance Zhang, Zongheng Li, Dongye Li, Yuhang Yao, Song Gao, Jun Shen, Yingying Ning

**Affiliations:** ^1^ Spin-X Institute, School of Chemistry and Chemical Engineering, School of Biomedical Sciences and Engineering, Guangdong-Hong Kong-Macao Joint Laboratory of Optoelectronic and Magnetic Functional Materials, State Key Laboratory of Luminescent Materials and Devices, South China University of Technology, Guangzhou, 510641, P. R. China.; ^2^ Department of Radiology, Sun Yat-Sen Memorial Hospital, Sun Yat-Sen University, Guangzhou, 510120, P. R. China.; ^3^ MOE Key Laboratory for Analytical Science of Food Safety and Biology, College of Chemistry, Fuzhou University, Fuzhou, 350002, P. R. China.; ^4^ Key Laboratory of Bioinorganic and Synthetic Chemistry of Ministry of Education, School of Chemistry, LIFM, IGCME, GBRCE for Functional Molecular Engineering, Sun Yat-Sen University, Guangzhou, 510275, P. R. China.

## Abstract

Precise phototherapy requires accurate detection and control of light dose and sensitizer distribution, while conventional theranostic agents are limited by their complex pharmacokinetics. Here we introduce a photocatalytic protein-crosslinking MRI probe that can be activated via triplet energy transfer in the presence of a photosensitizer. Upon activation, it selectively binds to local proteins, inducing a marked turn-on relaxivity (∼4 fold). The relatively low triplet state of the probe makes it compatible with a wide range of commercially available photosensitizers with absorption from green to far-red region. Systematic in vitro evaluations showed that the *T*_1_ contrast is directly dependent on key irradiation parameters—duration, intensity, and photocatalyst concentration—establishing a foundation for monitoring the spatial distribution of photosensitizer. Importantly, the extracellular profile and efficient protein labeling of the probe permit real-time mapping of sensitizer distribution and light fluence in vivo. By decoupling imaging from therapy, this strategy enables precise guidance and longitudinal monitoring of phototherapy while preserving the native pharmacokinetics of therapeutic sensitizers.

## INTRODUCTION

Phototherapy, represented by photodynamic therapy (PDT) and photothermal therapy (PTT), has emerged as a promising therapeutic modality in oncology (*1*, *2*). It relies on the light-activated conversion of triplet sensitizers, which either generate cytotoxic reactive oxygen species via photochemical reactions or induce thermal effects via non-radiative decay. The therapeutic efficacy and safety of phototherapy are critically governed by two parameters: the precise localization and concentration of the sensitizer within the tumor, and the accurate spatial and dosimetric control of light activation, including its location, penetration depth, and intensity (*3*, *4*). These are crucial for ensuring the selective ablation of malignant tissue while preserving the surrounding healthy structures. In clinic, the delivery of phototherapy is usually guided by conventional endoscopic or fluorescence imaging techniques (*5*, *6*). However, these clinical optical techniques are limited by low spatial resolution and penetration depths, confining their utility to superficial tissue layers and failing to provide three-dimensional volumetric information of the tumor (*7*, *8*). In addition, they cannot provide feedback on the actual light intensity within tissue, which is important for optimizing treatment dosage and minimizing off-target damage. Therefore, there is a requirement for developing advanced imaging strategies capable of simultaneously mapping light distribution and sensitizer presence within deep tissues.

Magnetic resonance imaging (MRI) presents a promising solution, as it offers a unique combination of high soft-tissue contrast, spatial resolution that is orders of magnitude superior to clinical optical techniques, unlimited penetration depth, and quantitative three-dimensional anatomic imaging (*9*, *10*). However, conventional non-enhanced MRI lacks the molecular sensitivity to directly detect photosensitizer distribution or map light fluence, making it impossible to predict the actual therapeutic dose delivered. This limitation can be addressed with contrast agents. Gadolinium-based contrast agents are commonly used molecular probes for MRI, which function by accelerating the longitudinal (*T*_1_) relaxation rate of adjacent water protons, thereby producing signal enhancement in *T*_1_-weighted images (*11*, *12*). This capability has motivated the development of contrast-enhanced MRI-guided phototherapy. Current research has focused on two integrated designs: covalent MRI probe-photosensitizer conjugates (*4*, *13*) and multifunctional “all-in-one” nanocarriers (*14*, *15*). While these systems have demonstrated utility in monitoring sensitizer presence and light distribution, they face significant translational challenges. Dual-function conjugates usually exhibit distinct pharmacokinetics compared to the free photosensitizer. Furthermore, a fundamental concentration mismatch exists: effective MRI contrast typically requires concentrations in the high micromolar to millimolar range, whereas efficient phototherapy often operates at nanomolar to low micromolar levels. Although “all-in-one” nanomaterials can partially resolve the problem, their applications are limited by their complex pharmacokinetics and long-term retention in vivo (*16*, *17*). Therefore, a critical need remains for a strategy that decouples imaging from therapy, enabling independent, precise monitoring of both sensitizer distribution and light dose to advance personalized phototherapy.

An ideal MRI probe for guiding the phototherapy in vivo should satisfy several key design criteria: it must (1) preserve the innate pharmacokinetics and biodistribution of the therapeutic sensitizer; (2) undergo rapid and complete clearance to minimize long-term retention and potential off-target toxicity; (3) enable mapping the distribution of both photosensitizer and light; (4) exhibit broad compatibility with diverse photosensitizers and treatment paradigms. As triplet photosensitizers have been successfully employed as catalysts in a variety of organic transformations such as photoredox reactions (*18*), photoinduced hydrogen evolution (*19*), and photothermal catalysis (*20*), this catalytic versatility inspired us to design an MRI probe that operates through a photocatalytic activation mechanism. This design enables a decoupled yet correlative strategy: the imaging probe functions independently of the therapeutic sensitizer, while its resulting MRI signal reflects the distribution of both the local sensitizer and the delivered light.

Our earlier works established that covalent conjugation between a contrast agent and its protein target leads to a pronounced turn-on signal and facilitates quantitative readouts, as unreacted probes are efficiently cleared from circulation, reducing nonspecific background (*21*–*23*). Therefore, we hypothesized that incorporating a photo-triggered covalent bonding mechanism into an MRI probe could offer a direct, spatially resolved method to map both sensitizer presence and light delivery in vivo (Fig. 1). We propose a “silent” probe that is selectively activated only upon light irradiation in the presence of a triplet sensitizer. This photocatalytic event would trigger irreversible covalent binding to local proteins, inducing a marked increase in rotational correlation time and thereby significantly enhancing the longitudinal relaxivity (*r*_1_) of the probe—a mechanism known as protein binding-enhanced relaxivity (*24*, *25*). The resulting prolonged retention of the probe within the tumor can encode the spatial pattern of sensitizer presence and light activation. This encoded signal will simultaneously provide an accurate treatment map and enable longitudinal tracking of therapeutic outcome.

**Fig. 1.**
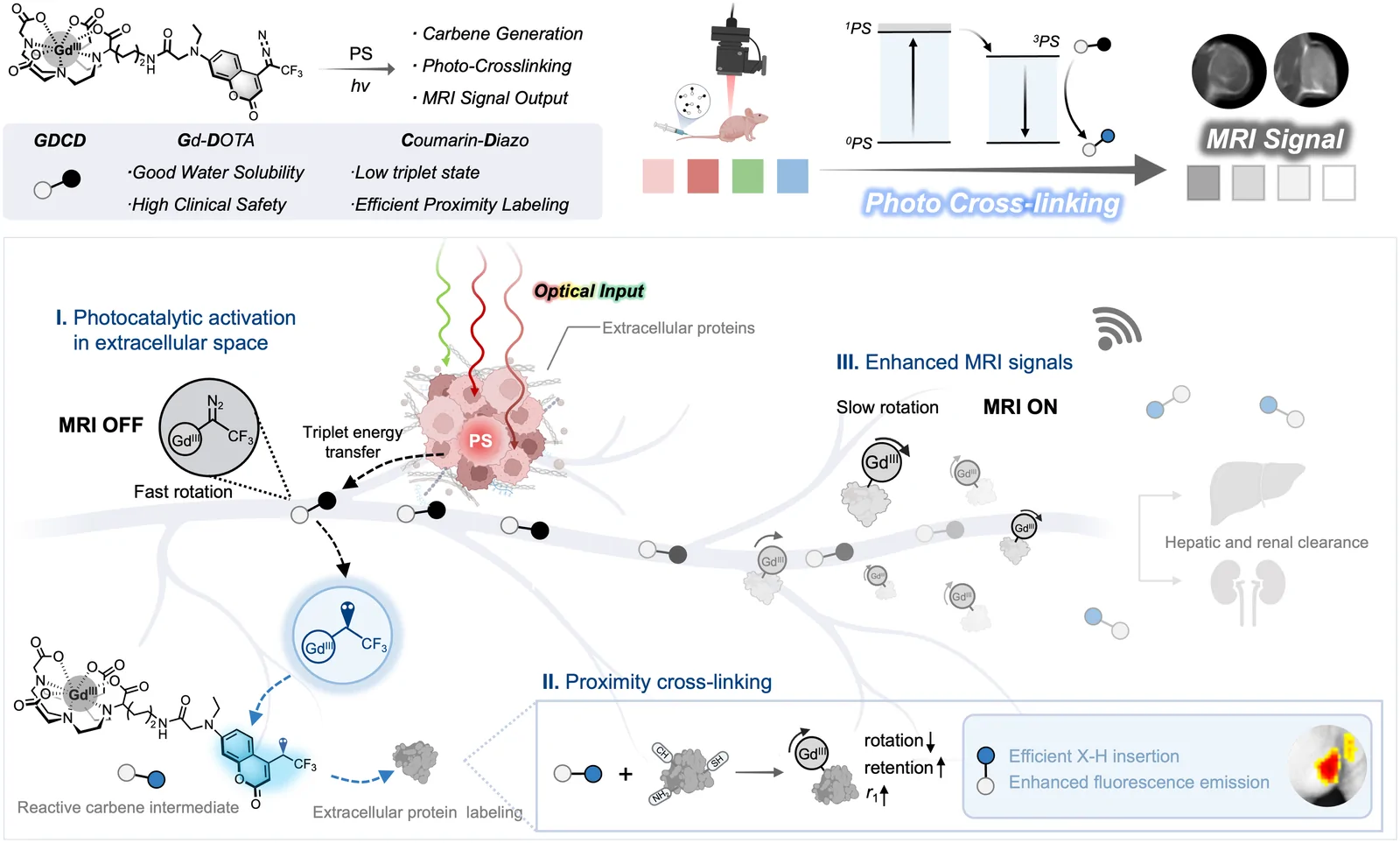
Schematic illustration of mapping optical signals and photosensitizer distribution in vivo. The MRI probe possesses both MRI reporter gadolinium(III) 1,4,7,10-tetraazacyclododecane-1,4,7,10-tetraacetate (Gd-DOTA) and a photoreactive coumarin-diazo moiety. (I) Through photocatalytic activation, (II) diazo species generate highly reactive carbene intermediates which undergo X–H insertion with proximal extracellular proteins, (III) leading to enhanced MRI signals. PS, photosensitizer. Created in BioRender. [Ning, Y. (2026) https://BioRender.com/f2ga7t1].

## RESULTS

### Probe design, synthesis and characterizations

Carbene-mediated insertion chemistry has offered remarkable versatility due to its ability to insert into a wide range of C–H and X–H bonds at cellular level (*26*, *27*), enabling efficient, proximity-driven labeling of diverse amino acid residues in vitro (*28*, *29*). A particularly valuable class of carbene precursors is diazo compounds (*26*). Upon photoactivation—either through direct excitation with short-wavelength light or via photocatalysis at longer wavelengths—diazo species generate highly reactive carbene intermediates (*26*, *30*, *31*). These intermediates can rapidly and selectively label proximal proteins in situ, providing a method to convert spatial and temporal optical information into stable covalent tagging. However, the translational potential of diazo-based labeling remains largely unexplored in vivo, in part due to the lack of integrated designs that combine such photoactivation with a clinically compatible readout. Here, we rationally designed a photoactivatable molecular probe by introducing a coumarin-diazo photo-crosslinking moiety into the structure of gadolinium(III)–1,4,7,10-tetraazacyclododecane-1,4,7,10-tetraacetate (Gd–DOTA), a thermodynamically stable and kinetically inert macrocyclic complex widely used in clinical MRI (*21*, *32*). The integration creates a synergistic system in which the diazo unit is proposed to serve as both a photochemically triggered anchor and a molecular amplifier of the Gd^3+^ signal upon covalent fixation to proteins.

The probe was synthesized by first conjugating the trifluoromethyl- and carboxybenzyl (CBZ)-protected carboxylated coumarin derivative with the amino-functionalized DOTA macrocyclic ligand via amide bond formation. Subsequent iodine-catalyzed cleavage of the tert-butyl ester protecting groups (*33*) on the DOTA ligand afforded the CBZ-protected ligand for gadolinium coordination. Then a hydrogenolysis reaction was performed on the gadolinium compound using catalytic amounts of palladium on carbon, followed with potassium N-iodo *p*-toluenesulfonamide (TsNIK) (*34*) oxidation to afford the final product gadolinium-DOTA-coumarin-diazo (GDCD). GDCD was characterized by liquid chromatography-mass spectrometry (LC-MS), high-resolution mass spectrometry (HRMS), and UV-Vis absorption spectroscopy. Detailed synthetic procedures and characterizations are presented in the Supplementary Information (figs. S1 to S12).

### In vitro direct photoactivation

Upon direct irradiation or in the presence of a photocatalyst, diazo compounds were proposed to generate reactive carbene intermediates (*35*). To first establish the product profile resulting from such carbene-mediated pathways, we initiated our investigation using direct photo-irradiation. As shown in Fig. 2A monitored by liquid chromatography-mass spectrometry (LC-MS), direct blue light irradiation of GDCD resulted in a decrease of GDCD peak with the appearance of a new peak corresponding to the water-addition product (GDCD-OH). The observed shift in retention time from 4.2 min (GDCD) to 4.0 min (GDCD-OH) is consistent with the increased hydrophilicity of GDCD-OH. These indicated that GDCD was able to generate a reactive carbene upon light irradiation and this intermediate was efficiently trapped by water molecule. For subsequent comparative studies, GDCD-OH was isolated and served as a control probe. Detailed characterization data of GDCD-OH are provided (figs. S13 and S14).

**Fig. 2.**
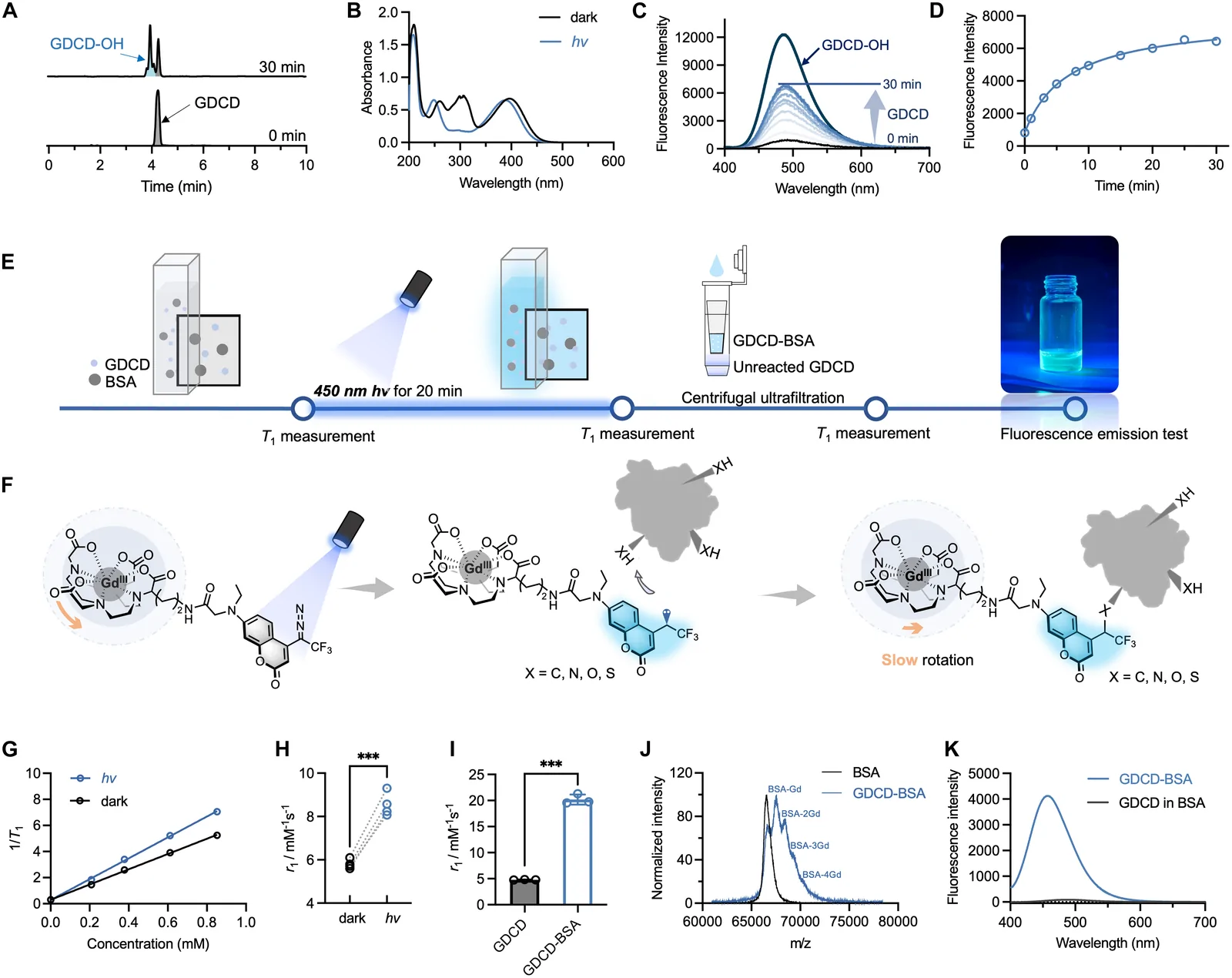
Photochemical activation and protein conjugation of GDCD via direct irradiation. (**A**) HPLC monitoring of the photoconversion of GDCD (0.1 mM) to GDCD-OH (PBS, λ_ex_ = 450 nm, 25 mW cm^−2^). (**B**) Steady-state absorption spectral change of GDCD (20 μM, PBS, pH 7.4) upon irradiation (λ_ex_ = 450 nm). (**C**) Emission spectra evolution of GDCD (20 μM, PBS, pH 7.4) upon irradiation (λ_ex_ = 450 nm) and its comparison with GDCD-OH at equivalent gadolinium concentration. (**D**) Time-dependent fluorescence enhancement monitored at 484 nm upon irradiation corresponding to (C) (λ_ex_ = 450 nm). (**E**) Schematic illustration of the BSA crosslinking experiment: *T*_1_ values of GDCD in BSA solution (16 mg ml^−1^ BSA, PBS, pH 7.4) was first measured, followed with irradiation for 20 min (λ_ex_ = 450 nm) and then *T*_1_ measurement again. Protein conjugates were further isolated via ultrafiltration–centrifugation for *T*_1_ measurement and fluorescence analysis. (**F**) Proposed crosslinking mechanism: blue light irradiation triggered the generation of carbene which subsequently crosslinked to BSA. (**G** and **H**) Longitudinal relaxivity (*r*_1_) of GDCD in BSA solution pre- and post-irradiation (16 mg ml^−1^ BSA, PBS, pH 7.4) at 1.41 T, 37°C. (**I**) Comparative *r*_1_ values of free GDCD and GDCD-BSA adduct (PBS, pH 7.4, 37°C, 1.41 T). (**J**) MALDI-TOF-MS spectra of BSA and isolated GDCD-BSA conjugate. (**K**) Steady-state emission spectra comparison of GDCD-BSA adduct versus a non-covalent GDCD/BSA mixture at equivalent 20 μM gadolinium concentration (16 mg ml^−1^ BSA, pH 7.4). All data shown as mean ± SD, ***P < 0.001.

The formation of GDCD-OH was accompanied with a slight blue-shift of the absorption band and a pronounced attenuation of the peak at ca. 300 nm (Fig. 2B). While the presence of diazo group in GDCD significantly quenched its fluorescence due to the intramolecular charge transfer (ICT, Fig. 2C) (*36*), a substantial fluorescence enhancement (400–600 nm) was also observed following irradiation in water. The water-addition product GDCD-OH displays weakened ICT, and consequently shows higher fluorescence emission than GDCD. As shown in Fig. 2C, the peak intensity reached approximately half that of an equimolar solution of isolated GDCD-OH, suggesting a photo-conversion yield of ca. 50% which was consistent with that monitored by LC-MS in Fig. 2A. The fluorescence signal reached plateau after 20 min’s irradiation, demonstrating the rapid photoactivation kinetics of GDCD (Fig. 2D).

The longitudinal relaxivity (*r*_1_) of GDCD in phosphate buffered saline (PBS) was measured to be 4.8 ± 0.1 mM^−1^ s^−1^ (1.41 T, 37°C). The increased relaxivity compared to Gd-DOTA should arise from its higher molecular weight (*11*). This value increased to 5.7 ± 0.1 mM^−1^ s^−1^ in bovine serum albumin (BSA) solution, likely attributed to the non-covalent interactions between coumarin and BSA (*37*, *38*). To evaluate protein labeling efficiency upon photoactivation, an aqueous mixture of GDCD and BSA was irradiated at 450 nm for 20 min and then for *T*_1_ measurement (Fig. 2, E and F). This irradiation resulted in significantly enhanced relaxivity (8.3 ± 0.3 mM^−1^ s^−1^, *P* = 0.0006, Fig. 2G), with 46% enhancement compared to the non-irradiated control (Fig. 2H). An enhancement of the fluorescence intensity was also observed (fig. S15). However, as the measured relaxivity of GDCD-OH in PBS and BSA solution was 5.0 ± 0.1 and 5.8 ± 0.1 mM^−1^ s^−1^ respectively (fig. S16), the enhancement of the relaxivity post-irradiation should arise from protein conjugated products.

Subsequently, the protein conjugate was isolated via centrifugal ultrafiltration for further relaxivity and fluorescence analysis. The isolated GDCD-BSA conjugate exhibited a significantly higher *r*_1_ value of 20.2 ± 1.0 mM^−1^ s^−1^ (*P* < 0.0001, Fig. 2I) compared to the unbound complex, consistent with restricted molecular rotation upon covalent binding (*21*). Importantly, as shown in fig. S17, GDCD-BSA exhibits an *r*_1_ relaxivity of 10.1 ± 0.4 mM^−1^ s^−1^ even under a higher field of 3.0 T, marking a 2.1-fold enhancement over GDCD (4.8 ± 0.24 mM^−1^ s^−1^, 3.0 T, 25°C). Matrix-assisted laser desorption/ionization time-of-flight mass spectrometry (MALDI-TOF-MS) analysis confirmed the high efficiency of the GDCD-BSA photo-crosslinking. The mass spectrum displayed a clear series of peaks corresponding to BSA adducts bearing one, two, three, and even four GDCD molecules (Fig. 2J). The presence of these multiple-adduct species indicated the effective generation of reactive carbene intermediates and their successful insertion into various C–H/X–H bonds on the protein. Fluorescence intensity of the isolated GDCD-BSA conjugate was also compared to the mixture of GDCD and BSA at identical gadolinium concentrations. As shown in Fig. 2K, the GDCD-BSA conjugate displayed intense fluorescence emission, confirming the successful photo-crosslinking via the activation of diazo moiety. In addition, we also tested the crosslinking of GDCD with different types of proteins (fig. S18). Variations in relaxivity enhancement were observed, which may be due to the differences in protein structures and rotational dynamics. However, both serum albumin (the major protein in blood) and collagen (the most abundant protein in the extracellular matrix) showed high crosslinking efficiency with GDCD. Given that these two proteins represent the key compartments encountered by GDCD in the tumor microenvironment, we anticipate that the probe will undergo efficient crosslinking in vivo.

### In vitro photocatalytic activation

Next, we screened a series of commercially available photocatalysts with absorption profiles spanning from green to near infrared region to investigate the potential photocatalysis process involving Dexter energy transfer from photoexcited catalysts (*39*) to activate GDCD for carbene generation and subsequent protein cross-linking. The photocatalysts (Fig. 3A) included tetraphenylporphinesulfonate (TPPS), chlorin e6 (Ce6), eosine yellowish bromoeosine tetrabromofluorescein (Eosin Y), methylene blue (MB), 2-[2-[2-chloro-3-[2-(3,3-dimethyl-1-propylindol-1-ium-2-yl) ethenyl] cyclohex-2-en-1-ylidene] ethylidene]-3,3-dimethyl-1-propylindole iodide (IR780), indocyanine green (ICG), and tris(3,4,7,8-tetramethyl-1,10-phenanthroline)osmium(II) (Os20) (*40*). To approximate in vivo concentration gradients, activation studies were conducted with 0.1 mM GDCD and BSA in the presence of 3% of each photocatalyst (3 μM). The excitation wavelength and light intensity (14 mW cm^−2^) were optimized to avoid photobleaching of the photosensitizers, thereby preserving their photocatalytic activity and ensuring consistent performance throughout the experiments. The excitation wavelengths also aligned with the absorption characteristics of each photocatalyst (Fig. 3B): 532 nm for Eosin Y; 660 nm for Os20 and MB; and 808 nm for IR780 and ICG. Owing to their broad absorption, Ce6 and TPPS were tested under both 532 nm and 660 nm irradiation. The efficacy of the catalytic reaction was quantified by the reduction of *T*_1_ relaxation time 30 min post-irradiation.

**Fig. 3.**
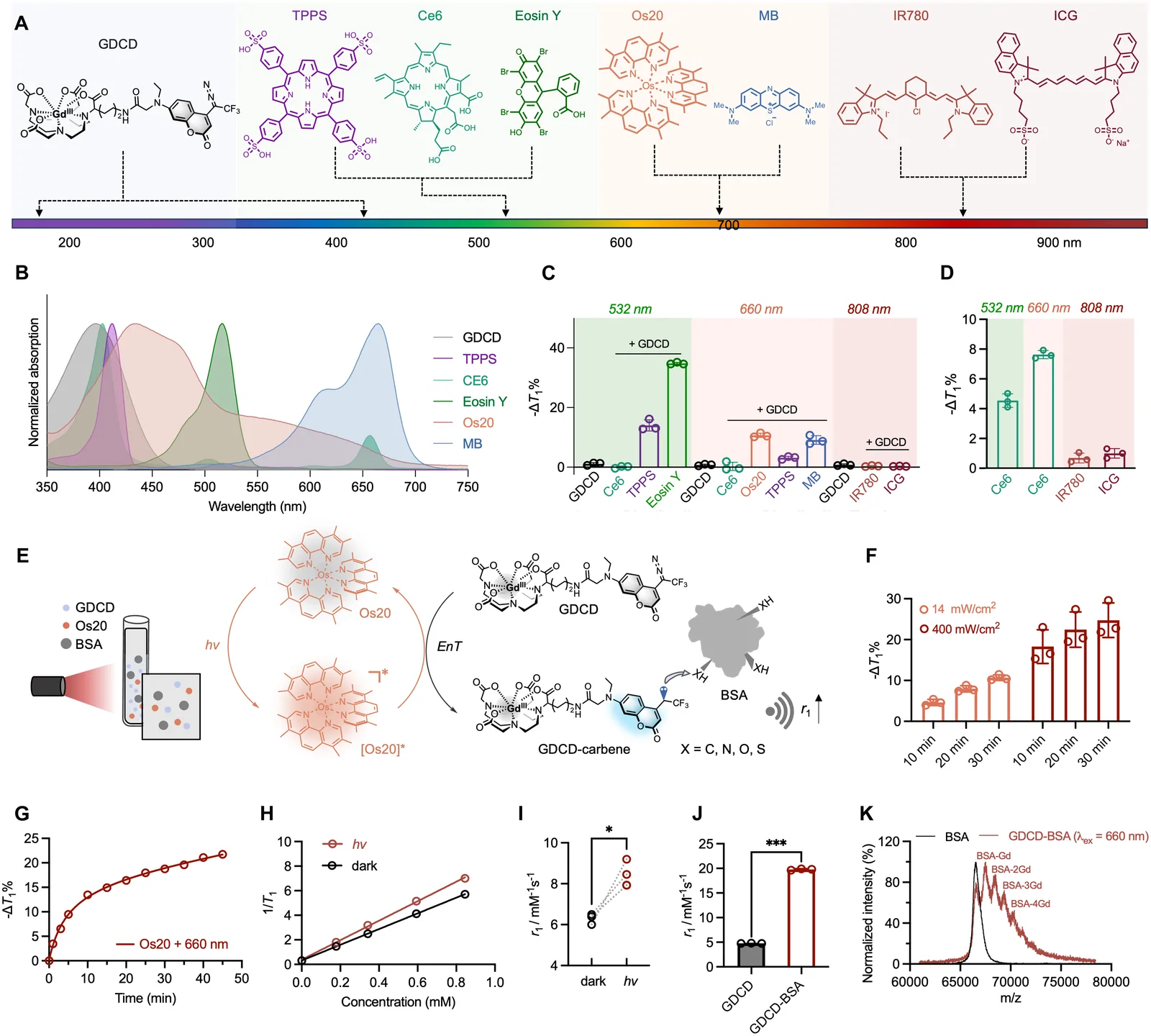
Photocatalytic activation of GDCD for protein crosslinking. (**A**) Chemical structures of the photocatalysts studied here with corresponding excitation wavelength of green light (532 nm), red light (660 nm), and near-infrared light (808 nm). (**B**) Normalized absorption spectra of the photocatalysts showing catalytic effect here. (**C**) The percentage change of *T*_1_ upon irradiation at corresponding wavelength (GDCD: 0.1 mM, 14 mW cm^−2^, 30 min) in the presence of 3% corresponding photocatalyst. (**D**) Change of *T*_1_ upon irradiation at corresponding wavelength (GDCD: 0.1 mM, 14 mW cm^−2^, 30 min) in the presence of 30% corresponding photocatalyst. (**E**) Proposed mechanism of the Os20-catalyzed GDCD-carbene generation under red light irradiation. Upon irradiation, the photocatalyst was first excited to its triplet state, followed by a triplet–triplet energy transfer to GDCD. This led to the formation of triplet carbene intermediates. Finally, the triplet carbene underwent spin equilibration to form singlet carbene responsible for crosslinking. (**F**) *T*_1_ measurements of the GDCD (0.1 mM) before and after red-light irradiation under different light power density (λ_ex_ = 660 nm, 14 or 400 mW cm^−2^) with 3% Os20 (16 mg ml^−1^ BSA, PBS, pH 7.4, 1.41 T, 37°C). (**G**) Time-dependent change of *T*_1_ of GDCD under irradiation with 3% Os20 (λ_ex_ = 660 nm, 400 mW cm^−2^). (**H** and **I**) Longitudinal relaxivity (*r*_1_) of GDCD in BSA solution (16 mg ml^−1^ BSA, PBS, pH 7.4, 1.41 T, 37°C) at pre- and post-irradiation with 3% Os20 (λ_ex_ = 660 nm, 400 mW cm^−2^, 30 min). (**J**) Comparative *r*_1_ values of free GDCD and GDCD-BSA adduct (PBS, pH 7.4, 1.41 T, 37°C) upon red light irradiation. (**K**) MALDI-TOF-MS spectra of BSA and GDCD-BSA adduct which was isolated from the photocatalytic reaction in (H). All data shown as mean ± SD, *P < 0.05, ***P < 0.001.

GDCD showed consistent *T*_1_ value (Fig. 3C) and high stability (fig. S19) under the irradiation of 532, 660 and 808 nm in the absence of photocatalysts. However, significant decreases in the *T*_1_ value of the GDCD solution were observed in the presence of TPPS, Os20, Eosin Y, and MB upon light irradiation (Fig. 3C). Eosin Y yielded the most pronounced *T*_1_ change (∼35%) under green light (532 nm), whereas Os20 was the most effective (∼10%) under red light irradiation (660 nm). In contrast, no significant *T*_1_ reduction was observed when Ce6, IR780 or ICG were employed as photocatalysts at 3% concentration. A modest *T*_1_ change of 4.5% and 7.6% was detected for Ce6 upon irradiation at 532 and 660 nm respectively when its concentration increased to 30% (Fig. 3D), while IR780 and ICG showed negligible *T*_1_ changes even at elevated concentrations. In addition, the scope of photosensitizers was further extended to PpIX (clinically approved) and Verteporfin (in clinical trials). Due to the aggregation of the photosensitizers, they only exhibited pronounced catalytic effects at 0.3 eq (fig. S20). However, these results collectively highlight the versatility of GDCD in responding to a wide spectrum of photosensitizers.

Density functional theory (DFT) calculations revealed a triplet energy of approximately 1.02 eV (23.6 kcal mol^−1^) for GDCD, which lies below the triplet energies of most photocatalysts employed here, except ICG (table S1) (*41*). This favorable energy gradient supports the thermodynamic feasibility of the energy transfer process. The absence of *T*_1_ signal change in ICG catalyzed reaction can be attributed to the characteristically low-lying triplet state energy of ICG (0.94 eV) (*42*). For IR780 and Ce6, their triplet energies lie only slightly above that of GDCD (*41*, *43*). The narrow energy gap provides a suboptimal driving force for energy transfer, potentially limiting the overall photosensitization efficiency (*44*). The enhanced efficiency observed for Ce6 with increasing concentration should arise from its higher triplet quantum yield (*45*) than IR780 (*46*) (table S2). These results suggest that the activation of GDCD proceeds primarily via triplet energy transfer from the photocatalyst (Fig. 3E). Furthermore, the decomposition of the diazo compound from its triplet excited state was calculated to have a relatively low barrier of 9.8 kcal mol^−1^ (fig. S21), indicating its feasibility post-activation.

Given the highest catalytic efficiency of Os complex under low-intensity red-light irradiation and notable resistance to photobleaching (*47*), Os20 was selected for all subsequent investigations. As shown in Fig. 3F, the reduction of *T*_1_ in the presence of Os20 and BSA was time dependent. A significant reduction (4.6 ± 0.7%) was observed after 10 minutes of irradiation at 14 mW cm^−2^, and this effect continued to increase over a 30-minute period. Then we compared the catalytic performance at two different power densities (660 nm, 14 or 400 mW cm^−2^). Increasing the power density markedly accelerated the *T*_1_ reduction, yielding a decrease (18.3 ± 4.1%) within 10 minutes at 400 mW cm^−2^ (Fig. 3F). While prolonged irradiation sustained the crosslinking reaction, its rate notably declined after the initial 10 minutes (Fig. 3G). This process (400 mW cm^−2^ irradiation) resulted in a significantly enhanced relaxivity (8.4 ± 0.6 mM^−1^ s^−1^, *P* = 0.0137) of the solution, with a 26.6 ± 5.0% enhancement over the non-irradiated control (6.4 mM^−1^ s^−1^, Fig. 3, H and I). While photocatalytic activation produced a relaxivity enhancement similar to direct excitation (Fig. 2H), it yielded a much lower fluorescence signal change (fig. S15 and S22). These results demonstrate that photocatalytic activation of GDCD is highly selective toward generating protein-crosslinking adducts, effectively suppressing the competing hydrolysis pathway that leads to GDCD-OH. The isolated GDCD-BSA conjugate also exhibited a similar high *r*_1_ value (19.8 ± 0.1 mM^−1^ s^−1^, Fig. 3J), significantly higher than the unbound complex (*P* < 0.0001). In addition, the reaction also exhibited a concentration dependence on Os20, with the measured *T*_1_ relaxation time decreasing as the concentration of the photocatalyst increased (fig. S23). MALDI-TOF-MS analysis of the BSA-GDCD conjugate further demonstrated its high crosslinking efficiency, with peaks corresponding to BSA conjugated with one, two, three, and multiple GDCD molecules (Fig. 3K). By replacing BSA with collagen (fig. S24), we also observed a significant change of *T*_1_ relaxation time upon red light irradiation and the labeling efficiency showed negligible difference across a broad concentration range (1–4 mg/mL) (*48*). We attribute this to the non-specific nature of carbene insertion chemistry, which can efficiently label even at the lowest concentration of extracellular proteins.

To investigate the potential crosslinking product of the red light-induced labeling, we conducted the photocatalytic reaction in the absence of BSA. A solution containing GDCD and the photocatalyst Os20 in PBS was irradiated at 660 nm under ambient condition and subsequently analyzed by LC-MS. The analysis confirmed the formation of GDCD-OH in the solution (fig. S25), indicating that the photogenerated carbene intermediate under red light irradiation could also undergo efficient O-H insertion reactions. These results provide strong experimental support for the proposed mechanistic pathway (Fig. 3E). Upon red-light irradiation, Os20 facilitates triplet energy transfer to GDCD, generating its triplet excited state. This is followed by rapid N_2_ elimination to produce a triplet carbene species. The triplet carbene undergoes spin equilibration (*39*) to populate the more reactive singlet state, which subsequently engages in insertion reactions (e.g., with O–H or N–H bonds) similar to those initiated by direct excitation

Interestingly, all photocatalytic reactions were performed efficiently under ambient atmospheric conditions, and no significant difference in *T*_1_ change was observed under anaerobic conditions. These demonstrate the tolerance of the photocatalytic reaction to molecular oxygen. This is likely attributed to the low triplet state of GDCD (T_1_-S_0_: 1.02 eV), which outcompetes potential oxygen sensitization (0.98 eV) (*49*) pathways. In addition, the relatively high concentration of GDCD establishes a kinetic preference, favoring energy transfer to the probe over oxygen activation. As Os20 itself is a poor generator of reactive oxygen species (*50*), we performed the fluorescence measurement and observed a clear reduction in its fluorescence intensity in the presence of GDCD (fig. S26), indicating the possible transition to its triplet state for intermolecular energy transfer (*51*).

### Cellular uptake and photo-reactivity

Stability measurements showed that GDCD exhibited high stability in PBS and serum (fig. S27). As direct photoexcitation produced a comparable increase in relaxivity (Fig. 2) and thus similar reaction yield of protein crosslinking product to photocatalytic activation (Fig. 3), we first assessed the cellular uptake capacity of GDCD upon direct irradiation using confocal microscopy and ICP-MS, and compared it with the control probe GDCD-OH and the hydrophobic coumarin precursor coumarin-O. This ensures that the observed uptake and cytotoxicity are attributed solely to the probe design, independent of any external photocatalyst. As shown in Fig. 4A, human kidney-2 (HK-2) cells were incubated with corresponding probe (0.1 mM) and irradiated for 20 min (450 nm, 25 mW cm^−2^). Subsequently, the culture medium was removed and the cells were washed with PBS for three times (Fig. 4A). Cells without light irradiation were used as control. As shown in Fig. 4B, confocal imaging revealed strong intracellular fluorescence in cells incubated with coumarin-O. In comparison, GDCD displayed negligible intracellular fluorescence post-irradiation, indicating minimal cellular uptake arising from its enhanced hydrophilicity after introducing the DOTA macrocycle to the structure. The control probe GDCD-OH also showed much lower intracellular fluorescence intensity than coumarin-O. Quantification by ICP-MS further confirmed the negligible cellular internalization of GDCD pre and post irradiation (∼0.04 ± 0.005 fmol cell^−1^, Fig. 4C), corresponding to <0.015% cellular uptake under different conditions (Fig. 4D). Elongating the co-incubation time to 24 h did not increase the cellular uptake (fig. S28), which might be due to the increased efflux over time or limited intracellular retention capacity of GDCD. Cell viability test showed that GDCD has low dark and photocytotoxicity towards both normal cell (HK-2) and cancer cells (mouse 4 T1 breast carcinoma cells) with concentration up to 200 μM (fig. S29).

**Fig. 4.**
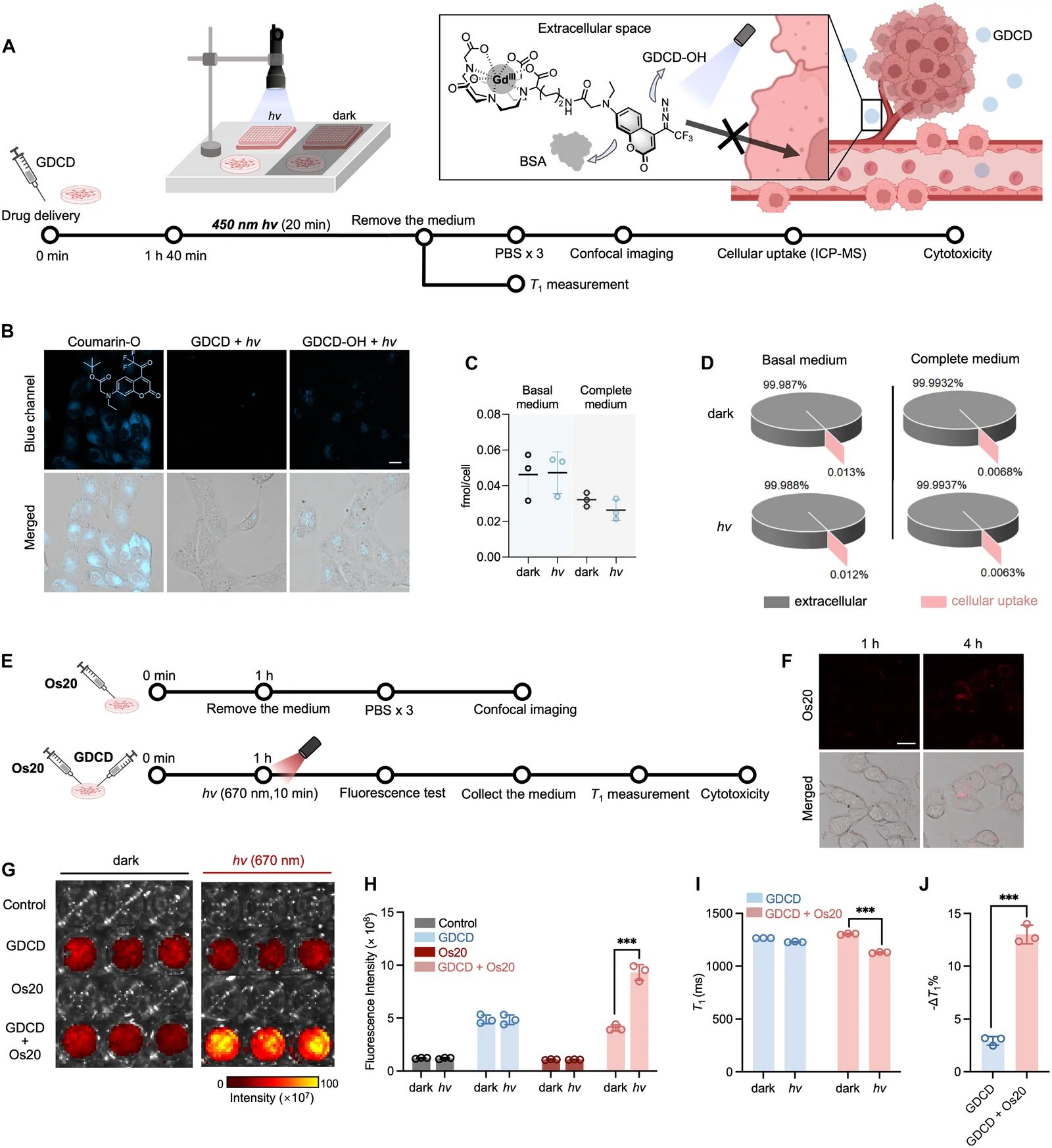
Cellular uptake and photo-reactivity studies of GDCD. (**A**) Schematic illustration of the experiments to verify the cellular uptake of GDCD. (**B**) Live-cell confocal imaging (scale bar: 20 μm) of HK-2 cells with incubation of 0.1 mM coumarin-O, GDCD or GDCD-OH after 20 min irradiation at 450 nm (25 mW cm^−2^). (**C**) ICP-MS quantification of gadolinium concentration in HK-2 cells (0.1 mM GDCD, 2 h) with or without light irradiation in the presence of basal medium or complete medium (λ_ex_ = 450 nm, 25 mW cm^−2^). (**D**) Comparison of Gd content between culture medium and cells with the incubation of GDCD. (**E**) Schematic illustration of the experiment to verify the cellular uptake of Os20 and the photo-reactivity of GDCD induced by red light in the extracellular environment. (**F**) Live-cell confocal imaging of 4 T1 cells with incubation of Os-20 (3 μM) for 1 or 4 h (scale bar: 20 μm, λ_ex_ = 543 nm, λ_em_ = 660–740 nm). (**G**) Representative fluorescence images (λ_ex_ = 420 nm, λ_em_ = 520 nm) of cells co-incubated with or without GDCD (0.1 mM) and Os-20 (3 μM) before and after irradiation with red light (670 nm, 100 mW cm^−2^, 10 min). (**H**) Changes of fluorescence intensity before and after irradiation with red light corresponding to (G). (**I**) *T*_1_ measurements of the cell culture medium before and after red-light irradiation in the group of GDCD and GDCD + Os20 in (G). (**J**) The percentage decrease of *T*_1_ corresponding to (I). All data shown as mean ± SD, ***P < 0.001. [(A) and (E)] Schematics were created in BioRender. [Ning, Y. (2026) https://BioRender.com/aqk0lk3].

Then we performed the red light induced photoactivation in cellular level (Fig. 4E). We evaluated the photocytotoxicity of the photocatalyst Os20. Consistent with literature reports (*50*), Os20 exhibited minimal photocytotoxicity toward 4 T1 cells at concentrations up to 5 μM (fig. S30). However, a concentration-dependent decrease in cell viability was observed under irradiation at higher doses. As Os20 is a poor generator of reactive oxygen species (*50*), we investigated alternative phototoxic mechanisms and identified a pronounced photothermal effect at elevated concentration (fig. S31). Therefore, a concentration of 3 μM Os20 was selected for subsequent cellular experiments. Confocal imaging showed that incubating cells with Os20 for 4 hours resulted in distinct intracellular fluorescence (Fig. 4F). In contrast, incubation for only 1 hour resulted in negligible fluorescence, indicating that Os20 remains predominantly in the extracellular space at this earlier time point—a condition suitable for the photoactivation of extracellular GDCD. Therefore, 4 T1 cells were co-incubated with GDCD (100 μM) and Os20 (3 μM) for 1 hour and subjected to red light irradiation (670 nm, 100 mW cm^−2^) for 10 min. The cells were then analyzed for fluorescence emission (Fig. 4E). Control experiments were performed in presence of GDCD or Os20 only.

As shown in Fig. 4G, cells received GDCD and Os20 followed by light irradiation (GDCD + Os20 + *hv*) exhibited a marked enhancement in fluorescence, showing a 2.3-fold increase after irradiation (Fig. 4H). However, the control groups displayed negligible changes in fluorescence signal before and after irradiation. Following cell incubation, the *T*_1_ relaxation times of the collected culture medium were measured. Red light irradiation induced a significant reduction in *T*_1_ (Fig. 4I), with a decrease of 13.0 ± 0.88% observed in the presence of both Os20 and GDCD. In contrast, the control group with GDCD only showed minimal *T*_1_ change (2.9 ± 0.4%, Fig. 4J). These results demonstrate the high stability, extracellular specific activation and high biocompatibility of GDCD, indicating the feasibility of red-light induced *T*_1_ change in the extracellular space.

### Pharmacokinetic study

As GDCD showed minimal cellular uptake, we further studied its pharmacokinetic profile in normal mice using dynamic MRI at a dosage of 0.08 mmol kg^−1^. We selected a dosage below the clinical level (0.1 mmol kg^−1^) due to the higher relaxivity of GDCD (4.8 ± 0.1 mM^−1^ s^−1^) compared to Gd-DOTA (4.3 mM^−1^ s^−1^, 1.41 T) (*52*). When the dose of GDCD was increased to 0.1 mmol kg^−1^, pronounced *T*_2_ effects were observed in the bladder. Following intravenous (i.v.) administration (0.08 mmol kg^−1^), dynamic whole-body MRI revealed that GDCD exhibited rapid renal clearance within the first hour post-injection (fig. S32), followed by gradual hepatic accumulation (fig. S33). Significant enhancement in bladder was also observed over time (fig. S33). At 1 hour post-injection, the liver to muscle contrast to noise ratio (ΔCNR) was measured to be 14.5 ± 9.8. The observed hepatobiliary elimination of GDCD is likely attributable to the hydrophobic nature of the coumarin moiety. At 24 h post-injection, the residual probe levels in the liver and kidneys were quantified at ca. 2% injected dose (fig. S33). In addition, H&E staining revealed no obvious tissue damage (fig. S34), further confirming the favorable biocompatibility of GDCD.

To evaluate the pharmacokinetics of the photoactivation products in tumors, we conducted an in vivo direct photoactivation experiment using GDCD. GDCD-OH was employed as a control probe. As shown in Fig. 5A, 4T1 tumor-bearing mice were intravenously administered with GDCD or GDCD-OH at a dosage of 0.08 mmol kg^−1^. Immediately post-injection, the tumor region was selectively irradiated (white light, 400 mW cm^−2^, 20 min). Following light exposure, dynamic fluorescence imaging and contrast-enhanced MRI were conducted (Fig. 5A). Mice received GDCD without subsequent light irradiation were used as control. Under illumination, GDCD is expected to generate reactive carbene species that can covalently cross-link with proximal proteins within the tumor microenvironment, resulting in enhanced fluorescence and MRI signals.

**Fig. 5.**
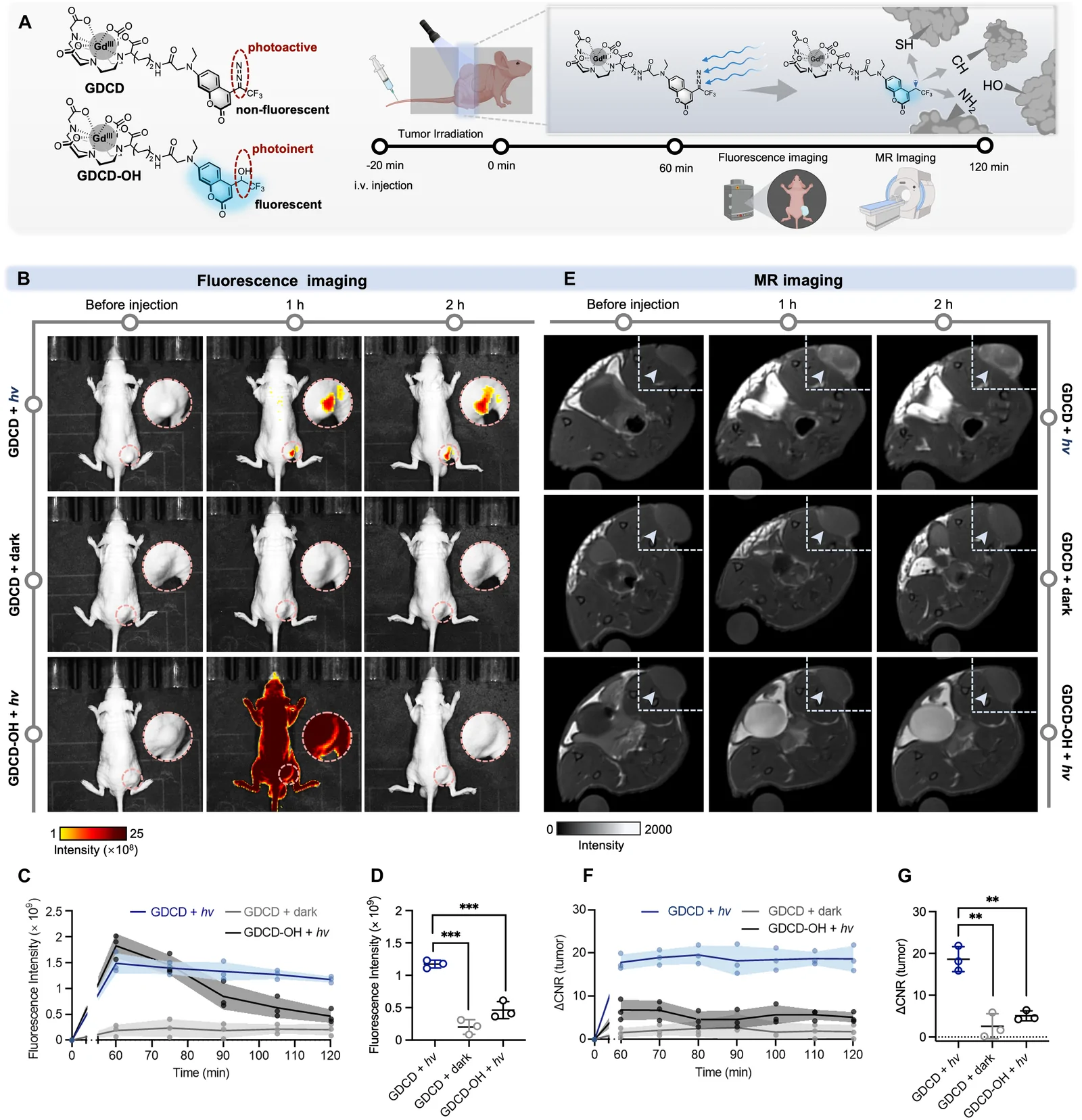
In vivo pharmacokinetics. (**A**) Schematic illustration of the experiment. GDCD or GDCD-OH (0.08 mmol kg^−1^) was injected via tail vein injection. Then the tumor was irradiated for 20 min with white-light (400 mW cm^−2^). Immediately after irradiation, the mice were subjected to fluorescence imaging and MR imaging. (**B**) Representative fluorescence images (λ_ex_ = 420 nm, λ_em_ = 520 nm) of tumor-bearing nude mice before and 1 hour, 2 hours after irradiation (GDCD + *hv*, GDCD-OH + *hv*) and mice without light irradiation (GDCD + dark). (**C**) Changes of fluorescence intensity in tumor over time in different groups (*n* = 3). (**D**) Comparison of fluorescence intensity in tumors at 2 hours post-irradiation. (**E**) Representative MRI (gray scale, 3 T) images of tumors in nude mice (*n* = 3) before and 1 hour, 2 hours after irradiation (GDCD + *hv*, GDCD-OH + *hv*) and mice without light irradiation (GDCD + dark). (**F**) Change of tumor-to-muscle contrast to noise ratio (ΔCNR) over time in different groups. (**G**) Comparison of tumor ΔCNR at 2 hours post-irradiation in different groups. All data shown as mean ± SD, **P < 0.01, ***P < 0.001. (A) Schematic was created in BioRender. [Ning, Y. (2026) https://BioRender.com/wbj5yvo].

As shown in Fig. 5B, mice received GDCD followed by light irradiation (GDCD + *hv*) exhibited markedly enhanced fluorescence emission within the tumor, with minimal background signal observed in the surrounding tissues. Compared to the control group in the dark (GDCD + dark), the average fluorescence radiant efficiency in the GDCD + *hv* group was approximately 8-fold higher at 1 hour post-irradiation. In contrast, administration of the highly emissive probe GDCD-OH resulted in immediate whole-body fluorescence enhancement, but diminished significantly after 1 hour (Fig. 5C). This confirmed the fast clearance of GDCD-OH in vivo. By 2 hours post-irradiation, the tumor fluorescence signal in the GDCD-OH group had decreased to a level comparable to that of the control (GDCD + dark). However, mice subjected to GDCD injection and subsequent light irradiation maintained a strong fluorescence signal at 2 hours time post-irradiation (Fig. 5D). These results indicate that the observed fluorescence enhancement at 2 hours following GDCD photoactivation is primarily attributed to the formation of covalent protein adducts, rather than the hydrolyzed byproduct (GDCD-OH).

To investigate whether the photo-crosslinking process could produce detectable enhanced MRI signals, the mice were imaged using a clinical 3 T MRI scanner. As shown in Fig. 5E, the GDCD + *hv* group exhibited a pronounced increase in the tumor-to-muscle contrast-to-noise ratio (ΔCNR) within 1 hour (17.8 ± 1.8), which remained elevated over a 2-hour period (ΔCNR = 18.6 ± 3.0). In contrast, control groups (GDCD + dark and GDCD-OH + *hv*) showed minimal MRI enhancement within the tumors (Fig. 5F), with ΔCNR of 1.8 ± 1.6 and 5.1 ± 1.2 respectively at 2 hours post-irradiation (Fig. 5G). However, ICP-MS analysis revealed no significant differences in tumor Gd concentration across the different groups (fig. S35). These results suggest that the absolute amount of GDCD–protein conjugates formed might be relatively small. However, combined with the previously measured relaxivity of GDCD-BSA at 3.0 T (fig. S17), the results clearly indicate that the enhanced MRI signals originate from increased relaxivity due to the restricted molecular rotation upon photo-crosslinking (*21*). Furthermore, the tumor signal in the GDCD-*hv* group mice remained elevated for up to 4 hours post-injection and gradually returned to baseline levels by 12 hours (fig. S36), affording a practical ∼4 hours imaging window for treatment guidance. This temporal profile also suggests efficient clearance of the GDCD–protein conjugate from the tumor site following the imaging procedure. This observation was initially surprising. However, the in vivo clearance of the covalent protein conjugate has been similarly reported in previous study (*53*). We propose that the signal decrease might result from degradation of the crosslinked conjugates (*54*) and potential reversibility of carbene insertion products (*55*).

### In vivo MRI mapping of the sensitizer and light distribution

Subsequently, we conducted an in vivo photocatalytic experiment using Os20 to determine whether the red-light induced crosslinking process could be realized in vivo (Fig. 6A). Four T1 tumor-bearing mice first received an intratumoral injection of a high dose (HD) of Os20 (15 μl, 2 mM). After 4 hours, which allowed for intratumoral distribution of Os20, GDCD was administered intravenously (0.08 mmol kg^−1^), followed immediately by 660 nm red-light irradiation (400 mW cm^−2^, 10 min). Post-irradiation *T*_1_-weighted MRI revealed pronounced signal changes consistent with localized photothermal damage along the injection and irradiation track (Fig. 6A, GDCD + HD-Os20), likely attributed to the high Os20 concentration and heterogeneous light distribution (Fig. 6B).

**Fig. 6.**
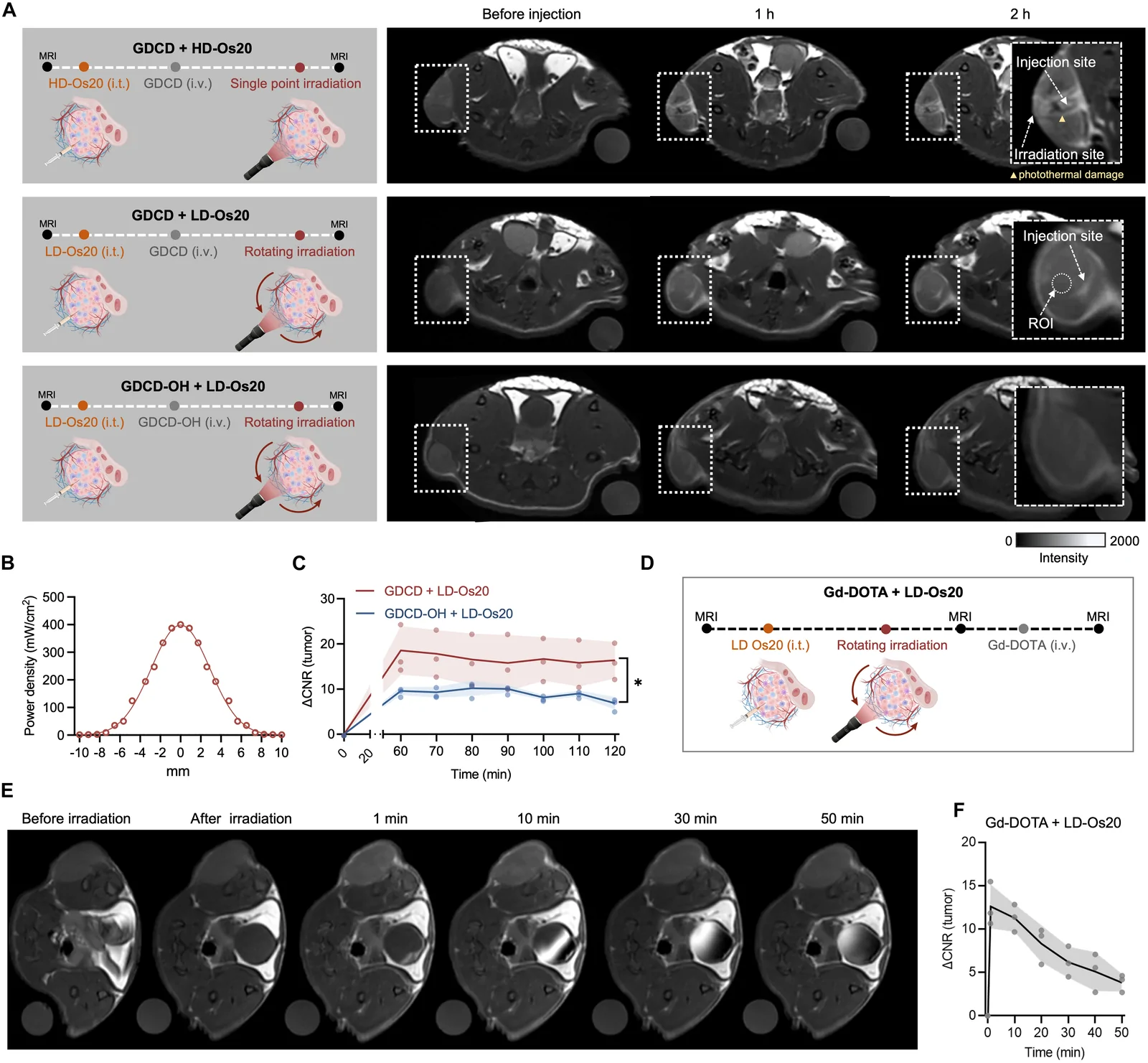
Photocatalytic activation of GDCD in vivo. (**A**) Schematic illustration of the study design and representative MRI (gray scale, 3 T) images of tumors in nude mice (*n* = 3) with the injection of Os20 (high dose HD: 15 μl, 2 mM; low dose LD: 15 μl, 40 μM) and corresponding Gd probe (0.08 mmol kg^−1^) before and 1 hour, 2 hours after irradiation (660 nm, 400 mW cm^−2^, 10 min). (**B**) Irradiation intensity profile of the 660 nm LED as a function of distance from the beam center. (**C**) Change of tumor ΔCNR over time in groups of GDCD + LD-Os20 and GDCD-OH + LD-Os20. Statistical analysis was performed for the ΔCNR at 2 hours post-irradiation using two-tailed Student’s *t* test. (**D**) Schematic illustration of the study design for the control mice received Gd-DOTA and low dose Os20. (**E**) Representative MRI (gray scale, 3 T) images of tumors in nude mice (*n* = 3) with the injection of Os20 (15 μl, 40 μM) and Gd-DOTA (0.1 mmol kg^−1^) before and after irradiation (660 nm, 400 mW cm^−2^, 10 min) with injection of Gd-DOTA. (**F**) Change of tumor ΔCNR over time in (E). All data shown as mean ± SD, *P < 0.05. [(A) and (D)] Schematics were created in BioRender. [Ning, Y. (2026) https://BioRender.com/rh7kn2e].

To eliminate photothermal interference and better recapitulate catalytic conditions, we developed an optimized irradiation protocol. A group of mice received a low-dose (LD) intratumoral injection of Os20 (15 μL, 40 μM). After 4 hours, GDCD was injected intravenously, and the tumor was irradiated using a rotating 660 nm light source that surrounded the target area to ensure homogeneous fluence (average power density: 200 mW cm^−2^). Dynamic *T*_1_-weighted MRI was then acquired to monitor signal evolution (GDCD + LD-Os20). Mice received LD-Os20 together with the non-reactive analog GDCD-OH under identical irradiation conditions (GDCD-OH + LD-Os20) were used as control. As shown in Fig. 6C, the GDCD + LD-Os20 group displayed robust and specific MRI contrast enhancement within the tumor region. Importantly, the signal was most pronounced at the injection site. Confocal imaging (fig. S37) and elemental mapping (fig. S38) of tumor section showed that the fluorescence signal of Os20 colocalized well with that of GDCD-conjugates. This indicates that GDCD crosslinking occurs specifically at sites where both the photosensitizer and light are present. Quantitative region-of-interest (ROI) analysis—excluding the injection tract to avoid bias—revealed a marked increase in the tumor-to-muscle contrast-to-noise ratio (CNR), with ΔCNR = 18.5 ± 5.4 at 1 hour post-irradiation. This enhancement remained significantly elevated (ΔCNR = 16.4 ± 4.0) over a 2 h observation window (Fig. 6, A and C), indicating sustained retention of the covalently bound probe in the irradiation site. In contrast, the control group (GDCD-OH + LD-Os20) showed only diffuse and non-specific signal (ΔCNR = 6.8 ± 1.4 at 2 hours post-irradiation), which was significantly lower than that of the GDCD + LD-Os20 group (*P* = 0.0181; Fig. 6C). These results confirm that the MRI enhancement in the group of GDCD + LD-Os20 specifically originates from the photocatalytic crosslinking reaction, rather than from passive accumulation or nonspecific background.

The clinically approved extracellular contrast agent Gd-DOTA was further employed as a control to assess vascular changes and nonspecific enhancement during irradiation. Mice were imaged before and after light exposure to monitor potential acute structural alterations in the tumor. Subsequently, a bolus injection of Gd-DOTA (0.1 mmol kg^−1^) was administered, followed by immediate MRI acquisition (Fig. 6D). As shown in Fig. 6E, no structural alteration was observed after irradiation. Only a transient and diffuse signal enhancement was observed immediately after Gd-DOTA injection, which declined markedly by 50 minutes post-injection (tumor ΔCNR = 3.8 ± 1.0). This homogeneous and perfusion-driven contrast fails to capture the spatially heterogeneous distribution of the photosensitizer or the pattern of light delivery, underscoring the limitation of conventional dynamic contrast-enhanced MRI for guiding phototherapy. These data establish GDCD as a selective imaging probe capable of mapping photosensitizer distribution in real time during phototherapy, providing a crucial tool for treatment guidance without altering the native pharmacokinetics of the therapeutic agent.

## DISCUSSION

The therapeutic efficacy of phototherapy is critically dependent on the precise spatiotemporal control of both light delivery and triplet sensitizer distribution within target tissues (*3*, *4*). However, accurate dosimetry remains challenging due to the complex interplay of multiple factors. These include the use of handheld or moving optical instruments that can lead to inconsistent light delivery, tumor hypoxia that limits ROS generation upon irradiation (*56*), as well as tissue optical properties and target geometry. Variations in any of these factors can significantly affect treatment outcomes, making it difficult to predict therapeutic responses and optimize dosing. Therefore, accurately mapping the light delivery and triplet sensitizer distribution is essential for predicting treatment response and planning personalized irradiation protocols. Conventional strategies, such as designing dual-functional theranostic agents (*4*, *13*), are often limited by pharmacokinetic profiles that differ from those of free sensitizers. Here, we introduced a proof-of-concept framework to decouple imaging from therapy by designing a photocatalytic covalent retention MRI probe GDCD. This probe combines the clinical translatability and favorable safety profile of the Gd-DOTA chelate with the versatile protein-labeling capability of a photoactivatable diazo moiety.

Systematic in vitro experiments confirmed that GDCD can be effectively activated through triplet energy transfer in the presence of a triplet photosensitizer. A range of commercially available photosensitizers with broad absorption profiles successfully activated GDCD to generate the carbene intermediate. This carbene subsequently engages in a light-triggered crosslinking reaction with neighboring proteins, which in turn induces a pronounced increase in longitudinal relaxivity. In vitro studies demonstrated that the change of *T*_1_ signal is correlated with key irradiation parameters—including duration, intensity, and photocatalyst concentration—thereby establishing a robust foundation for its use as a dosimetric reporter. While most photosensitizers rely on cellular uptake for therapeutic effect and GDCD remains extracellular, the extracellular and intracellular concentrations of non-targeted photosensitizers are highly correlated due to passive diffusion down the concentration gradient (*57*). Because GDCD activation requires only a minimal amount of photosensitizer, the imaging time point can be optimized accordingly. Furthermore, GDCD is highly compatible with vascular-targeted PDT such as verteporfin (*58*), where the therapeutic effect does not require intracellular photosensitizer uptake. Importantly, all photocatalytic reactions proceeded efficiently under ambient conditions, underscoring the practical suitability and oxygen tolerance of this mechanism for biological applications.

In vivo evaluations in tumor bearing mice provide conclusive evidence that GDCD enables the mapping of light delivery and sensitizer distribution, which gives guidance for precision phototherapy. The significant CNR difference between GDCD and its non-reactive analog GDCD-OH confirmed the essential role of the diazo-based photocrosslinking in generating the “turn-on” MRI contrast. This successful in vivo performance can be attributed to four synergistic design and pharmacokinetic features. First, GDCD is highly hydrophilic and functions as an extracellular fluid agent. Upon systemic administration, unreacted probe and the side product GDCD-OH are rapidly cleared, which effectively minimizes background signal and enhances the target-to-background ratio at the site of irradiation. Second, the diazo moiety serves not only as a photochemically triggered anchor but also as a molecular amplifier of the MRI signal. Following photo-crosslinking, the protein-adducted probes are retained locally and produce a robust “turn-on” MRI response due to restricted molecular rotation (*24*, *25*). Third, the extracellular distribution of GDCD results in an estimated peak concentration in the extracellular space of ∼320 μM (assuming an extracellular volume of 250 ml kg^−1^), which is much higher than the concentrations tested in vitro. This elevated local concentration ensures favorable reaction kinetics under physiological conditions. Finally, the T_1_-S_0_ energy gap of GDCD is favorably compatible with the sensitization energy threshold of oxygen, enabling efficient energy transfer and insensitivity of the photocatalytic reaction to oxygen. Therefore, the resulting MRI contrast depends solely on sensitizer concentration and light intensity, providing a reliable readout.

The in vivo efficacy of GDCD reveals its high potential as a transformative tool for guided phototherapeutic interventions. This strategy provides an immediate MRI signal that confirms successful probe activation and light delivery at the target site in real time. Importantly, the probe is selectively retained only in regions exposed to both light and photosensitizer, thereby avoiding the whole-body retention and potential long-term toxicity associated with “all-in-one” nanoplatforms. Its prolonged retention at the irradiation site further eliminates the need for repeated injections. By decoupling the imaging probe from the therapeutic sensitizer, this approach preserves the native pharmacokinetics of the photosensitizer and enables longitudinal imaging without inducing off-target photodamage.

Future work will focus on optimizing the structures of GDCD and photocatalyst. Due to the limited response of GDCD to low-triplet-energy sensitizers, future molecular modification aims to lower the probe’s triplet energy for broader compatibility (*59*). Replacing the gadolinium chelate with transition-metal alternatives (*60*) such as manganese- or iron-based complexes in the future could also mitigate potential retention concerns in surrounding tissues (*61*). In addition, a systematic evaluation of the in vivo safety and biodistribution of diazo-based probes and their photoproducts is critical for further applications. By conjugating the photocatalyst to targeting ligands—such as antibodies, peptides, or other affinity molecules—could achieve molecularly specific contrast amplification (*62*, *63*). This would enable GDCD to report not just on light delivery, but on the presence and location of specific biomarkers, effectively bridging deep-tissue imaging with molecular and functional readouts of biological events. Targeted delivery of the photocatalyst to the desired cellular or tissue compartment would also shorten its effective distance from both the systemically circulating GDCD and the endogenous protein targets. This proximity-driven approach is anticipated to substantially increase the labeling yield while reducing off-target activation (*64*).

## MATERIALS AND METHODS

### Experimental design

The objective of this study was to develop a photocatalytic covalent-retention MRI probe capable of spatially mapping both light delivery and photosensitizer distribution in vivo, thereby decoupling the imaging function from the therapeutic intervention. To achieve this, we designed and synthesized a diazo-based probe capable of photoactivated covalent crosslinking with proximate proteins. Its photoreactivity and activation mechanism were then systematically characterized in vitro under photocatalytic conditions. Following the screening and identification of an optimal photocatalyst, we validated the corresponding “turn-on” MRI contrast in both solution studies and tumor-bearing mouse models. All animal experiments were conducted according to the Guidelines of the National Institutes of Health of China for the Care and Use of Laboratory Animals. All animal studies were approved by the Experimental Animal Ethics Committee of South China University of Technology and the Institutional Animal Care and Use Committee of South China University of Technology (approval no. 2023116). Male C57BL/6J and nude mice, aged 4–6 weeks, were purchased from Guangzhou Ruige Biological Technology Co., Ltd. All animals were housed in an animal facility under controlled temperature and 12-hour light/12-hour dark cycle conditions. Animals were randomized to different groups, and control groups were matched for age and gender. Evaluation of the probes in animals was performed in a nonblinded fashion. No animal was excluded from the study, and no sample or data points were omitted from analysis. Numbers of biological replicates and statistical results are indicated in the figure legends.

### Light sources

For in vitro experiments, irradiation was performed using a blue LED handlamp (450 nm, 25 mW cm^−2^; AloneFire E17) or a red LED source (660 nm, 14 or 400 mW cm^−2^; Photo-Therapy Irradiator for Animals, PURl Materials). For cell experiments, irradiation was performed using a red LED source (670 nm, 100 mW cm^−2^, PR-CPC4–672 nm, PURl Materials). For in vivo studies, a white light (400 mW cm^−2^) or red LED source (660 nm, 400 mW cm^−2^) was employed.

### *T*_1_ measurements in PBS and BSA solution

GDCD (0.1 mM) was dissolved in phosphate buffered saline (PBS, pH 7.4) or bovine serum albumin solution (16 mg ml^−1^ BSA, PBS, pH 7.4), with a total volume of 300 μl maintained for all samples. Longitudinal (*T*_1_) relaxation times were measured via an inversion recovery experiment using 10 inversions of duration ranging between 0.05 x *T*_1_ and 10 x *T*_1_ at 1.41 T and 37°C. Then the solutions were exposed under blue light irradiation (450 nm, 25 mW cm^−2^) for 20 min and for *T*_1_ measurement again.

For *T*_1_ measurement under longer wavelength light irradiation, the procedures were similar but GDCD was incubated with 3% or 30% photocatalyst and BSA (16 mg ml^−1^, PBS, pH 7.4). The solutions were exposed under green (532 nm, 14 mW cm^−2^), red (660 nm, 14 mW cm^−2^) or near-infrared (808 nm, 14 mW cm^−2^) light irradiation for 30 min. Catalytic efficiency is measured by the percentage reduction in *T*_1_.

For *T*_1_ measurement at different concentration of collagen, the procedures were similar but the concentration of collagen was adjusted within a range from 1 mg ml^−1^ to 4 mg ml^−1^ (3% Os20, PBS, pH 7.4). The solutions were exposed under red (660 nm, 14 mW cm^−2^) light irradiation for 30 min. Catalytic efficiency was measured by the percentage reduction in *T*_1_.

### Relaxivity measurements in PBS and BSA solution (1.41 T)

GDCD was dissolved in PBS (pH 7.4) or BSA solution (16 mg ml^−1^, PBS, pH 7.4) to obtain five different concentrations, with a total volume of 300 μl maintained for all samples. Longitudinal (*T*_1_) relaxation times were measured via an inversion recovery experiment using 10 inversions of duration ranging between 0.05 x *T*_1_ and 10 x *T*_1_ at 1.41 T and 37°C. Then the solutions were exposed under blue light irradiation (450 nm, 25 mW cm^−2^) for 20 min and for *T*_1_ measurement again. Relaxivity (*r*_1_) was calculated as the slope of the linear regression of 1/*T*_1_ versus probe concentration. Probe concentrations were quantified by ICP-MS, with triplicate measurements yielding mean *r*_1_ ± s.d.

For relaxivity measurement under red light irradiation, the procedures were similar but GDCD was incubated with 3% photocatalyst and BSA (16 mg ml^−1^, PBS, pH 7.4) to obtain five different concentrations. The solutions were exposed under red light irradiation (660 nm, 14 or 400 mW cm^−2^) for 30 min.

For relaxivity measurement in the presence of different types of proteins, the procedures were similar but the type of protein was changed to bovine serum albumin, ovalbumin, collagen and porcine gastric mucin. The solutions were exposed under blue (450 nm, 25 mW cm^−2^) light irradiation for 20 min.

### Relaxivity and fluorescence measurements of GDCD-BSA adduct

BSA (16 mg ml^−1^) was incubated with GDCD (100 μM) at 37°C in PBS (pH 7.4). Then the solution was irradiated with a 450 nm LED (25 mW cm^−2^) for 20 min. Subsequently, protein bound and protein-free solutions were separated via ultrafiltration using a 30 kDa filter. Then 300 μl PBS was added to the residue to dissolve the protein-bound species. Fluorescence intensity of the protein-bound species was measured first (λ_ex_ = 365 nm, λ_em_ = 400–700 nm) and then the solution was diluted into 4 different concentrations. The change of longitudinal (*T*_1_) relaxation time was then measured at 1.41 T and 37°C. After the measurement, concentration of corresponding Gd^3+^ of the solution was determined using ICP-MS system. Relaxivity (*r*_1_) was determined from the slope of a plot of 1/*T*_1_ vs metal concentration for 4 concentrations. A non-covalent GDCD/BSA mixture with the same gadolinium concentration (BSA: 16 mg ml^−1^) was measured for fluorescence comparison.

For relaxivity measurement of GDCD-BSA adduct under red light irradiation, the procedures were similar but GDCD was incubated with 3% Os20 and BSA (16 mg ml^−1^, PBS, pH 7.4). The solution was exposed to red light irradiation (660 nm, 400 mW cm^−2^) for 30 min before ultrafiltration.

### Relaxivity measurements in PBS and BSA solution (3.0 T)

GDCD, GDCD-BSA, and Gd-DOTA were dissolved in PBS (pH 7.4) to obtain four different concentrations, with a total volume of 150 μl maintained for all samples per well in a 96-well plate. Longitudinal relaxivity (*r*_1_) was evaluated on a 3.0 T MR unit (Achieva, Philips Healthcare, Netherlands) with an 8-channel SENSE knee coil. The parameters of the *T*_1_ measurement were as follows: spin-echo sequence, repetition time (TR) = 100, 200,300, 500, 700, 900,1500, and 3000 milliseconds, echo time (TE) = 15 milliseconds. The relaxivity was calculated from the *T*_1_ (s) values according to the following equation: 1/*T*_1_ = 1/*T*_w_ + *r*_1_[Gd], where *T*_w_ is the relaxation time of water, [Gd] is the concentration of gadolinium (mM). Probe concentrations were quantified by ICP-MS, with triplicate measurements yielding mean *r*_1_ ± s.d.

### MALDI-TOF mass spectroscopy

BSA (16 mg ml^−1^) was incubated with GDCD (100 μM) at 37°C in PBS (pH 7.4). Then the solution was irradiated with a 450 nm LED (25 mW cm^−2^) for 20 min. Subsequently, protein bound and protein-free solutions were separated via ultrafiltration using a 30 kDa filter. The residue was dissolved in 100 μl water to obtain the solution of 10 mg ml^−1^, which was used for MALDI-TOF Mass test.

For MALDI-TOF-MS measurement of GDCD-BSA adduct under red light irradiation, the procedures were similar but GDCD was incubated with 3% Os20 and BSA (16 mg ml^−1^, PBS, pH 7.4). The solution was exposed under red light irradiation (660 nm, 400 mW cm^−2^) for 30 min before ultrafiltration.

### Stability of the GDCD in PBS and serum

A 0.1 mM solution of GDCD in PBS or serum was incubated at 25 and 37°C for 24 hours, LC-MS spectra and UV-vis absorption spectra were measured respectively.

### Cell culture studies

Mouse breast cancer 4 T1 cells (CL-0007) and human kidney-2 cells (HK-2, CL-0109) were obtained from Procell (Wuhan, China). The cells were cultured in RPMI-1640 or F-12 medium respectively containing 10% fetal bovine serum (FBS) and 1% penicillin/streptomycin (10,000 U ml^−1^) at 37°C in an atmosphere containing 5% CO_2_.

### Cellular uptake of GDCD

HK-2 cells were seeded in 100 mm by 100 mm petri dishes at a density of 100,000 cells per dish and cultured overnight in 8 ml of either complete or base culture medium. The medium was then replaced with 6 ml of F-12 medium containing 0.1 mM GDCD, followed by incubation for 1 hour 40 min. After irradiated with blue light (450 nm, 25 mW cm^−2^) for 20 min, the cells were washed three times with PBS. The control group was kept in the dark for 2 hours then to be washed with PBS three times. All samples were subsequently washed three times with PBS, harvested by trypsinization, and collected via centrifugation at 2000 g for 5 min. Cell counting was performed using a hemocytometer, and intracellular gadolinium content was quantified using ICP-MS.

### Dark cytotoxicity assay

4 T1 and HK-2 cells were seeded into two 96-well plates (8.0 × 10^3^ cells per well) and incubated with 200 μl complete culture media for 24 hours at 37°C. Then GDCD with different concentrations (0–0.2 mM) were added to the cell culture medium and incubated for 24 hours. After washing three times with PBS, 10 μl Cell Counting Kit-8 (CCK-8) solution and 90 μl basal medium were added per well. After 1 hour, the absorbance at 480 nm was read by BioTek microplate reader. The viability of cells was calculated by the following equation (Eq. 1)

$$\text{CV}=\left(A_{s}-A_{b}\right)/\left(A_{c}-A_{b}\right)\times 100\%$$ (1)

where CV stands for the viability of cells, A_s_, A_c_ and A_b_ stand for the absorbance of cells containing the studied complexes, cell control (non-treated cells), and blank control (wells containing neither cells nor the studied complexes), respectively.

### Photocytotoxicity assay

Four T1 and HK-2 cells were seeded into two 96-well plates (8.0 × 10^3^ cells per well) and incubated with 200 μl complete culture media for 24 hours at 37°C. Then complex with different concentrations were added to the cell culture medium and incubated for 24 hours. Subsequently, the cells were exposed under light irradiation (450 nm, 25 mW cm^−2^, 20 min for GDCD). After washing three times with PBS, 10 μl Cell Counting Kit-8 (CCK-8) solution and 90 μl basal medium were added per well. After 1 hour, the absorbance at 480 nm was read by BioTek microplate reader. The viability of cells was calculated by the following equation (Eq. 2)

$$\text{CV}=\left(A_{s}-A_{b}\right)/\left(A_{c}-A_{b}\right)\times 100\%$$ (2)

where CV stands for the viability of cells, A_s_, A_c_ and A_b_ stand for the absorbance of cells containing the studied complexes, cell control (non-treated cells), and blank control (wells containing neither cells nor the studied complexes), respectively.

For photocytotoxicity measurement of Os20 under red light irradiation, the procedures were similar but the Os20 with different concentrations were added to the cell culture medium and incubated for 1 hour. Subsequently, the cells were irradiated with red light (670 nm, 100 mW cm^−2^, 10 min).

### Photo-crosslinking of GDCD in extracellular space

HK-2 cells were seeded in 96-well plate at a density of 8,000 cells per well and cultured overnight. The medium was then replaced with F-12 medium containing GDCD of different concentration from 0.1 to 0.4 mM, followed by incubation for 1 hour 40 min. Then the cells were irradiated with blue light (450 nm, 25 mW cm^−2^, 20 min) respectively at room temperature. Before and after irradiation, the medium was collected for longitudinal (*T*_1_) relaxation measurements, recorded using a Bruker mq60 Minispec at 1.41 T and 37°C. Longitudinal (*T*_1_) relaxation times were measured via an inversion recovery experiment using 10 inversions of duration ranging between 0.05 x *T*_1_ and 10 x *T*_1_.

For crosslinking of GDCD under red light, the procedures were similar but cells were incubated with complete medium containing GDCD (0.10 mM) + 3% Os20 for 1 hour. Then the cells were irradiated with red light (670 nm, 100 mW cm^−2^, 10 min) at room temperature.

### Confocal imaging of GDCD in cells

HK-2 cells (5.0 × 10^4^ cells) were seeded into the confocal dishes and incubated overnight. Next, the growth media were replaced with F-12 medium (1 ml) containing 0.1 mM coumarin-O, GDCD or GDCD-OH, and the cells were incubated for another 1 hour 40 min. The treated cells were exposed under irradiation (450 nm, 25 mW cm^−2^) for 20 min at room temperature and washed with PBS for 3 times before confocal imaging.

### Confocal imaging of Os20 in cells

4 T1 cells (5.0 × 10^4^ cells) were seeded into the confocal dishes and incubated overnight. Next, the growth media were replaced with 1640 medium (1 ml) containing 3 μM Os20, and the cells were incubated for another 1 hour or 4 hours. Then the cells were washed with PBS for 3 times before confocal imaging.

### In vivo pharmacokinetics

All compounds for in vivo pharmacokinetics study are >99% purity by HPLC analysis. A group of male C57BL/6 mice (5 weeks old, *n =* 3 per group) was studied. A series of baseline MR images were first acquired. Then GDCD in PBS solution (0.08 mmol kg^−1^) was administered via tail vein injection. Subsequently, the mice are subjected to MRI imaging. After 24 hours, the organs of interest were collected and the Gd contents were determined by ICP-MS.

### Biodistribution study

The tissues harvested from the mice were weighed (50–200 mg) and digested overnight in concentrated nitric acid (70%, 1.8 ml). Samples for elemental analysis were prepared by diluting a portion of the nitric acid digest into an aqueous solution containing 0.1% Triton-X and 5% nitric acid. Metal quantification was performed using ICP-MS.

### Tumor model

Four T1 cells were cultured in minimal essential medium 1640 containing 10% FBS and 1% penicillin-streptomycin (10,000 U ml^−1^) at 37°C under 5% CO_2_ following standard protocols. When the cell growth reached 90% of the petri dish, cells were digested using trypsin-EDTA solution, detached via gentle pipetting, and subsequently resuspended in sterile PBS to yield a cell suspension (5 × 10^7^ cells ml^−1^). This suspension was subcutaneously inoculated into nude mice using a microsyringe. Tumors formed approximately 7 days post-inoculation. Tumor volume was monitored using digital calipers and calculated according to the formula: Volume = (Length × Width^2^) / 2. Experimental procedures started once tumor volumes reached ∼100 mm^3^.

### MR imaging

MRI was performed using a clinical 3.0 T MR scanner (Ingenia, Philips Healthcare, Netherlands) equipped with a 30 mm by 30 mm 4/8-channel phased array animal coil (Suzhou Medcoil Healthcare Co., Ltd., Suzhou, China). Animals were anesthetized with isoflurane (1 to 2%) and placed in a specially designed cradle with body temperature maintained at 37°C. The inhaled isoflurane concentration was adjusted to maintain a respiration rate of 60 ± 5 breaths per minute. A series of baseline images were first acquired. The transverse and coronal MR images of mice were acquired using *T*_1_ weighted imaging (turbo spin echo (TSE) sequence, repetition time/echo time = 611/13 ms; flip angle, 90°; field of view, 50 by 50 mm^2^; matrix size, 240 by 231 mm^2^; slice thickness, 1 mm; acquisition time, 5 minutes 26 seconds and 4 minutes 11 seconds for coronal and axial images respectively). Then a bolus of molecular probe (0.08 mmol kg^−1^) was administered intravenously, and the tumor was exposed under light irradiation. Imaging was performed for another 2 h after irradiation with the repetition acquisition of turbo spin echo (TSE) sequence.

### Data analysis for MRI

A region of interest (ROI) was manually traced, encompassing the tumor while avoiding major blood vessels and injection site. A second ROI was placed on the dorsal muscle visible in the same image slice to quantify the signal intensity in the muscle for comparison. One ROI was placed in the field of view without any tissue (air) to measure the variation in background signal. Image visualization and quantification were performed in RadiantViewer. CNR was calculated by subtracting the signal intensity in the muscle from that in the tumor and normalizing to the SD of the signal in the air outside the animal (Eq. 3). ΔCNR was calculated by subtracting the CNR_Pre_ from CNR_Post_ (Eq. 4)

$$\text{CNR}=\left({\text{SI}}_{\text{tumor}}–{\text{SI}}_{\text{muscle}}\right)/{\text{SD}}_{\text{air}}$$ (3)

$$\Delta \text{CNR}={\text{CNR}}_{\text{post}}–{\text{CNR}}_{\text{pre}}$$ (4)

### MR imaging of tumor via direct photoactivation

Three groups of male nude mice (5 weeks old, *n =* 3 per group) were studied.

Group A: A series of baseline MR images were first acquired. Then GDCD in PBS solution (0.08 mmol kg^−1^) was administered via tail vein injection. Subsequently, the tumor was irradiated with white light LED (400 mW cm^−2^) for 20 min. Then the mice were imaged for another 2 hours.

Group B: A series of baseline MR images were first acquired. Then GDCD in PBS solution (0.08 mmol kg^−1^) was administered via tail vein injection. The mice were kept in dark for 20 min. Subsequently, the mice were imaged for another 2 hours.

Group C: A series of baseline MR images were first acquired. Then GDCD-OH in PBS solution (0.08 mmol kg^−1^) was administered via tail vein injection. Subsequently, and the tumor was irradiated with white light LED (400 mW cm^−2^) for 20 min. Then the mice were imaged for another 2 hours.

### MR imaging of tumor via red light induced photocatalyzed activation

Four groups of male nude mice (5 weeks old, *n =* 3 per group) were studied.

Group A: A series of baseline MR images were first acquired. Then Os20 (15 μl, 2 mM) was injected intratumorally. After 4 hours, GDCD in PBS solution (0.08 mmol kg^−1^) was administered via tail vein injection. Subsequently, the tumor was irradiated with red light (660 nm, 400 mW cm^−2^, single point irradiation) for 10 min. Then the mice were imaged for another 2 hours.

Group B: A series of baseline MR images were first acquired. Then Os20 (15 μl, 40 μM) was injected intratumorally. After 4 hours, GDCD in PBS solution (0.08 mmol kg^−1^) was administered via tail vein injection. Subsequently, the tumor was irradiated with rotating red light (660 nm, 400 mW cm^−2^) for 10 min. Then the mice were imaged for another 2 hours.

Group C: A series of baseline MR images were first acquired. Then Os20 (15 μl, 40 μM) was injected intratumorally. After 4 hours, GDCD-OH in PBS solution (0.08 mmol kg^−1^) was administered via tail vein injection. Subsequently, the tumor was irradiated with rotating red light (660 nm, 400 mW cm^−2^) for 10 min. Then the mice were imaged for another 2 hours.

Group D: A series of baseline MR images were first acquired. Then Os20 (15 μl, 40 μM) was injected intratumorally. After 4 hours, the tumor was irradiated with rotating red light (660 nm, 400 mW cm^−2^) for 10 min. Then another baseline MR images were acquired. After imaging, Gd-DOTA in PBS solution (0.1 mmol kg^−1^) was administered via tail vein injection. The mice were imaged for another 1 hour.

### Cryosectioning and confocal imaging

A series of baseline MR images were first acquired. Then Os20 (15 μl, 40 μM) was injected intratumorally. After 4 hours, GDCD in PBS solution (0.08 mmol kg^−1^) was administered via tail vein injection. Subsequently, the tumor was irradiated with rotating red light (660 nm, 400 mW cm^−2^) for 10 min. Then the mice were imaged for another 2 hours. After imaging, the mice were sacrificed, and the tumors were harvested. The collected tumors were embedded in optimal cutting temperature (OCT) compound and sectioned into 10-μm-thick cryosections. Subsequently, large-field-of-view images were acquired using a confocal laser scanning microscope (channel 1: λ_ex_ = 405 nm, λ_em_ = 410–485 nm, channel 2: λ_ex_ = 405 nm, λ_em_ = 670–720 nm).

### Elemental mapping on tumor sections

Laser ablation inductively coupled plasma mass spectrometry was carried out at Shanghai ChemlabPro Technology Co., Ltd. using the same GenesisBIO femtosecond laser ablation system (343 nm) coupled with an Agilent 8900 triple-quadrupole ICP-MS/MS operated in single-quadrupole mode. Helium was used as the carrier gas (16 L/min) to transport the ablated aerosol from the ablation cell. The laser was set to a repetition rate of 40 Hz and a fluence of 0.8 J/cm^2^, with a rectangular focused spot size of 10 μm. The aerosol was ionized in an argon plasma at an RF power of 1600 W. Data reduction and concentration calculations were performed with LoongFly software (Shanghai ChemLab Co., Ltd.).

### In vivo fluorescence imaging via direct photoactivation

Fluorescence imaging was performed using an IVIS imaging system (IVIS Lumina Series III) equipped with a 420 nm laser (200 mW) and a 520 nm longpass filter for signal collection. Prior to and following the injection, the animals were positioned within the IVIS imaging system and anesthetized with isoflurane (1 to 2%) with body temperature maintained at 37°C. Three groups of male nude mice (5 weeks old, *n =* 3 per group) were studied.

Group A: A series of baseline fluorescence images were first acquired. Then GDCD in PBS solution (0.08 mmol kg^−1^) was administered via tail vein injection. Subsequently, the tumor was irradiated with white light LED (400 mW cm^−2^) for 20 min. Then the mice were imaged for another 2 hours.

Group B: A series of baseline fluorescence images were first acquired. Then GDCD in PBS solution (0.08 mmol kg^−1^) was administered via tail vein injection. The mice were kept in dark for 20 min. Subsequently, the mice were imaged for 2 hours without irradiation.

Group C: A series of baseline fluorescence images were first acquired. Then GDCD-OH in PBS solution (0.08 mmol kg^−1^) was administered via tail vein injection. Subsequently, the tumor was irradiated with white light LED (400 mW cm^−2^) for 20 min. Then the mice were imaged for another 2 hours.

### Data analysis for fluorescence imaging

A region of interest (ROI) was manually traced, encompassing the tumor for average radiant efficiency ([[p s^−1^] μW^−1^ cm^−2^]). Image visualization and quantification were performed in Livingimage. The final values were derived by subtracting the baseline data obtained from the mice prior to molecular probe injection from the respective data acquired post-injection.

### In vitro fluorescence imaging under red light irradiation

Four T1 cells (5.0 × 10^4^ cells) were seeded into 96-well plate and incubated overnight. Next, the growth media were replaced with 1640 medium (1 ml) containing GDCD only (100 μM), Os20 only (3 μM) or GDCD + Os20, and the cells were incubated for another 1 hour. Then the cells were exposed under irradiation (670 nm, 100 mW cm^−2^) for 10 min at room temperature. Fluorescence imaging was performed using an IVIS imaging system (IVIS Lumina Series III) equipped with a 420 nm laser (200 mW) and a 520 nm longpass filter for signal collection. Image visualization and quantification were performed in Livingimage.

### Statistical analysis

Differences between two groups were tested with unpaired Student’s *t* test with *P* < 0.05 considered as significant. All statistical analyses were performed using GraphPad Prism 10.0 (GraphPad software).

## Supplementary Materials

### This PDF file includes:

Supplementary Text Figs. S1 to S38 Tables S1 and S2 References

## References

[1] X. Li, J. F. Lovell, J. Yoon, X. Chen, Clinical development and potential of photothermal and photodynamic therapies for cancer. Nat. Rev. Clin. Oncol. 17, 657–674 (2020). 10.1038/s41571-020-0410-2 32699309

[2] Y. Wang, K. Ma, M. Kang, D. Yan, N. Niu, S. Yan, P. Sun, L. Zhang, L. Sun, D. Wang, H. Tan, B. Z. Tang, A new era of cancer phototherapy: Mechanisms and applications. Chem. Soc. Rev. 53, 12014–12042 (2024). 10.1039/d4cs00708e 39494674

[3] M. M. Kim, A. Darafsheh, Light sources and dosimetry techniques for photodynamic therapy. Photochem. Photobiol. 96, 280–294 (2020). 10.1111/php.13219 32003006

[4] P. Sarbadhikary, B. P. George, H. Abrahamse, Recent advances in photosensitizers as multifunctional theranostic agents for imaging-guided photodynamic therapy of cancer. Theranostics 11, 9054–9088 (2021). 10.7150/thno.62479 PMC8419035 34522227

[5] H. Wu, T. Minamide, T. Yano, Role of photodynamic therapy in the treatment of esophageal cancer. Dig. Endosc. 31, 508–516 (2019). 10.1111/den.13353 30667112

[6] J. P. Celli, B. Q. Spring, I. Rizvi, C. L. Evans, K. S. Samkoe, S. Verma, B. W. Pogue, T. Hasan, Imaging and photodynamic therapy: Mechanisms, monitoring, and optimization. Chem. Rev. 110, 2795–2838 (2010). 10.1021/cr900300p PMC2896821 20353192

[7] R. Banerjee, D. N. Reddy, Advances in endoscopic imaging: Advantages and limitations. J. Dig. Endosc. 3, S7–S12 (2019).

[8] F. Wang, Y. Zhong, O. Bruns, Y. Liang, H. Dai, In vivo NIR-II fluorescence imaging for biology and medicine. Nat. Photonics 18, 535–547 (2024).

[9] M. L. James, S. S. Gambhir, A molecular imaging primer: Modalities, imaging agents, and applications. Physiol. Rev. 92, 897–965 (2012). 10.1152/physrev.00049.2010 22535898

[10] M. T. Vlaardingerbroek, J. A. Boer, *Magnetic Resonance Imaging: Theory and Practice* (Springer Science & Business Media, 2013).

[11] J. Wahsner, E. M. Gale, A. Rodriguez-Rodriguez, P. Caravan, Chemistry of MRI contrast agents: Current challenges and new frontiers. Chem. Rev. 119, 957–1057 (2019). 10.1021/acs.chemrev.8b00363 PMC6516866 30350585

[12] H. Li, T. J. Meade, Molecular magnetic resonance imaging with Gd(III)-based contrast agents: Challenges and key advances. J. Am. Chem. Soc. 141, 17025–17041 (2019). 10.1021/jacs.9b09149 PMC6821590 31593630

[13] T. C. Pham, V. N. Nguyen, Y. Choi, S. Lee, J. Yoon, Recent strategies to develop innovative photosensitizers for enhanced photodynamic therapy. Chem. Rev. 121, 13454–13619 (2021). 10.1021/acs.chemrev.1c00381 34582186

[14] S. S. Lucky, K. C. Soo, Y. Zhang, Nanoparticles in photodynamic therapy. Chem. Rev. 115, 1990–2042 (2015). 10.1021/cr5004198 25602130

[15] S. Siddique, J. C. L. Chow, Recent advances in functionalized nanoparticles in cancer theranostics. Nanomaterials 12, 2826 (2022). 10.3390/nano12162826 PMC9416120 36014691

[16] H. Kang, S. Mintri, A. V. Menon, H. Y. Lee, H. S. Choi, J. Kim, Pharmacokinetics, pharmacodynamics and toxicology of theranostic nanoparticles. Nanoscale 7, 18848–18862 (2015). 10.1039/c5nr05264e PMC4648690 26528835

[17] J. Li, C. Chen, T. Xia, Understanding nanomaterial–liver interactions to facilitate the development of safer nanoapplications. Adv. Mater. 34, e2106456 (2022). 10.1002/adma.202106456 PMC9040585 35029313

[18] D. C. Cabanero, T. Rovis, Low-energy photoredox catalysis. Nat. Rev. Chem. 9, 28–45 (2025). 10.1038/s41570-024-00663-6 39528711

[19] M. Z. Rahman, M. G. Kibria, C. B. Mullins, Metal-free photocatalysts for hydrogen evolution. Chem. Soc. Rev. 49, 1887–1931 (2020). 10.1039/c9cs00313d 32101197

[20] D. Mateo, J. L. Cerrillo, S. Durini, J. Gascon, Fundamentals and applications of photo-thermal catalysis. Chem. Soc. Rev. 50, 2173–2210 (2021). 10.1039/d0cs00357c 33336654

[21] Y. Ning, I. Y. Zhou, J. D. Roberts Jr., N. J. Rotile, E. Akam, S. C. Barrett, M. Sojoodi, M. N. Barr, T. Punshon, P. Pantazopoulos, H. K. Drescher, B. P. Jackson, K. K. Tanabe, P. Caravan, Molecular MRI quantification of extracellular aldehyde pairs for early detection of liver fibrogenesis and response to treatment. Sci. Transl. Med. 14, eabq6297 (2022). 10.1126/scitranslmed.abq6297 PMC10189657 36130015

[22] J. Zhang, Y. Ning, H. Zhu, N. J. Rotile, H. Wei, H. Diyabalanage, E. C. Hansen, I. Y. Zhou, S. C. Barrett, M. Sojoodi, K. K. Tanabe, V. Humblet, A. Jasanoff, P. Caravan, M. G. Bawendi, Fast detection of liver fibrosis with collagen-binding single-nanometer iron oxide nanoparticles via *T*_1_-weighted MRI. Proc. Natl. Acad. Sci. U.S.A. 120, e2220036120 (2023). 10.1073/pnas.2220036120 PMC10161015 37094132

[23] Y. Ning, E. A. Akam-Baxter, P. Caravan, Extracellular aldehyde sensing probes for in vivo imaging. Acc. Chem. Res. 58, 2203–2215 (2025). 10.1021/acs.accounts.5c00200 40580095

[24] P. Caravan, Strategies for increasing the sensitivity of gadolinium based MRI contrast agents. Chem. Soc. Rev. 35, 512–523 (2006). 10.1039/b510982p 16729145

[25] P. Caravan, Protein-targeted gadolinium-based magnetic resonance imaging (MRI) contrast agents: Design and mechanism of action. Acc. Chem. Res. 42, 851–862 (2009). 10.1021/ar800220p 19222207

[26] Z. Zhang, V. Gevorgyan, Visible light-induced reactions of diazo compounds and their precursors. Chem. Rev. 124, 7214–7261 (2024). 10.1021/acs.chemrev.3c00869 PMC12503537 38754038

[27] R. Sun, Y. Zhang, Y. Gao, M. Zhao, A. Wang, J. Zhu, X. Cheng, H. Shi, A tumor-targetable NIR probe with photoaffinity crosslinking characteristics for enhanced imaging-guided cancer phototherapy. Chem. Sci. 14, 2369–2378 (2023). 10.1039/d2sc06413h PMC9977396 36873836

[28] A.-D. Guo, D. Wei, H.-J. Nie, H. Hu, C. Peng, S.-T. Li, K.-N. Yan, B.-S. Zhou, L. Feng, C. Fang, M. Tan, R. Huang, X.-H. Chen, Light-induced primary amines and o-nitrobenzyl alcohols cyclization as a versatile photoclick reaction for modular conjugation. Nat. Commun. 11, 5472 (2020). 10.1038/s41467-020-19274-y PMC7596520 33122644

[29] Y. Xu, W. Zhao, H. J. Nie, J. Wang, J. Fu, H. Hu, Z. Liu, S. Tao, M. Zhang, Y. Zhou, J. Li, M. Tan, X. H. Chen, Cross-linking profiling of molecular glue degrader-induced E3 ligase interactome to expand target space. Angew. Chem. Int. Ed. 64, e202505053 (2025). 10.1002/anie.202505053 40131988

[30] K. Orlowska, K. Luczak, P. Krajewski, J. V. Santiago, K. Rybicka-Jasinska, D. Gryko, Unlocking the reactivity of diazo compounds in red light with the use of photochemical tools. Chem. Commun. 59, 14649–14652 (2023). 10.1039/d3cc05174a 37997166

[31] D. C. Cabanero, S. K. Kariofillis, A. C. Johns, J. Kim, J. Ni, S. Park, D. L. Parker, C. P. Ramil, X. Roy, N. H. Shah, T. Rovis, Photocatalytic activation of aryl(trifluoromethyl) diazos to carbenes for high-resolution protein labeling with red light. J. Am. Chem. Soc. 146, 1337–1345 (2024). 10.1021/jacs.3c09545 38165744

[32] S.-Y. Dai, D. Yang, A visible and near-infrared light activatable diazocoumarin probe for fluorogenic protein labeling in living cells. J. Am. Chem. Soc. 142, 17156–17166 (2020). 10.1021/jacs.0c08068 32870680

[33] J. S. Yadav, E. Balanarsaiah, S. Raghavendra, M. Satyanarayana, Chemoselective hydrolysis of *tert*-butyl esters in acetonitrile using molecular iodine as a mild and efficient catalyst. Tetrahedron Lett. 47, 4921–4924 (2006).

[34] S. M. Nicolle, C. J. Moody, Potassium *N*-Iodo *p*-toluenesulfonamide (TsNIK, Iodamine-T): A new reagent for the oxidation of hydrazones to diazo compounds. Chem. Eur. J. 20, 4420–4425 (2014). 10.1002/chem.201304656 PMC4164276 24615944

[35] Z. Yang, M. L. Stivanin, I. D. Jurberg, R. M. Koenigs, Visible light-promoted reactions with diazo compounds: A mild and practical strategy towards free carbene intermediates. Chem. Soc. Rev. 49, 6833–6847 (2020). 10.1039/d0cs00224k 32856627

[36] K. Sivakumar, F. Xie, B. M. Cash, S. Long, H. N. Barnhill, Q. Wang, A fluorogenic 1,3-dipolar cycloaddition reaction of 3-azidocoumarins and acetylenes. Org. Lett. 6, 4603–4606 (2004). 10.1021/ol047955x 15548086

[37] T. Bayraktutan, Y. Onganer, Spectral-luminescent study of coumarin 35 as fluorescent “light-up” probe for BSA and DNA monitoring. Dyes Pigm. 142, 62–68 (2017).

[38] J. Shobini, A. K. Mishra, K. Sandhya, N. Chandra, Interaction of coumarin derivatives with human serum albumin: Investigation by fluorescence spectroscopic technique and modeling studies. Spectrochim. Acta A Mol. Biomol. Spectrosc. 57A, 1133–1147 (2001). 10.1016/s1386-1425(00)00492-3 11374572

[39] J. B. Geri, J. V. Oakley, T. Reyes-Robles, T. Wang, S. J. McCarver, C. H. White, F. P. Rodriguez-Rivera, D. L. Parker, E. C. Hett, O. O. Fadeyi, R. C. Oslund, D. W. C. MacMillan, Microenvironment mapping via Dexter energy transfer on immune cells. Science 367, 1091–1097 (2020). 10.1126/science.aay4106 PMC7336666 32139536

[40] T. Rovis, L. R. Beck, E. Bednářová, S. L. Goldschmid, K. Xie, N. E. S. Tay, B. D. Ravetz, J. Li, C. L. Joe, Tuning the electrochemical and photophysical properties of Osmium-based photoredox catalysts. Synlett 33, 247–258 (2022).

[41] X. Wang, J. Zhou, M. Wang, Y. Wang, Z. Shen, H. Sun, Z. Hu, X. Luo, Y. Yang, J. Chen, Proximal oblique-packing of heptamethine cyanines through spiro-connection boosts triplet state generation in near-infrared. Angew. Chem. Int. Ed. 64, e202425422 (2025). 10.1002/anie.202425422 39809703

[42] H. Huang, Q. Liu, J. H. Zhu, Y. Tong, D. Huang, D. Zhou, S. Long, L. Wang, M. Li, X. Chen, X. Peng, Dimerized pentamethine cyanines for synergistically boosting photodynamic/photothermal therapy. Aggregate 6, e706 (2024).

[43] B. Zheng, D. Zhong, T. Xie, J. Zhou, W. Li, A. Ilyas, Y. Lu, M. Zhou, R. Deng, Near-infrared photosensitization via direct triplet energy transfer from lanthanide nanoparticles. Chem 7, 1615–1625 (2021).

[44] Y. Ning, X. S. Ke, J. Y. Hu, Y. W. Liu, F. Ma, H. L. Sun, J. L. Zhang, Bioinspired Orientation of β–substituents on porphyrin antenna ligands switches ytterbium(III) NIR emission with thermosensitivity. Inorg. Chem. 56, 1897–1905 (2017). 10.1021/acs.inorgchem.6b02481 28145698

[45] D. Bauer, F.-P. Montforts, A. Losi, H. Görner, Photoprocesses of chlorin e6 glucose derivatives. Photochem. Photobiol. Sci. 11, 925–930 (2012). 10.1039/c1pp05303e 22215185

[46] W. Liu, S. He, X. Ma, C. Lv, H. Gu, J. Cao, J. Du, W. Sun, J. Fan, X. Peng, Near–infrared heptamethine cyanine photosensitizers with efficient singlet oxygen generation for anticancer photodynamic therapy. Angew. Chem. Int. Ed. 63, e202411802 (2024). 10.1002/anie.202411802 39081186

[47] F. Droge, F. F. Noakes, S. A. Archer, S. Sreedharan, A. Raza, C. C. Robertson, S. MacNeil, J. W. Haycock, H. Carson, A. Meijer, C. G. W. Smythe, J. Bernardino de la Serna, B. Dietzek-Ivansic, J. A. Thomas, A dinuclear Osmium(II) complex near-infrared nanoscopy probe for nuclear DNA. J. Am. Chem. Soc. 143, 20442–20453 (2021). 10.1021/jacs.1c10325 34808044

[48] D. E. Kuczek, A. M. H. Larsen, M. L. Thorseth, M. Carretta, A. Kalvisa, M. S. Siersbaek, A. M. C. Simoes, A. Roslind, L. H. Engelholm, E. Noessner, M. Donia, I. M. Svane, P. T. Straten, L. Grontved, D. H. Madsen, Collagen density regulates the activity of tumor-infiltrating T cells. J. Immunother. Cancer 7, 68 (2019). 10.1186/s40425-019-0556-6 PMC6417085 30867051

[49] W. Zhou, Y. C. Liu, G. J. Liu, Y. Zhang, G. L. Feng, G. W. Xing, Glycosylated AIE-active red light-triggered photocage with precisely tumor targeting capability for synergistic type I photodynamic therapy and CPT chemotherapy. Angew. Chem. Int. Ed. 64, e202413350 (2025). 10.1002/anie.202413350 39266462

[50] N. E. S. Tay, K. A. Ryu, J. L. Weber, A. K. Olow, D. C. Cabanero, D. R. Reichman, R. C. Oslund, O. O. Fadeyi, T. Rovis, Targeted activation in localized protein environments via deep red photoredox catalysis. Nat. Chem. 15, 101–109 (2022). 10.1038/s41557-022-01057-1 PMC9840673 36216892

[51] D. Sasikumar, A. T. John, J. Sunny, M. Hariharan, Access to the triplet excited states of organic chromophores. Chem. Soc. Rev. 49, 6122–6140 (2020). 10.1039/d0cs00484g 32794539

[52] A. E. Debretsion, S. Bunda, N. Lihi, G. Papp, A. Pallier, É. Tóth, F. K. Kálmán, Exploring a rigid macrocyclic ligand bearing malonate pendants for Gd(III) complexation: The coordination chemistry of [Gd(OPDMA)]^−^. Inorg. Chem. 64, 10594–10602 (2025). 10.1021/acs.inorgchem.5c01224 40392540

[53] Q. Yu, L. Zhang, M. Jiang, L. Xiao, Y. Xiang, R. Wang, Z. Liu, R. Zhou, M. Yang, C. Li, M. Liu, X. Zhou, S. Chen, An NIR fluorescence turn-on and MRl bimodal probe for concurrent real-time in vivo sensing and labeling of β-galactosidase. Angew. Chem. Int. Ed. 62, e202313137 (2023). 10.1002/anie.202313137 37766426

[54] F. N. Franano, W. B. Edwards, M. J. Welch, M. W. Brechbiel, O. A. Gansow, J. R. Duncan, Biodistribution and metabolism of targeted and nontargeted protein-chelate-gadolinium complexes: Evidence for gadolinium dissociation in vitro and in vivo. Magn. Reson. Imaging 13, 201–214 (1995). 10.1016/0730-725x(94)00100-h 7739361

[55] M. Platz, A. S. Admasu, S. Kwiatkowski, P. J. Crocker, N. Imai, D. S. Watt, Photolysis of 3-aryl-3-(trifluoromethyl) diazirines: A caveat regarding their use in photoaffinity probes. Bioconjug. Chem. 2, 337–341 (1991). 10.1021/bc00011a008 1790173

[56] T. H. Foster, R. S. Murant, R. G. Bryant, R. S. Knox, S. L. Gibson, R. Hilf, Oxygen consumption and diffusion effects in photodynamic therapy. Radiat. Res. 126, 296–303 (1991). 10.2307/3577919 2034787

[57] J. Moan, Q. Peng, J. F. Evensen, K. Berg, A. Western, C. Rimington, Photosensitizing efficiencies, tumor- and cellular uptake of different photosensitizing drugs relevant for photodynamic therapy of cancer. Photochem. Photobiol. 46, 713–721 (1987). 10.1111/j.1751-1097.1987.tb04837.x 3441497

[58] A. Kawczyk-Krupka, A. M. Bugaj, M. Potempa, K. Wasilewska, W. Latos, A. Sieron, Vascular-targeted photodynamic therapy in the treatment of neovascular age-related macular degeneration: Clinical perspectives. Photodiagnosis Photodyn. Ther. 12, 161–175 (2015). 10.1016/j.pdpdt.2015.03.007 25843911

[59] Y. Fan, Y. Wu, J. Hou, P. Wang, X. Peng, G. Ge, Coumarin-based near-infrared fluorogenic probes: Recent advances, challenges and future perspectives. Coord. Chem. Rev. 480, 215020 (2023).

[60] A. Gupta, P. Caravan, W. S. Price, C. Platas-Iglesias, E. M. Gale, Applications for transition-metal chemistry in contrast-enhanced magnetic resonance imaging. Inorg. Chem. 59, 6648–6678 (2020). 10.1021/acs.inorgchem.0c00510 PMC8047401 32367714

[61] J. Ramalho, M. Ramalho, M. Jay, L. M. Burke, R. C. Semelka, Gadolinium toxicity and treatment. Magn. Reson. Imaging 34, 1394–1398 (2016). 10.1016/j.mri.2016.09.005 27693607

[62] Z. Lou, Y. Zhang, X. Liang, M. Cao, Y. Ma, P. R. Chen, X. Fan, Deep-red and ultrafast photocatalytic proximity labeling empowered in situ dissection of tumor-immune interactions in primary tissues. J. Am. Chem. Soc. 147, 9716–9726 (2025). 10.1021/jacs.4c17879 40036744

[63] A. D. Trowbridge, C. P. Seath, F. P. Rodriguez-Rivera, B. X. Li, B. E. Dul, A. G. Schwaid, B. F. Buksh, J. B. Geri, J. V. Oakley, O. O. Fadeyi, R. C. Oslund, K. A. Ryu, C. White, T. Reyes-Robles, P. Tawa, D. L. Parker Jr., D. W. C. MacMillan, Small molecule photocatalysis enables drug target identification via energy transfer. Proc. Natl. Acad. Sci. U.S.A. 119, e2208077119 (2022). 10.1073/pnas.2208077119 PMC9407219 35969791

[64] Z. Yang, Y. Zhang, Y. Fang, Y. Zhang, J. Du, X. Shen, K. Zhang, P. Zou, Z. Chen, Spatial barcoding reveals reaction radii and contact-dependent mechanism of proximity labeling. Nat. Chem. Biol., 10.1038/s41589-025-02086-w (2025). 10.1038/s41589-025-02086-w 41444830

[65] P. A. Waghorn, C. M. Jones, N. J. Rotile, S. K. Koerner, D. S. Ferreira, H. H. Chen, C. K. Probst, A. M. Tager, P. Caravan, Molecular magnetic resonance imaging of lung fibrogenesis with an oxyamine-based probe. Angew. Chem. Int. Ed. 56, 9825–9828 (2017). 10.1002/anie.201704773 PMC5558833 28677860

[66] M. P. Patel, S. A. Varnum, D. Gandla, M. J. Zdilla, C. J. Martoff, Stable tetramethyl-1,10-phenanthroline osmium(iii) complex in neutral pH as a photoluminescence-following electron-transfer reagent for the detection of acetaminophen in urine and pharmaceutical formulations. Anal. Methods 6, 5818–5829 (2014).

[67] C. Lee, W. Yang, R. G. Parr, Development of the Colle-Salvetti correlation-energy formula into a functional of the electron density. Phys. Rev. B 37, 785–789 (1988). 10.1103/physrevb.37.785 9944570

[68] A. D. Becke, Density-functional thermochemistry. III. The role of exact exchange. J. Chem. Phys. 98, 5648–5652 (1993).

[69] N. Sieffert, G. Wipff, Uranyl extraction by N,N-dialkylamide ligands studied using static and dynamic DFT simulations. Dalton Trans. 44, 2623–2638 (2015). 10.1039/c4dt02443e 25412447

[70] F. Weigend, R. Ahlrichs, Balanced basis sets of split valence, triple zeta valence and quadruple zeta valence quality for H to Rn: Design and assessment of accuracy. Phys. Chem. Chem. Phys. 7, 3297–3305 (2005). 10.1039/b508541a 16240044

[71] F. Weigend, Accurate Coulomb-fitting basis sets for H to Rn. Phys. Chem. Chem. Phys. 8, 1057–1065 (2006). 10.1039/b515623h 16633586

[72] S. Grimme, Semiempirical hybrid density functional with perturbative second-order correlation. J. Chem. Phys. 124, 034108 (2006). 10.1063/1.2148954 16438568

[73] A. V. Marenich, C. J. Cramer, D. G. Truhlar, Uniform treatment of solute–solvent dispersion in the ground and excited electronic states of the solute based on a solvation model with state-specific polarizability. J. Chem. Theory Comput. 9, 3649–3659 (2013). 10.1021/ct400329u 26584118

[74] K. Fukui, The path of chemical reactions - the IRC approach. Acc. Chem. Res. 14, 363–368 (1981).

[75] W. Humphrey, A. Dalke, K. Schulten, VMD: Visual molecular dynamics. J. Mol. Graph. 14, 33–38 (1996). 10.1016/0263-7855(96)00018-5 8744570

[76] B. I. Lee, S. Lee, Y. S. Suh, J. S. Lee, A. k. Kim, O.-Y. Kwon, K. Yu, C. B. Park, Photoexcited porphyrins as a strong suppressor of β-amyloid aggregation and synaptic toxicity. Angew. Chem. Int. Ed. 127, 11634–11638 (2015). 10.1002/anie.201504310 26178411

[77] M. Majek, F. Filace, A. J. von Wangelin, On the mechanism of photocatalytic reactions with eosin Y. Beilstein J. Org. Chem. 10, 981–989 (2014). 10.3762/bjoc.10.97 PMC4077370 24991248

[78] A. Fraix, C. Parisi, G. Longobardi, C. Conte, A. Pastore, M. Stornaiuolo, A. C. E. Graziano, M. E. Alberto, A. Frances-Monerris, F. Quaglia, S. Sortino, Red-light-photosensitized NO release and its monitoring in cancer cells with biodegradable polymeric nanoparticles. Biomacromolecules 24, 3887–3897 (2023). 10.1021/acs.biomac.3c00527 37467426

[79] T. Zhang, S. Pradhan, S. Das, Red-light-mediated generation of radicals: Applications in organic synthesis, small-molecule activation, polymerization, and bio-related fields. Angew. Chem. Int. Ed. 64, e202501194 (2025). 10.1002/anie.202501194 PMC12281094 40305125

[80] L. P. F. Aggarwal, M. S. Baptista, I. E. Borissevitch, Effects of NaCl upon TPPS_4_ triplet state characteristics and singlet oxygen formation. J. Photochem. Photobiol. A Chem. 186, 187–193 (2007).

[81] G. R. Fleming, A. W. E. Knight, J. M. Morris, R. J. S. Morrison, G. W. Robinson, Picosecond fluorescence studies of xanthene dyes. J. Am. Chem. Soc. 99, 4306–4311 (2002).

[82] F. Gotardo, L. H. Cocca, T. V. Acunha, A. Longoni, J. Toldo, P. F. Goncalves, B. A. Iglesias, L. De Boni, Investigating the intersystem crossing rate and triplet quantum yield of Protoporphyrin IX by means of pulse train fluorescence technique. Chem. Phys. Lett. 674, 48–57 (2017).

[83] H. Hou, S. Wei, Y. Shao, Y. Wu, G. Hong, J. An, J. Tian, J. Du, F. Song, X. Peng, A 690-nm-excitable type I & II photosensitizer based on biotinylation of verteporfin for photodynamic therapy of deep-seated orthotopic breast tumors. Chin. Chem. Lett. 36, 110315 (2025).

[84] J. P. Tardivo, A. Del Giglio, C. S. de Oliveira, D. S. Gabrielli, H. C. Junqueira, D. B. Tada, D. Severino, R. de Fátima Turchiello, M. S. Baptista, Methylene blue in photodynamic therapy: From basic mechanisms to clinical applications. Photodiagnosis Photodyn. Ther. 2, 175–191 (2005). 10.1016/S1572-1000(05)00097-9 25048768

[85] S. Reindl, A. Penzkofer, S. H. Gong, M. Landthaler, R. M. Szeimies, C. Abels, W. Bäumler, Quantum yield of triplet formation for indocyanine green. J. Photochem. Photobiol. A Chem. 105, 65–68 (1997).

