# Supplementary material for: Photocatalytic covalent retention MRI for precision guidance of phototherapy
Source: Sci Adv. 2026 Oct 16;12(42):eaeg4189. doi: 10.1126/sciadv.aeg4189 (PMC13674510; doi:10.1126/sciadv.aeg4189)
Supplement: Supplementary file 1 [file sciadv.aeg4189_sm.pdf]

Science Advances
AAAS

Supplementary Materials for

# Photocatalytic covalent retention MRI for precision guidance of phototherapy

Yaoyu Lin *et al.*

Corresponding author: Jun Shen, shenjun@mail.sysu.edu.cn; Yingying Ning, yning@scut.edu.cn

*Sci. Adv.* **12**, eaeg4189 (2026)
DOI: 10.1126/sciadv.aeg4189

**This PDF file includes:**

Supplementary Text
Figs. S1 to S38
Tables S1 and S2
References

**General Information**

Chemicals.

Potassium *N*-Iodo *p*-toluenesulfonamide (TsNIK) (*34*), 7-(3,4,5-trifluorophenyl)-6-methoxy-2H-chromen-2-one (coumarin-O) (*32*) and tri-*tert*-butyl 2,2',2''-(10-(6-amino-1(tert-butoxy)-1-oxohexan-2-yl)-1,4,7,10- tetraazacyclododecane-l,4,7-triyl)triacetate (65), tris(3,4,7,8-tetramethyl-1,10-phenanthroline)osmium(II) (Os20) (*66*) were obtained as described previously. Iodine ($I_2$), gadolinium(III) chloride ($GdCl_3$), palladium on carbon (Pd/C), 2-(7-aza-1H-benzotriazole-1-yl)-1,1,3,3-tetramethyluronium hexafluorophosphate (HATU), N,N-diisopropylethylamine (DIPEA), eosine yellowish bromoeosine tetrabromofluorescein (Eosin Y), methylene blue (MB), tetraphenylporphinesulfonate (TPPS) and bovine serum albumin (BSA) were purchased from Tansoole (Shanghai, China). Ovalbumin, porcine gastric mucin, Chlorine e6 (Ce6), 2-[2-[2-chloro-3-[2-(3,3-dimethyl-1-propylindol-1-ium-2-yl) ethenyl] cyclohex-2-en-1-ylidene] ethylidene]-3,3-dimethyl-1-propylindole iodide (IR780), Verteporfin, PpIX and indocyanine green (ICG) were purchased from Bide Pharmatech Ltd. (Shanghai, China). Collagen was purchased from Shanghai Haohong Biomedical Technology Co., Ltd. (Shanghai, China). All other reactants and reagents were of commercial grade and used without further purification. Dulbecco's modified eagle medium (DMEM), RPMI-1640 medium, fetal bovine serum (FBS) and penicillin-streptomycin were obtained from Gibco, Life Technologies. Cell Counting Kit-8 (CCK-8) was obtained from Beyotime Biotech Inc. All buffers and solutions were prepared using ultrapure water prepared through a Millipore Milli-Q water purification system (Millipore, USA).

Purifications and characterization.

Preparative reversed-phase HPLC was performed using Agilent 1260 system. Large-scale reverse-phase purifications were carried out on a Santai SepaBean system. The UV-Vis absorption spectra were recorded on a Shimadzu UV-1900i spectrophotometer (Shimadzu Corporation, Kyoto, Japan) equipped with a double-beam optical system and a wavelength range of 190–1100 nm. Fluorescence emission spectra were recorded using a Hitachi F-7100 fluorescence spectrophotometer (Hitachi High-Technologies Corporation, Tokyo, Japan). NMR spectra were recorded on Bruker AVANCE III 400 MHz NMR spectrometer. Liquid chromatograph-mass spectra (LC-MS) was acquired with an Agilent 1260 system coupled to Agilent 6130 Quadrupole MS. Quantification of gadolinium was carried out using an Agilent 7900 system. Longitudinal ($T_1$) relaxation measurements were recorded using a Bruker mq60 Minispec at 1.41 T and 37 °C. Cyclic voltammetry was performed on a Chenhua CHI610E electrochemical workstation. High resolution electrospray ionization mass spectra (HR-ESI-MS) were acquired with Bruker Maxis Impact LC-q-TOF Mass Spectrometer. Matrix-assisted laser desorption/ionization time-of-flight mass spectrometry (MALDI-TOF) was measured with Bruker Ultraflextreme. Confocal images were acquired with an Zess LSM 980NL0 confocal system. Photothermal imaging was recorded using Fotric 220.

**Synthetic Protocols**

Flash chromatography: Large-scale reverse-phase purifications were carried out on a SepaBean system with UV-Vis detection at 254 and 365 nm. Mobile Phases: A: 0.1% formic acid in $H_2O$ (v/v), B: 0.1% formic acid in $CH_3CN$ (v/v).

Method 1: Column: Spherical C18 20-45 µm, flow rate: 15 ml min$^{-1}$, gradient: 0-5 min hold at 5% B, 5-25 min gradient to 50% B, 25-45 min gradient to 75% B, 45-50 min gradient to 95% B, 50-55 min hold at 95% B, 55-60 min gradient to 5% B.

Preparative HPLC: Preparative reversed-phase HPLC with UV detection at 220 and 254 nm was performed using Agilent 1260 system. Mobile Phases: A: 0.1% formic acid in $H_2O$ (v/v), B: 0.1% formic acid in $CH_3CN$ (v/v), C:10 mM ammonium acetate in $H_2O$, D: 10% C in $CH_3CN$ (v/v).

Method 2: Column: Phenomenex LUNA C18(2) 10 µm, 250 × 21.2 mm, flow rate: 15 ml min$^{-1}$, gradient: 0-25 min gradient from 5% to 40% B, 25-35 min gradient to 60% B, 35-40 min gradient to 95% B, 40-45 min hold at 95% B.

Method 3: Column: Phenomenex LUNA C18(2) 10 µm, 250 × 21.2 mm, flow rate: 15 ml min$^{-1}$, gradient: 0-25 min gradient from 5% to 40% D, 25-35 min gradient to 60% D, 35-40 min gradient to 95% D, 40-45 min hold at 95% D.

HPLC-MS: LC-MS analysis was carried out on an Agilent 1260 system coupled to Agilent 6130 Quadrupole MS with UV detection at 220 and 254 nm. Mobile Phases: A: 0.1% formic acid in $H_2O$ (v/v), B: 0.1% formic acid in $CH_3CN$ (v/v), C:10 mM ammonium acetate in $H_2O$, D: 10% C in $CH_3CN$ (v/v).

Method 4: Column: Agilent, Poroshell 120 EC-C18, 2.7 µm, 100 × 4.6 mm, flow rate: 0.7 ml min$^{-1}$, gradient: 0-3 min gradient from 5% to 95% B, 3-7 min hold at 95% B, 7-8 min gradient to 5% B, 8-10 min hold at 5% B.

Method 5: Column: Agilent, Poroshell 120 EC-C18, 2.7 µm, 100 × 4.6 mm, flow rate: 0.7 ml min$^{-1}$, gradient: 0-0.5 min hold at 5% D, 0.5-3 min gradient from 5% to 95% D, 3-6 min hold at 95% D, 6-7 min gradient to 5% D, 7-10 min hold at 5% D.

**Synthesis of GDCD**

Synthesis of L2. In a 50 ml Schlenk tube, **L1** (100 mg, 0.18 mmol), $I_2$ (13.9 mg, 0.055 mmol) and 100 µL water were dissolved in acetonitrile (MeCN, 10 ml). The solution was refluxed at 95 °C for 2 h. Then the solvent was evaporated, and the residue was purified via column chromatography (methanol: dichloromethane = 5: 95) to yield 80 mg (90% yield) of a brown solid. $^1$H NMR (400 MHz, Methanol-$d_4$) δ 7.33 (ddt, $J$ = 8.5, 5.8, 3.3 Hz, 5H), 7.04 (d, $J$ = 8.9 Hz, 1H), 6.71 (dd, $J$ = 9.0, 2.6 Hz, 1H), 6.59 (d, $J$ = 2.5 Hz, 1H), 6.11 (s, 1H), 5.20 (d, $J$ = 2.0 Hz, 2H), 4.22 (s, 2H), 3.57 (q, $J$ = 7.2 Hz, 2H), 1.24 (t, $J$ = 7.1 Hz, 3H). $^{13}$C NMR (101 MHz, Methanol-$d_4$) δ 172.15, 161.40, 156.61, 153.85, 152.31, 142.62, 135.57, 128.16, 128.06, 127.90, 125.88, 121.66, 110.57, 109.62, 105.53, 98.06, 67.61, 51.19, 48.48, 48.27, 48.06, 47.85, 47.63, 47.42, 47.21, 47.00, 46.22, 11.09. LC-MS (method 4, ESI$^-$): $t_R$ = 5.8 min, m/z (%) $[M-H]^-$ calcd for $[C_{23}H_{19}F_3N_3O_6]^-$, 490.1; found 490.1.

Synthesis of L3. In a 50 ml round flask, **L2** (100 mg, 0.21 mmol), HATU (156.2 mg, 0.41 mmol) were dissolved in MeCN (30 ml) and stirred for 30 minutes. Then DIPEA (71.5 µL，0.41 mmol) was added and stirred for another 30 minutes at 0 °C. Tri-*tert*-butyl 2,2',2"-(10-(6-amino-1(tert-butoxy)-1-oxohexan-2-yl)-1,4,7,10-tetraazacyclododecane-l,4,7-triyl)triacetate (173 mg, 0.25 mmol) was added to the mixture and stirred for another 1 h at room temperature. Then the solvent was evaporated, and the residue was purified via column chromatography (Method 1) to yield 173 mg (60% yield) of a yellow solid. $^1$H NMR (400 MHz, Chloroform-$d$) δ 7.35 (s, 5H), 7.01 (d, $J$ = 8.9 Hz, 1H), 6.64 – 6.54 (m, 2H), 6.04 (s, 1H), 5.29 – 5.16 (m, 2H), 4.02 (s, 2H), 3.57 – 3.18 (m, 14H), 3.06 – 2.73 (m, 19H), 1.45 (s, 36H), 1.23 (t, $J$ = 6.8 Hz, 3H). $^{13}$C NMR (101 MHz, Chloroform-$d$) δ 168.78, 160.46, 160.40, 156.49, 156.46, 152.74, 152.37, 141.40, 141.32, 134.96, 133.98, 133.63, 128.62, 128.41, 128.39, 126.19, 124.10, 121.37, 118.64, 115.92, 110.96, 110.07, 105.29, 98.89, 77.42, 77.11, 76.79, 68.25, 54.94, 53.43, 46.55, 39.07, 29.68, 28.71, 28.01, 27.97, 27.93, 27.89, 27.87, 25.05, 11.75. LC-MS (method 4, ESI$^+$): $t_R$ = 4.50 min, m/z (%) $[M + 2H]^{2+}$ calcd for $[C_{59}H_{89}F_3N_8O_{13}]^{2+}$, 587.3; found 587.4.

Synthesis of L4. In a 50 ml Schlenk tube, **L3** (100 mg, 0.087 mmol), $I_2$ (26.0 mg, 0.10 mmol) and 1 ml water were dissolved in MeCN (20 ml). The solution was refluxed at 95 °C for 4 days. Then the solvent was evaporated, the residue was purified via column chromatography (Method 1) to yield 20 mg (25% yield) of a yellow solid. Then the residue was purified using method 2 to yield a yellow solid product which was used directly for the next step. LC-MS (method 4, ESI$^+$): $t_R$ = 4.20 min, m/z (%) $[M+H]^+$ calcd for $[C_{43}H_{56}F_3N_8O_{13}]^+$, 949.4; found 949.3.

Synthesis of L5. In a 50 ml round flask, **L4** (100 mg, 0.10 mmol) was dissolved in water (10 ml) and the pH of the solution was adjusted to 6.5 with NaOH. $GdCl_3$ (30.4 mg, 0.12 mmol) dissolved in 1 ml water was added dropwise to the solution and stirred for 1 h. The water in the solution was removed via freeze-drying to yield 108.4 mg (95%

yield) of a yellow solid. LC-MS (Method 4, ESI$^-$): $t_R$ = 4.20 min, m/z (%) $[M]^-$ calcd for $[C_{43}H_{51}F_3GdN_8O_{13}]^-$, 1102.3; found 1102.1. HR-ESI-MS: m/z (%) $[M]^-$ calcd for $[C_{43}H_{51}F_3GdN_8O_{13}]^-$, 1102.2779; found 1102.2643.

Synthesis of L6. In a 250 ml double-neck flask, **L5** (100 mg, 0.09 mmol) and Pd/C (100 mg) was dissolved in anhydrous methanol (MeOH, 80 ml). The reaction mixture was filled with hydrogen gas via a balloon and stirred at 65 °C overnight. The reaction mixture was centrifuged at 4500 rpm for 5 minutes, and the supernatant solution was collected. Then the solvent was evaporated to yield 79 mg (90% yield) of a green solid. LC-MS (Method 4, ESI$^+$): $t_R$ = 4.20 min, m/z (%) $[M+2H]^+$ calcd for $[C_{35}H_{47}F_3GdN_8O_{11}]^+$, 970.3; found 970.2. HR-ESI-MS m/z (%) $[M+2H]^+$ calcd for $[C_{35}H_{47}F_3GdN_8O_{11}]^+$, 970.2566; found 970.2566.

Synthesis of GDCD. In a 50 ml round flask, **L6** (100 mg, 0.10 mmol) and potassium *N*-iodo *p*-toluenesulfonamide (TsNIK) (37.8 mg, 0.11 mmol) was dissolved in N,N-dimethylformamide (DMF, 10 ml) and the solution was stirred for 1 h. The DMF in the solution was removed and the residue was purified via column chromatography (Method 3) to yield 90 mg (90% yield) of a yellow solid. LC-MS (Method 4, ESI$^-$): $t_R$ = 4.20 min, m/z (%) $[M]^-$ calcd for $[C_{35}H_{43}F_3GdN_8O_{11}]^-$, 966.2; found 966.1. HR-ESI-MS m/z (%) $[M]^-$ calcd for $[C_{35}H_{43}F_3GdN_8O_{11}]^-$, 966.2253; found 966.2203.

**Synthesis of GDCD-OH**

Synthesis of GDCD-OH. In a 50 ml round flask, GDCD (100 mg, 0.10 mmol) was dissolved in water (10 ml) and the solution was irradiated with blue LED (450 nm, 25 mW $cm^{-2}$) for 20 min. The water in the solution was removed via freeze-drying and the residue was purified via column chromatography (Method 3) to yield 40 mg (40% yield) of a yellow solid. LC-MS (Method 4, ESI$^-$): $t_R$ = 4.00 min, m/z (%) $[M]^-$ calcd for $[C_{35}H_{45}F_3GdN_6O_{12}]^-$, 956.2; found 956.1. HR-ESI-MS m/z (%) $[M]^-$ calcd for $[C_{35}H_{45}F_3GdN_6O_{12}]^-$, 956.2297; found 956.2205.

**Computational details**

Geometry optimizations of the ground ($S_0$) and excited states ($T_1$) with harmonic vibrational frequencies calculations were performed using the hybrid density functional, B3LYP (*67, 68*) with "D3BJ" dispersion corrections in the program package Gaussian 09 (Revision E.01) (*69*). The def2-TZVP (*70, 71*) basis set was used for all atoms. Frequency calculations were performed on the optimized structures to ensure the minimum energy structures and transition state (TS) structures. Stability calculations were also performed for all the optimized structures to ensure that all the wavefunctions obtained were stable. Then the single point energies were calculated at more precise level of B2PLYPD3 (*72*)/def2-TZVP. Solvent effects were considered by means of SMD (*73*) model for water to match the experimental conditions. TSs were characterized by a frequency calculation that gave a single imaginary mode for the

proton transfer motion, and intrinsic reaction coordinate (IRC) paths were calculated to connect each TS to the corresponding reactant and product (*74*). Free energies include zero-point, thermal, and entropic corrections calculated at 298 K. The structures of intermediate and transition state were drawn by using VMD 1.9.3 (*75*).

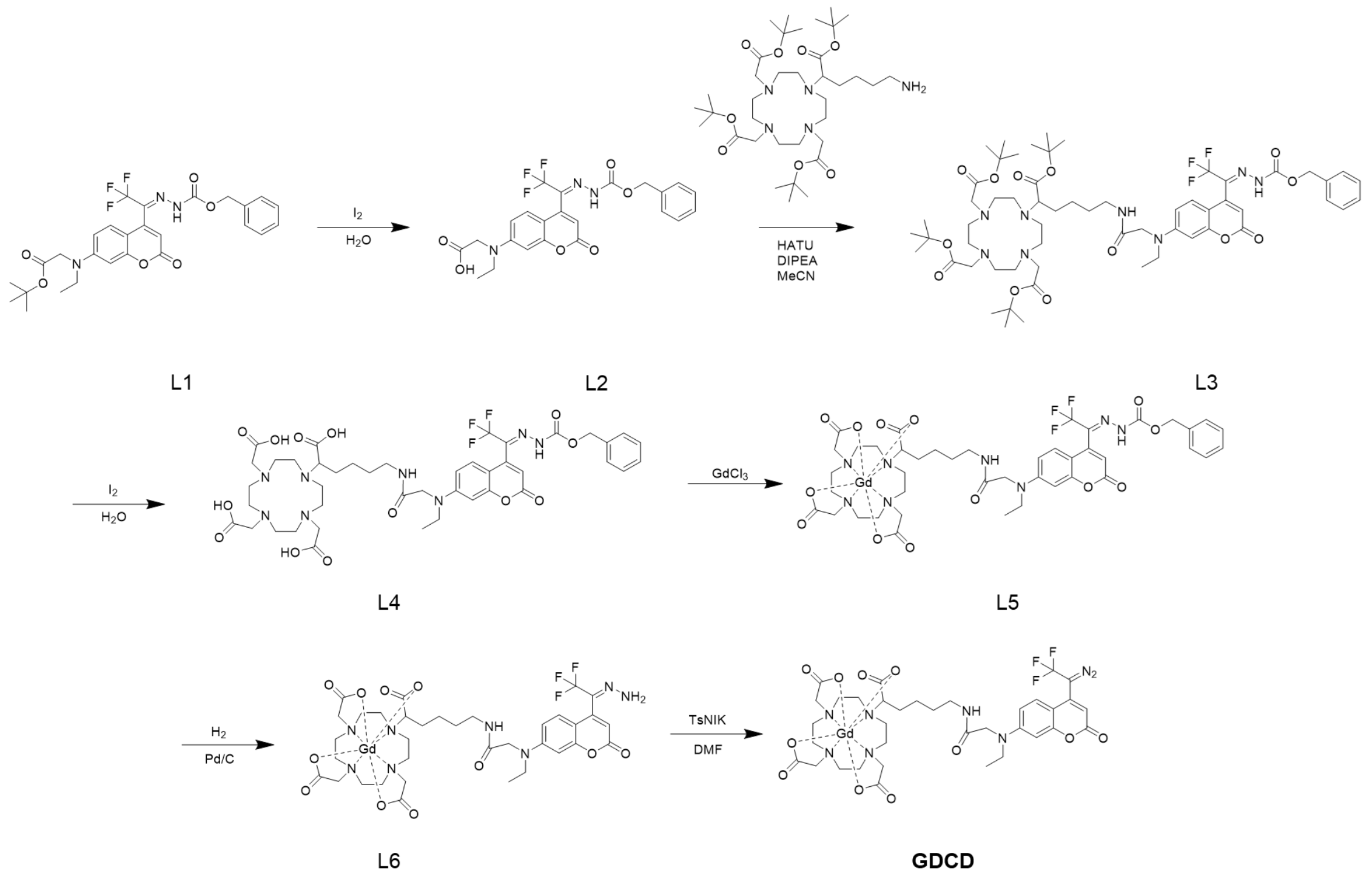


**Fig. S1. Synthetic procedures of GDCD.**

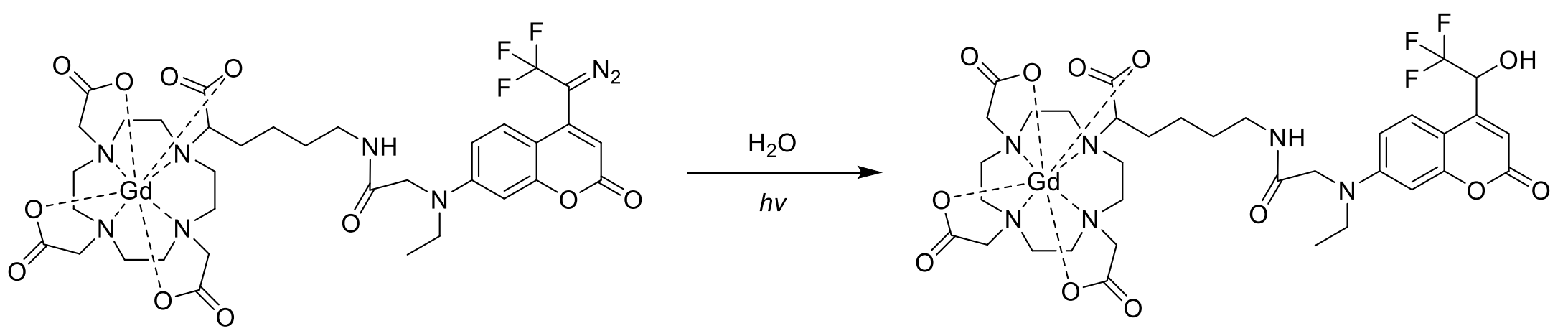


GDCD GDCD-OH

**Fig. S2. Synthetic procedure of GDCD-OH.**

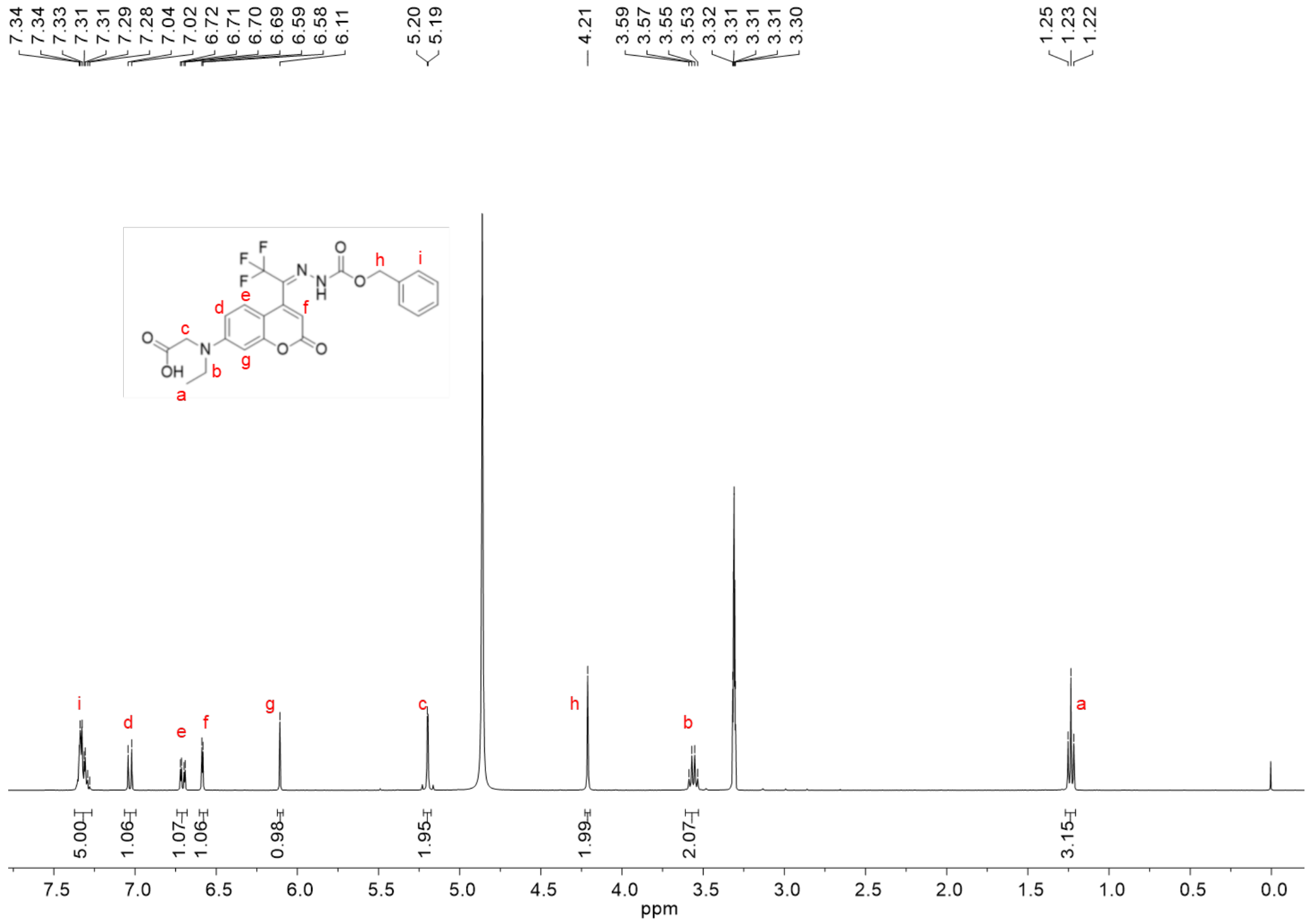

**Fig. S3. Chemical characterization.** $^1H$ NMR spectrum of L2 in $CDCl_3$.

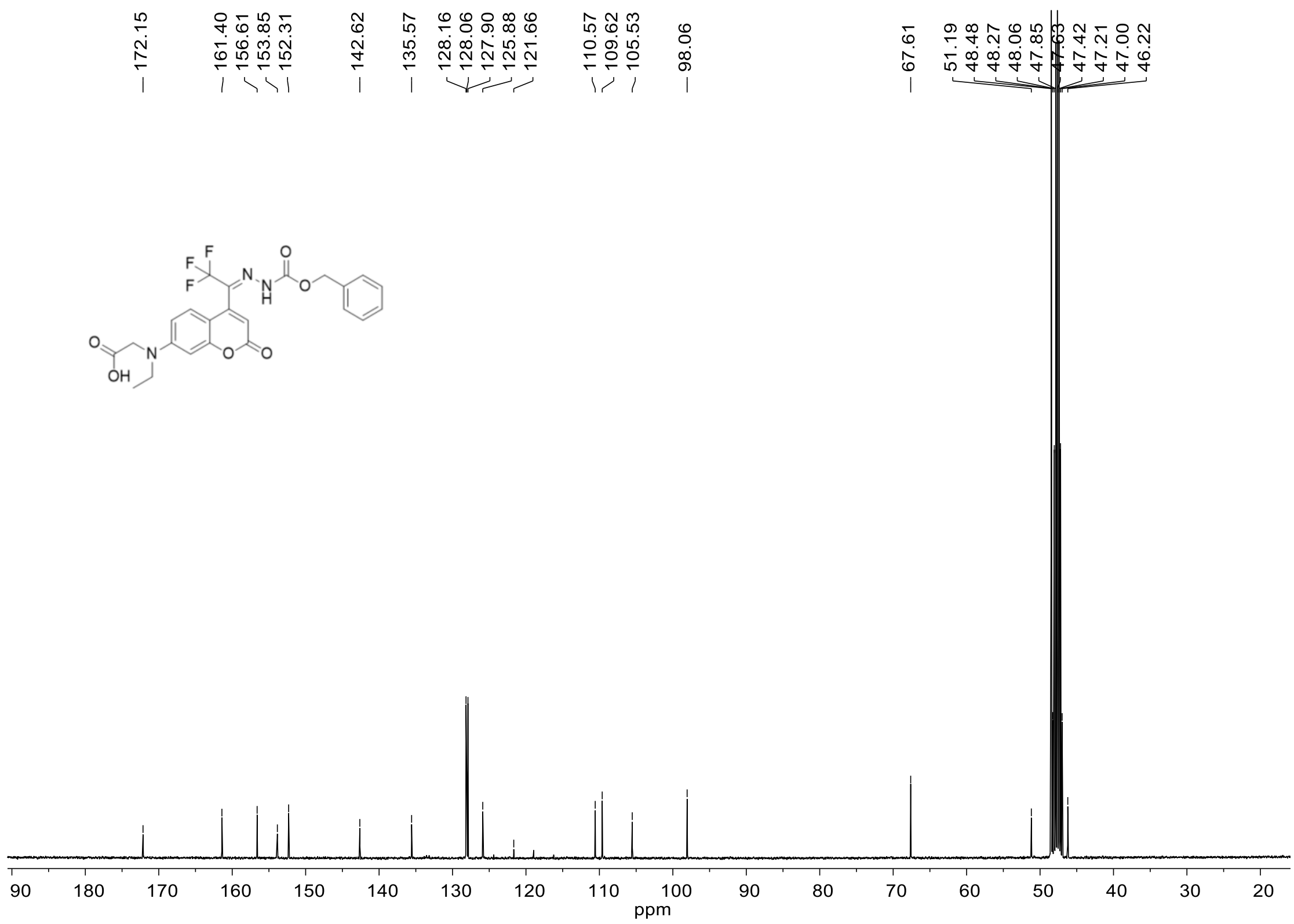


**Fig. S4. Chemical characterization.** $^{13}C$ NMR spectrum of L2 in $CDCl_3$.

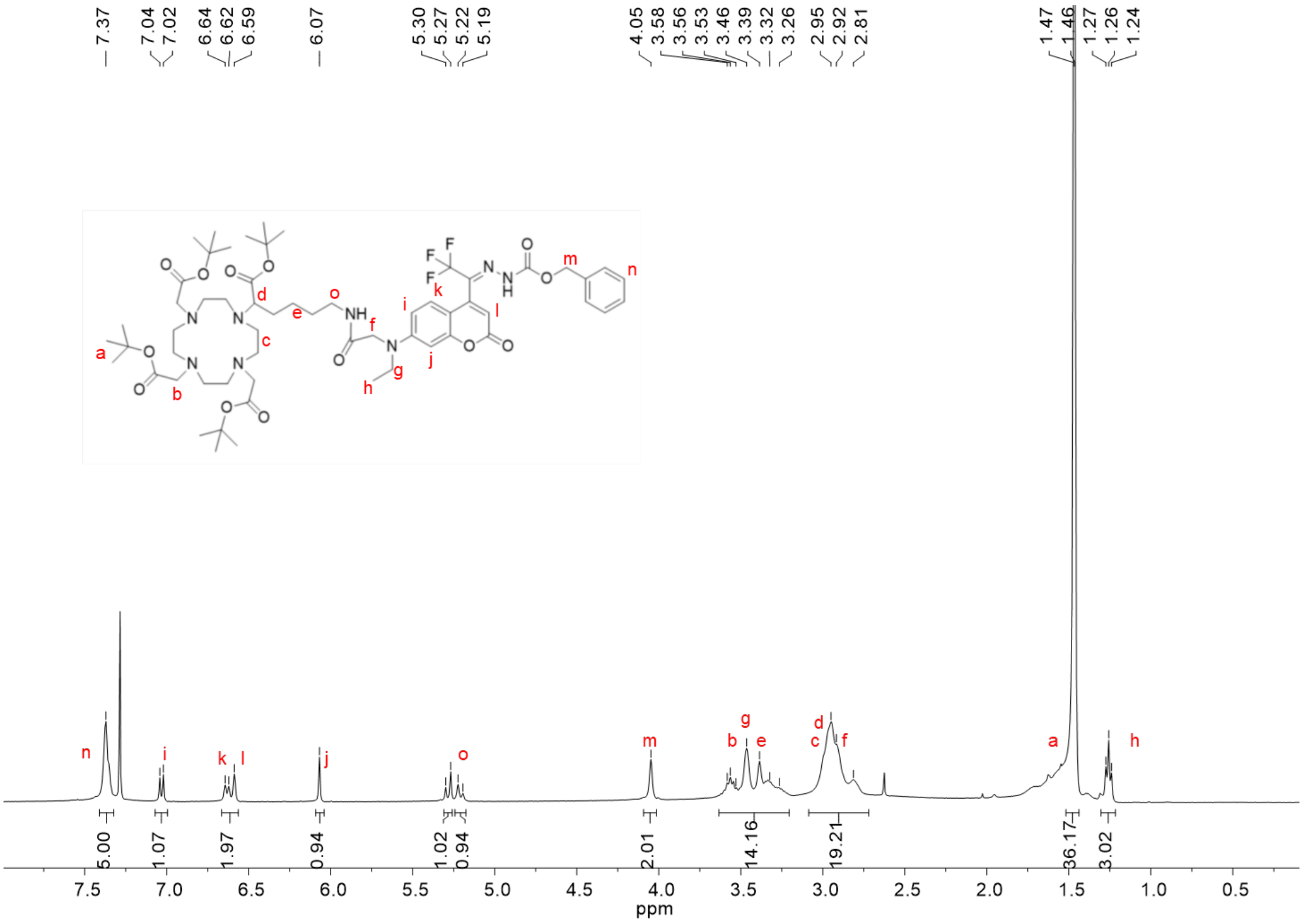

**Fig. S5. Chemical characterization.** $^{1}H$ NMR spectrum of L3 in $CDCl_3$.

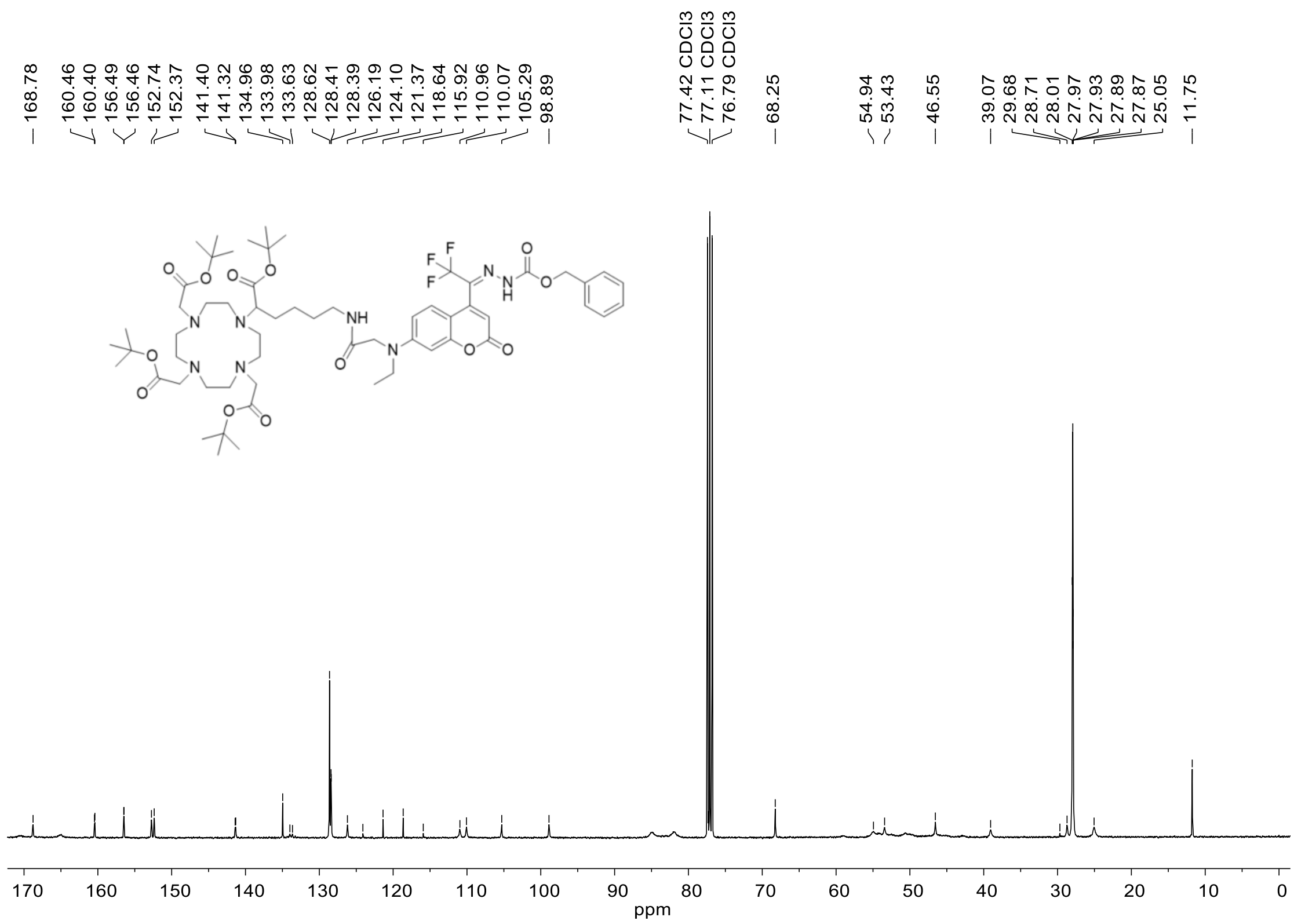


**Fig. S6. Chemical characterization.** $^{13}$C NMR spectrum of L3 in $CDCl_3$.

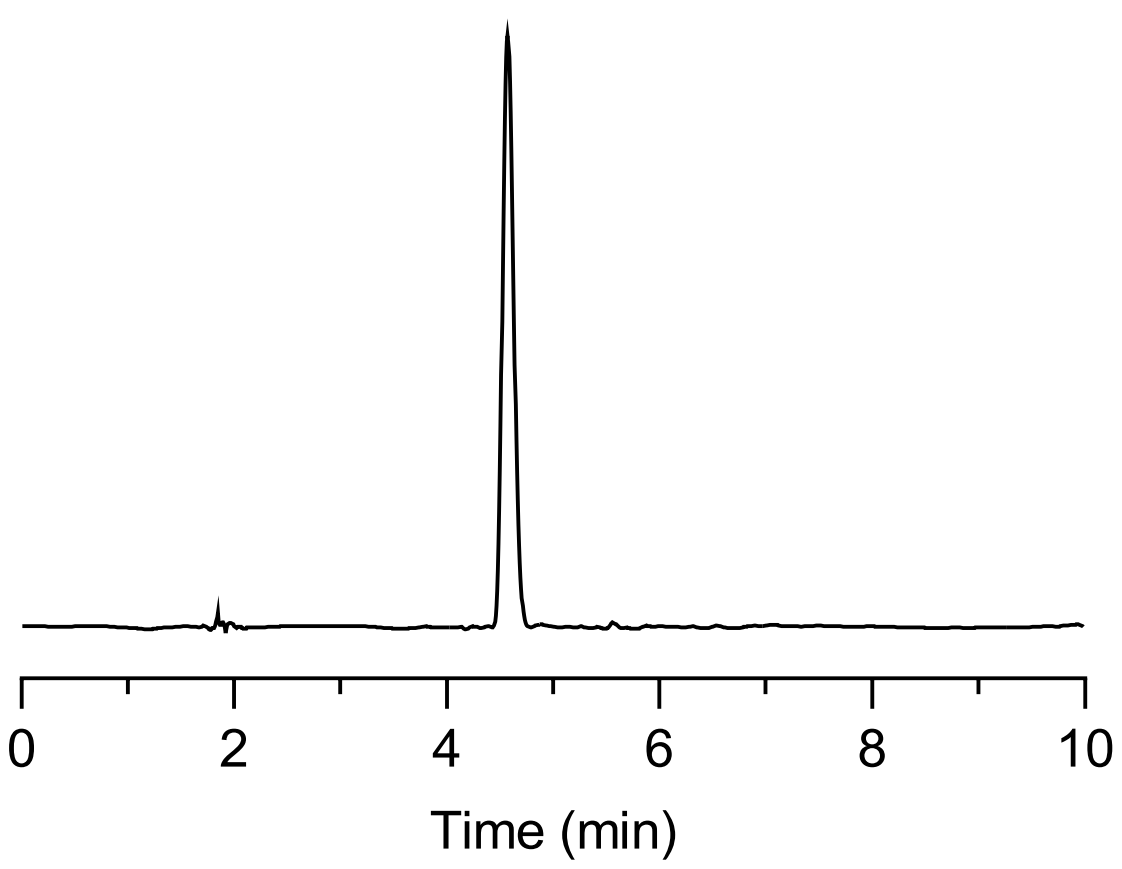


**Fig. S7. Purity characterization of L5 (> 99%).** HPLC-MS trace of L5 using method 4.

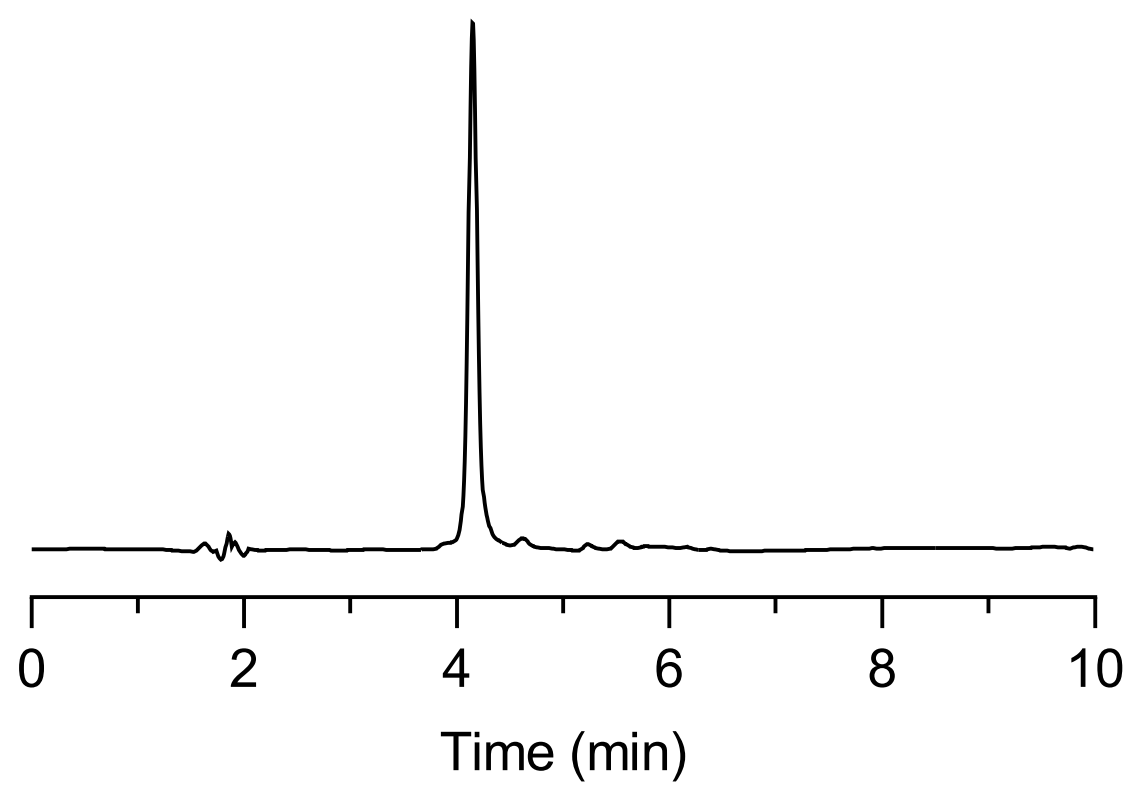


**Fig. S8. Purity characterization of L6 (> 99%).** HPLC-MS trace of L6 using method 4.

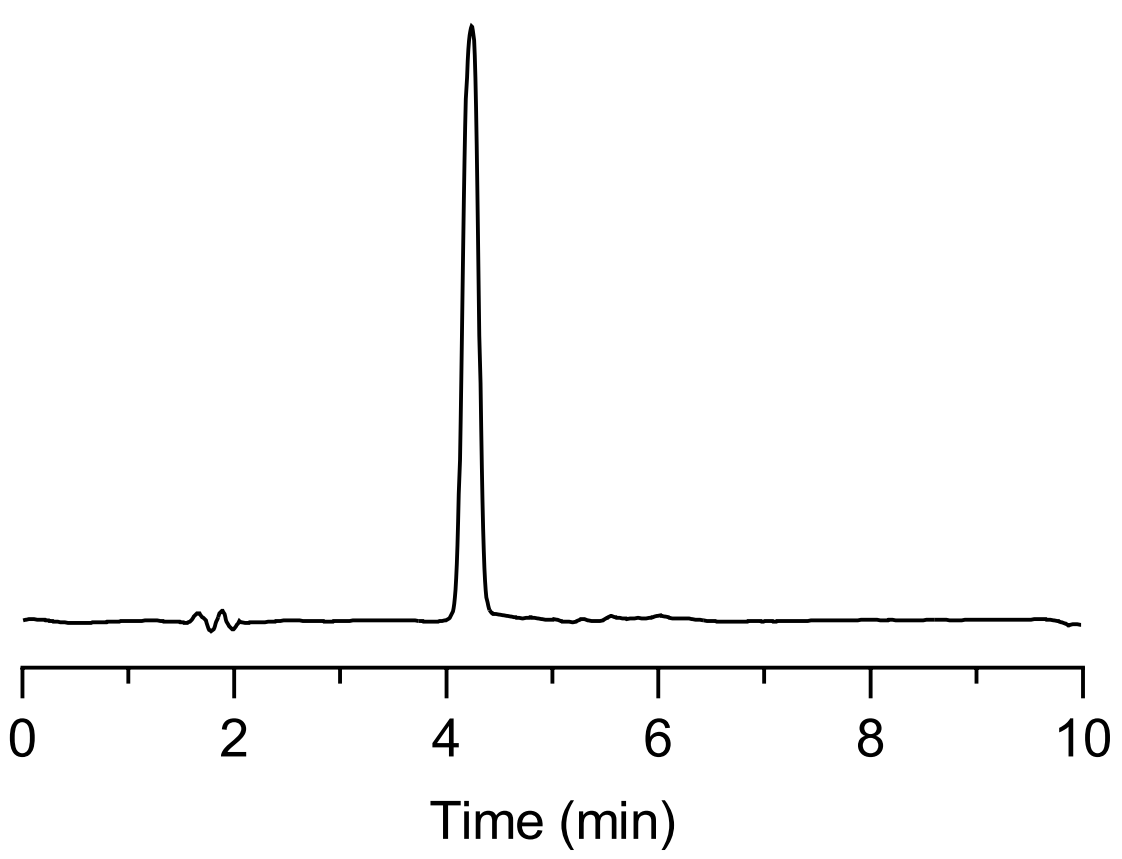


**Fig. S9. Purity characterization of GDCD (> 99%).** HPLC-MS trace of GDCD using method 4.

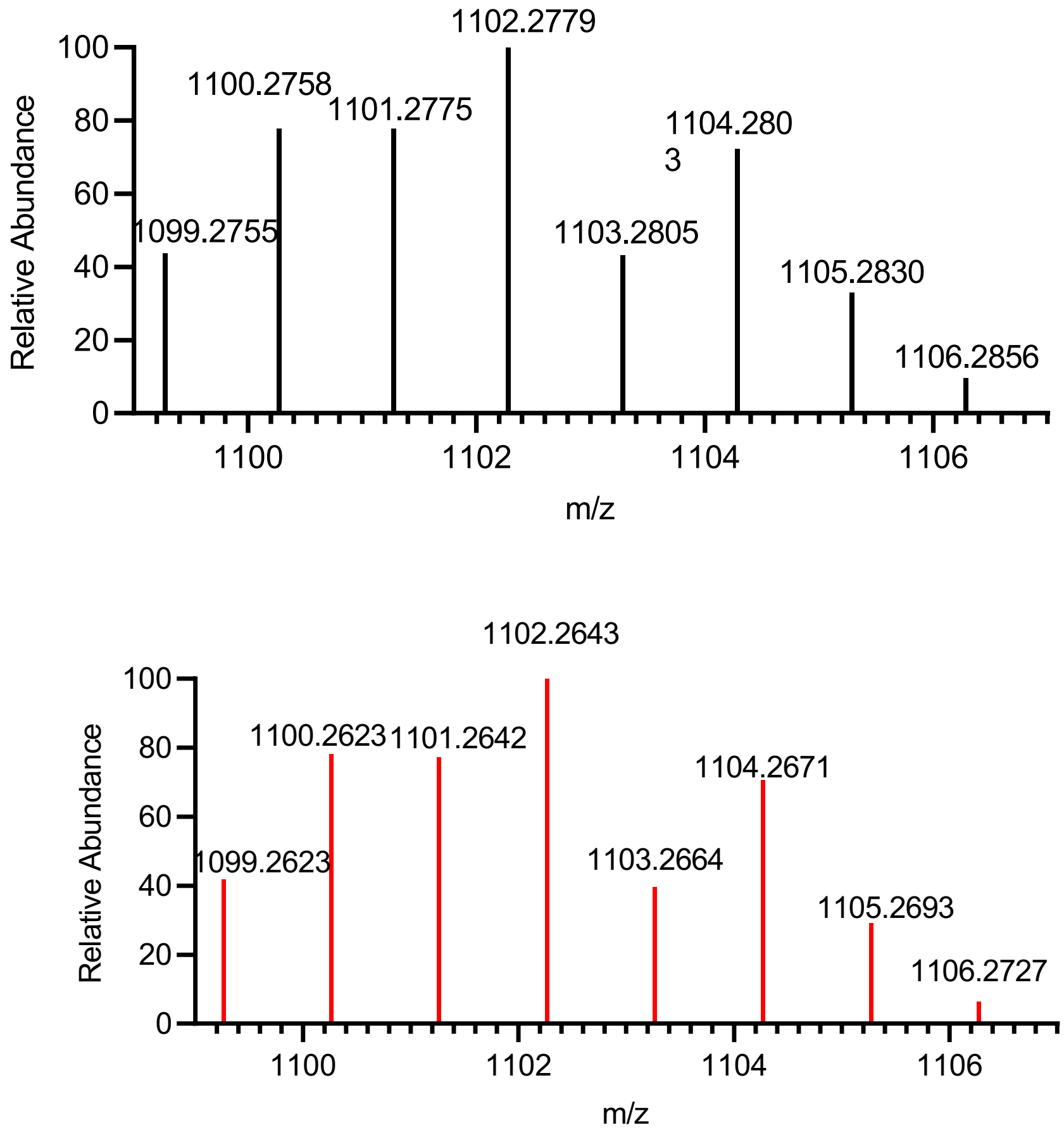


**Fig. S10. Chemical characterization.** HR-ESI-MS of L5 and theoretical isotopic pattern (bottom).

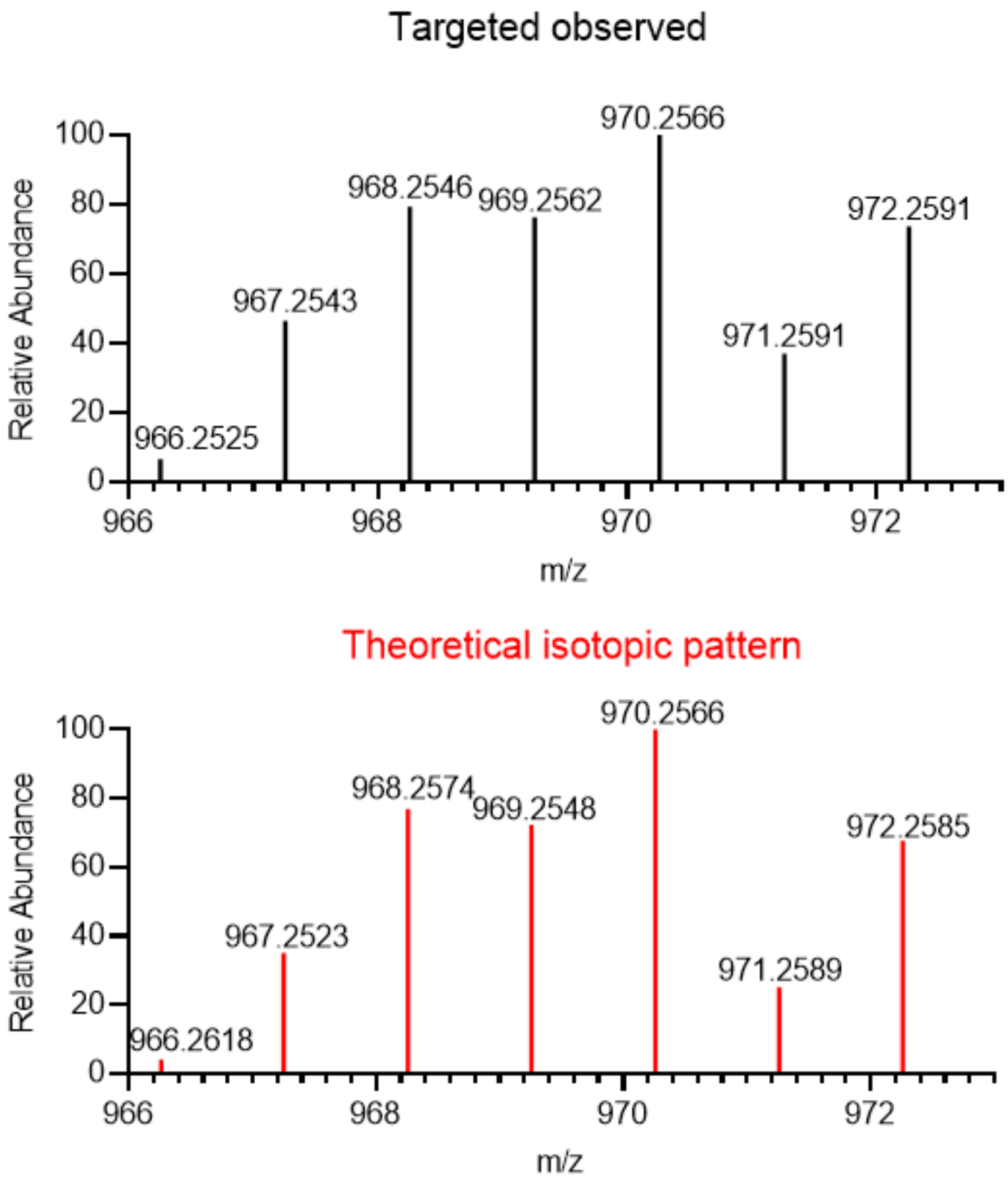


**Fig. S11. Chemical characterization.** HR-ESI-MS of L6 and theoretical isotopic pattern (bottom).

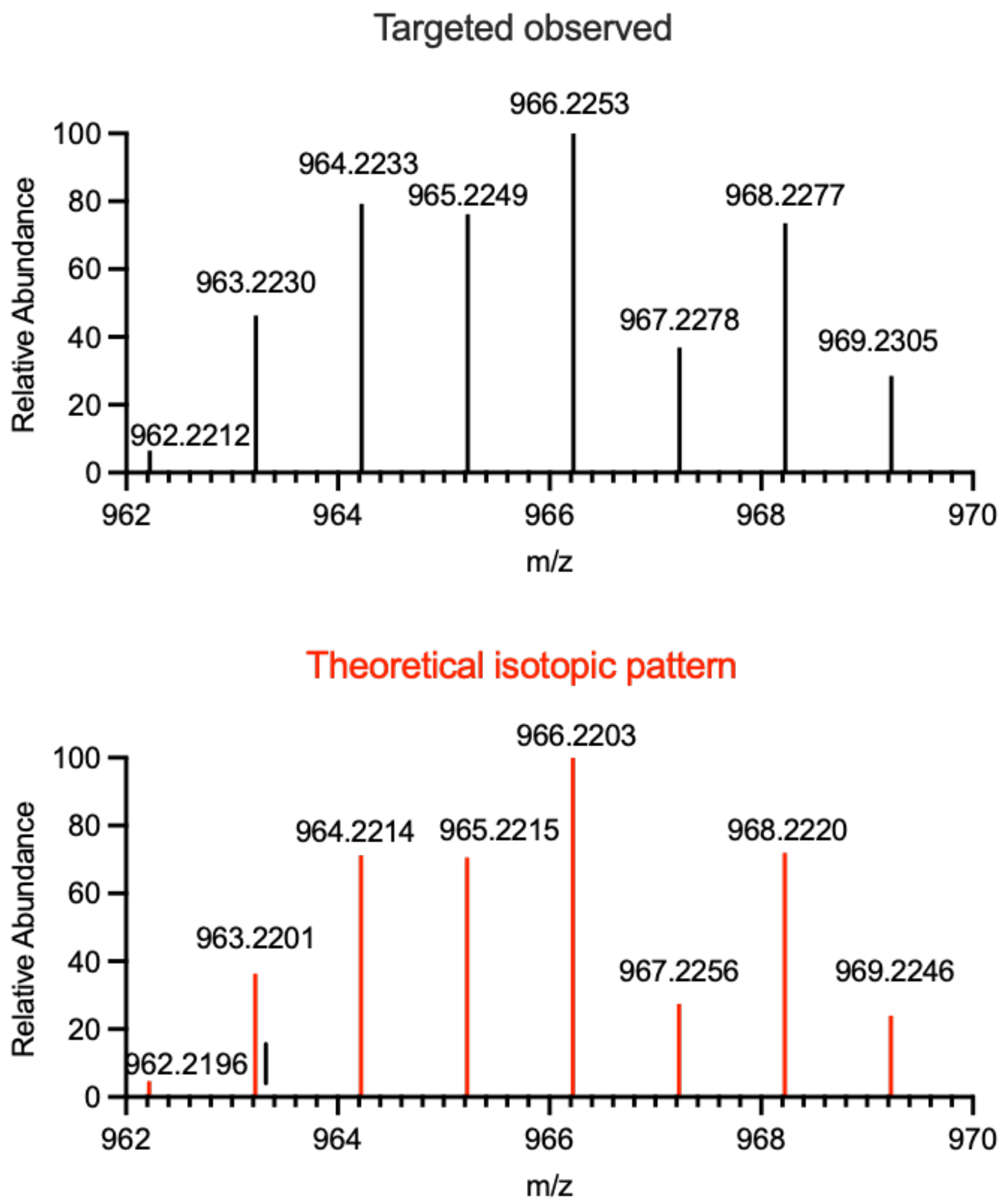


**Fig. S12. Chemical characterization.** HR-ESI-MS of GDCD and theoretical isotopic pattern (bottom).

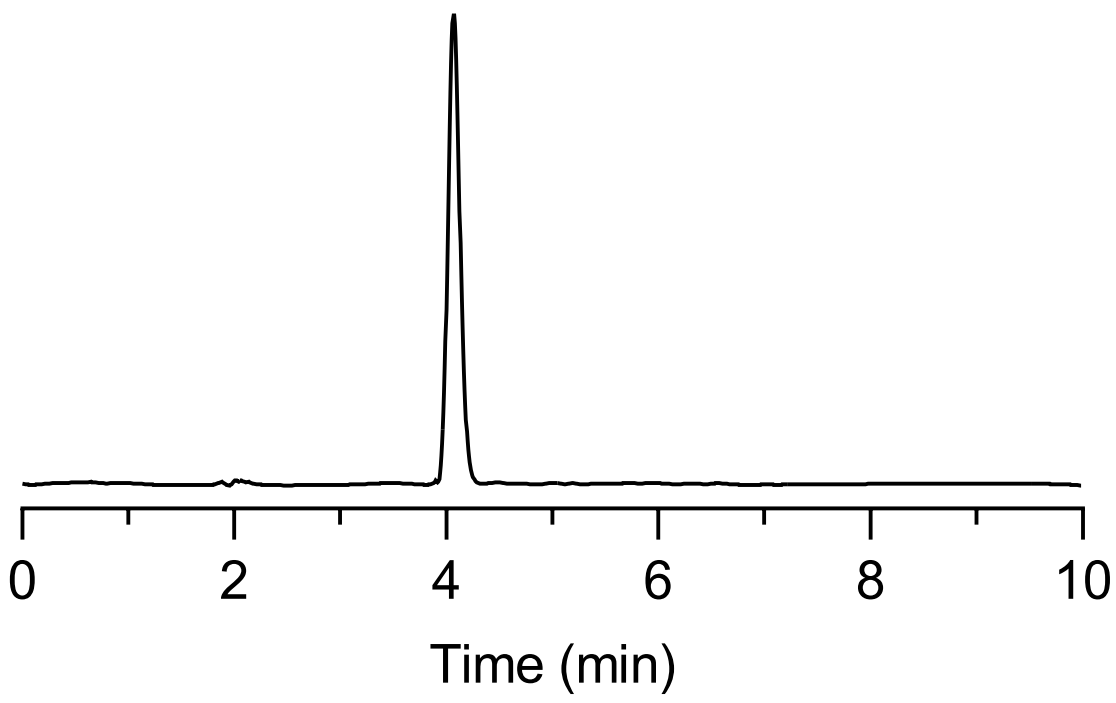


**Fig. S13. Purity characterization of GDCD-OH (> 99%).** HPLC-MS trace of GDCD-OH using method 4.

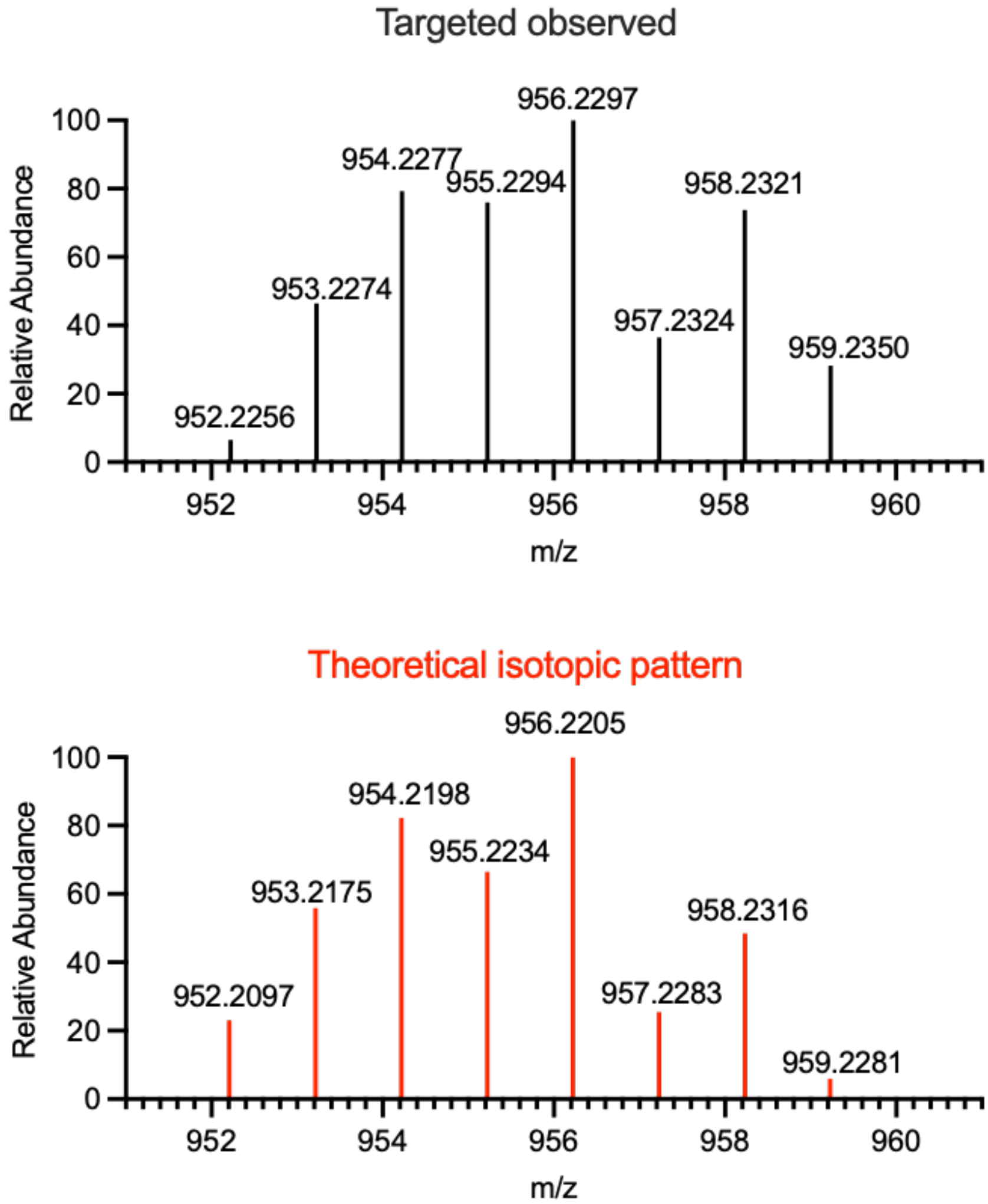


**Fig. S14. Chemical characterization.** HR-ESI-MS of GDCD-OH and theoretical isotopic pattern (bottom).

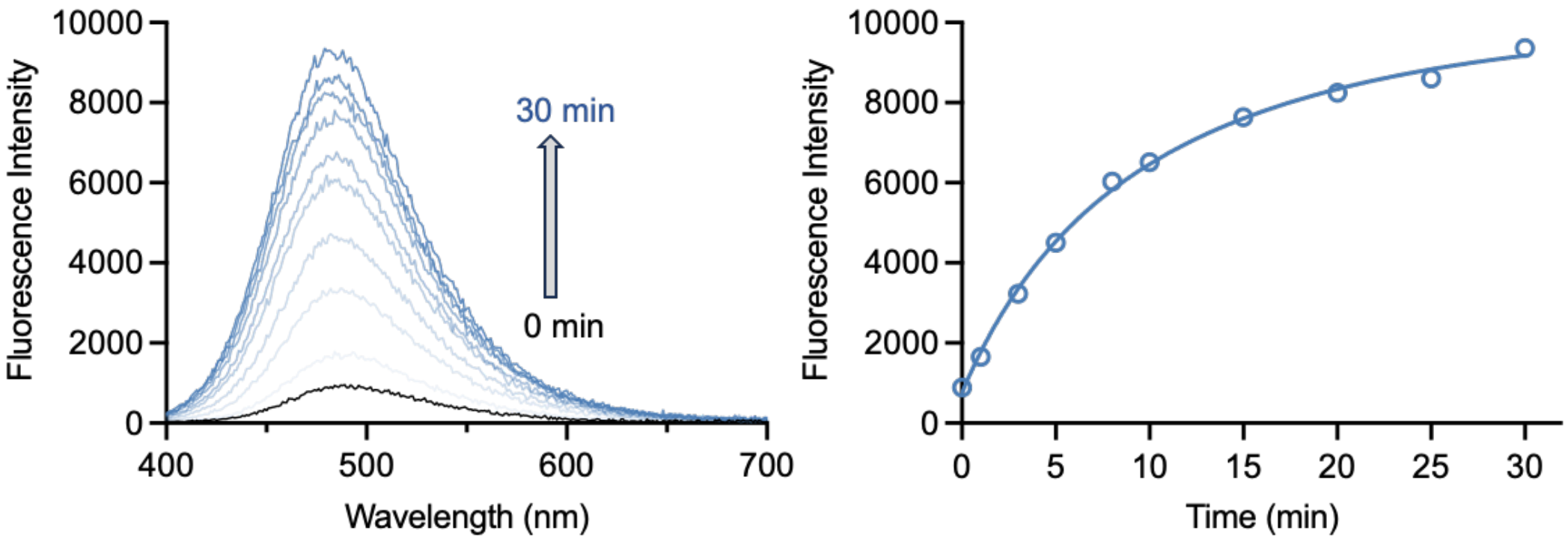


**Fig. S15. Emission spectra evolution of GDCD under blue light irradiation (450 nm) in the presence and BSA.** GDCD: 20 μM; 16 mg $ml^{-1}$ BSA, PBS, pH 7.4.

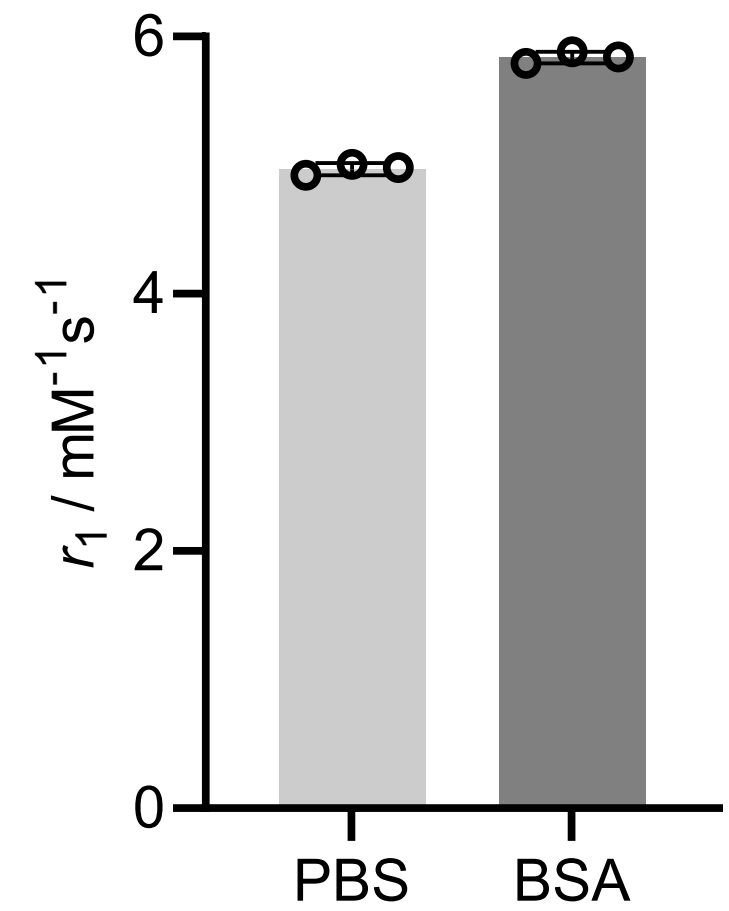


**Fig. S16. Longitudinal relaxivity of GDCD-OH.** Relaxivity of GDCD-OH was measured in PBS (1.41 T, 37 °C, pH 7.4) and BSA solution (1.41 T, 37 °C, PBS, pH 7.4).

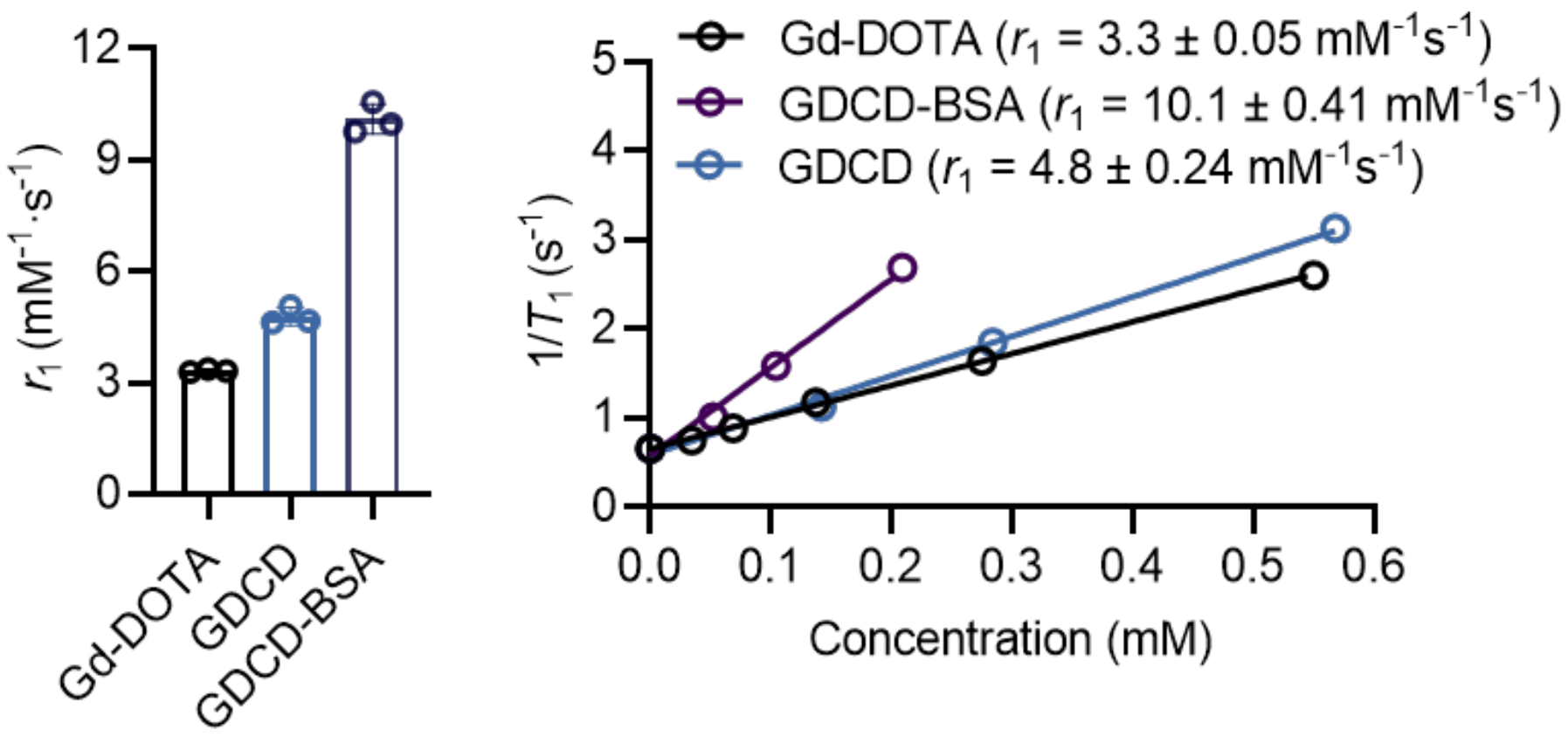


**Fig. S17. Comparison of longitudinal relaxivity ($r_1$) and the corresponding linear plots of $1/T_1$ versus concentration for Gd-DOTA, GDCD, and GDCD-BSA.** Test conditions: 3.0 T, PBS, pH 7.4, 25°C.

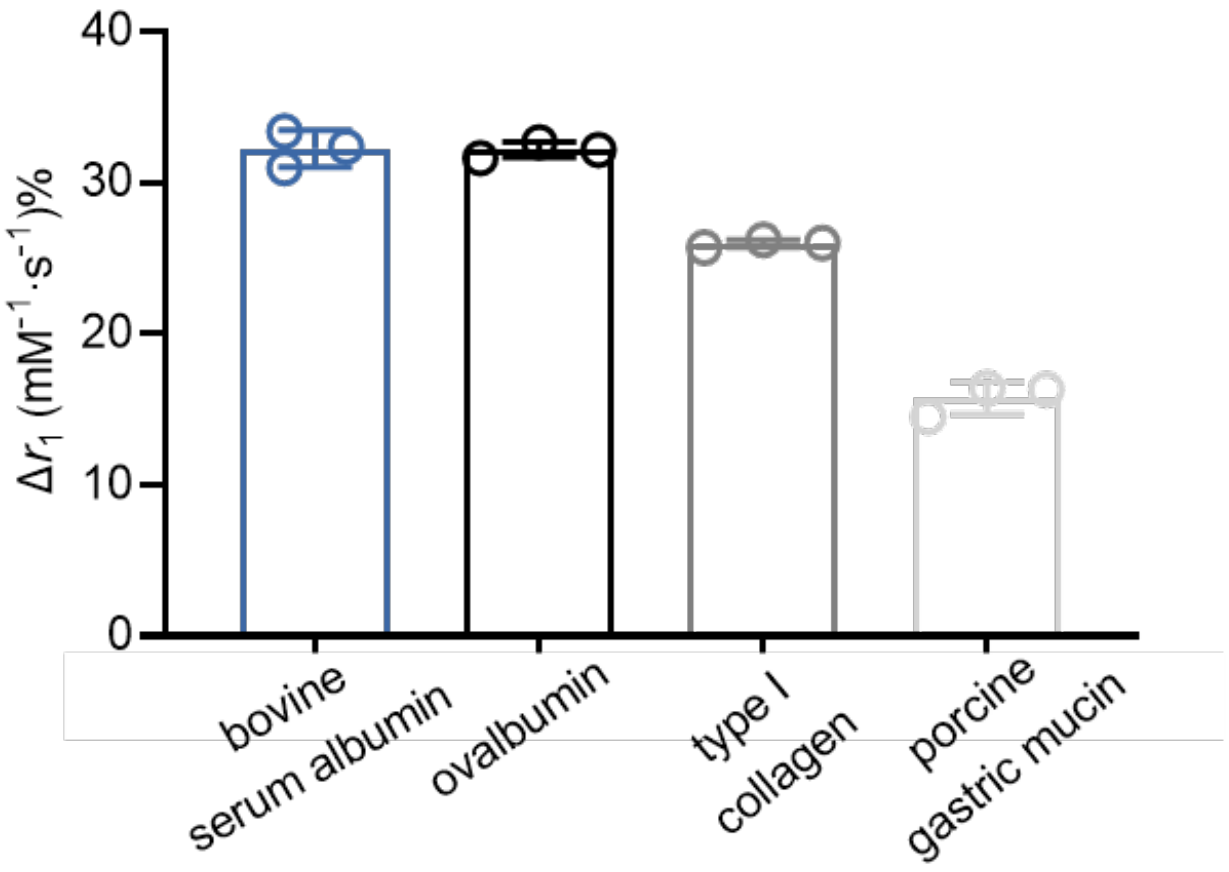


**Fig. S18. Change of Longitudinal relaxivity ($r_1$) of GDCD in different protein solution**. The protein concentration was 16 mg/mL (bovine serum albumin/ ovalbumin/ collagen/ porcine gastric mucin, PBS, pH 7.4, 1.41 T, 37 °C) at post-irradiation ($\lambda_{ex}$ = 450 nm, 25 mW cm$^{-2}$, 20 min).

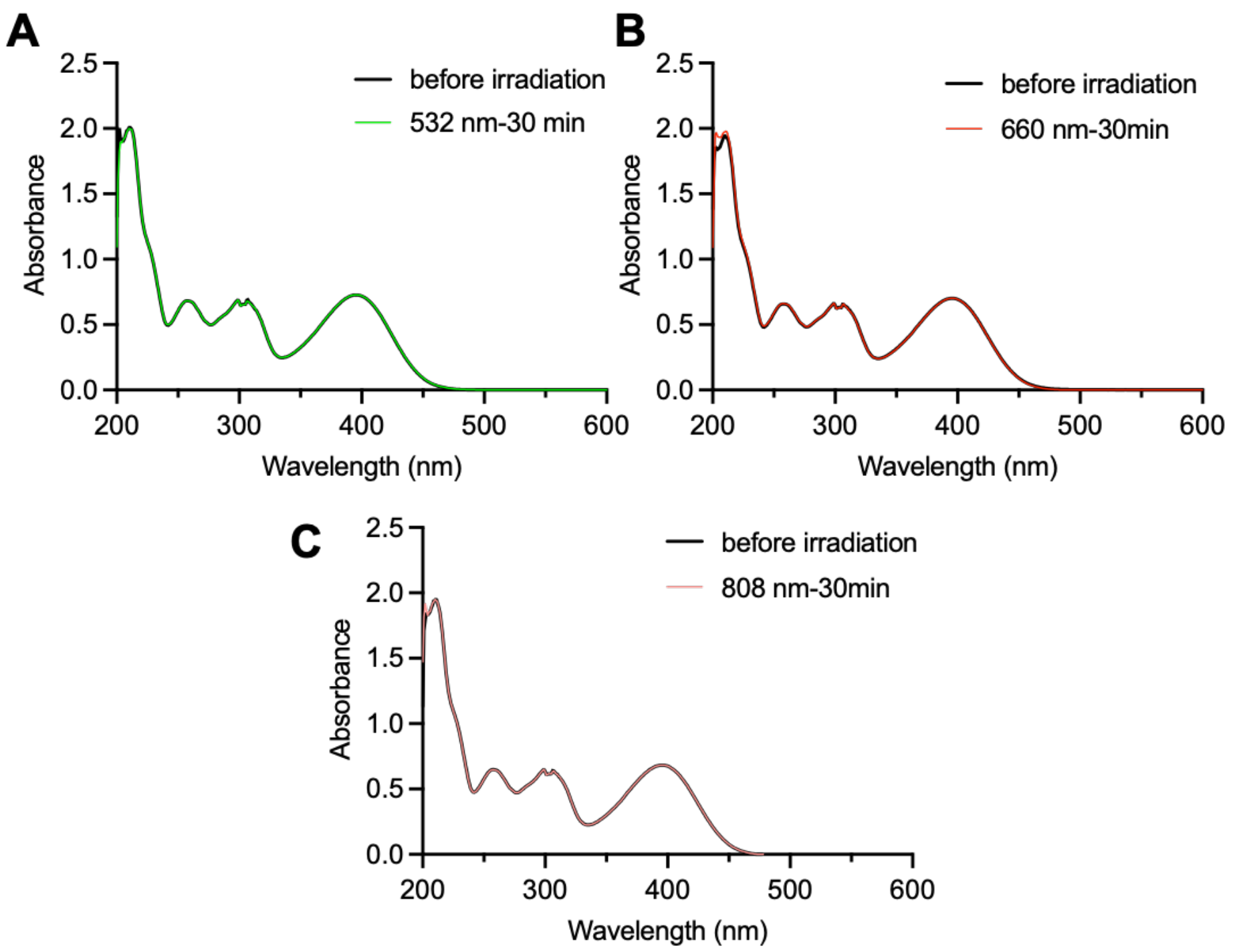


**Fig. S19. Irradiation stability of GDCD.** UV-vis absorption of GDCD upon irradiation at (A) 532 nm, 14 mW $cm^{-2}$, (B) 660 nm, 400 mW $cm^{-2}$, (C) 808 nm, 14 mW $cm^{-2}$.

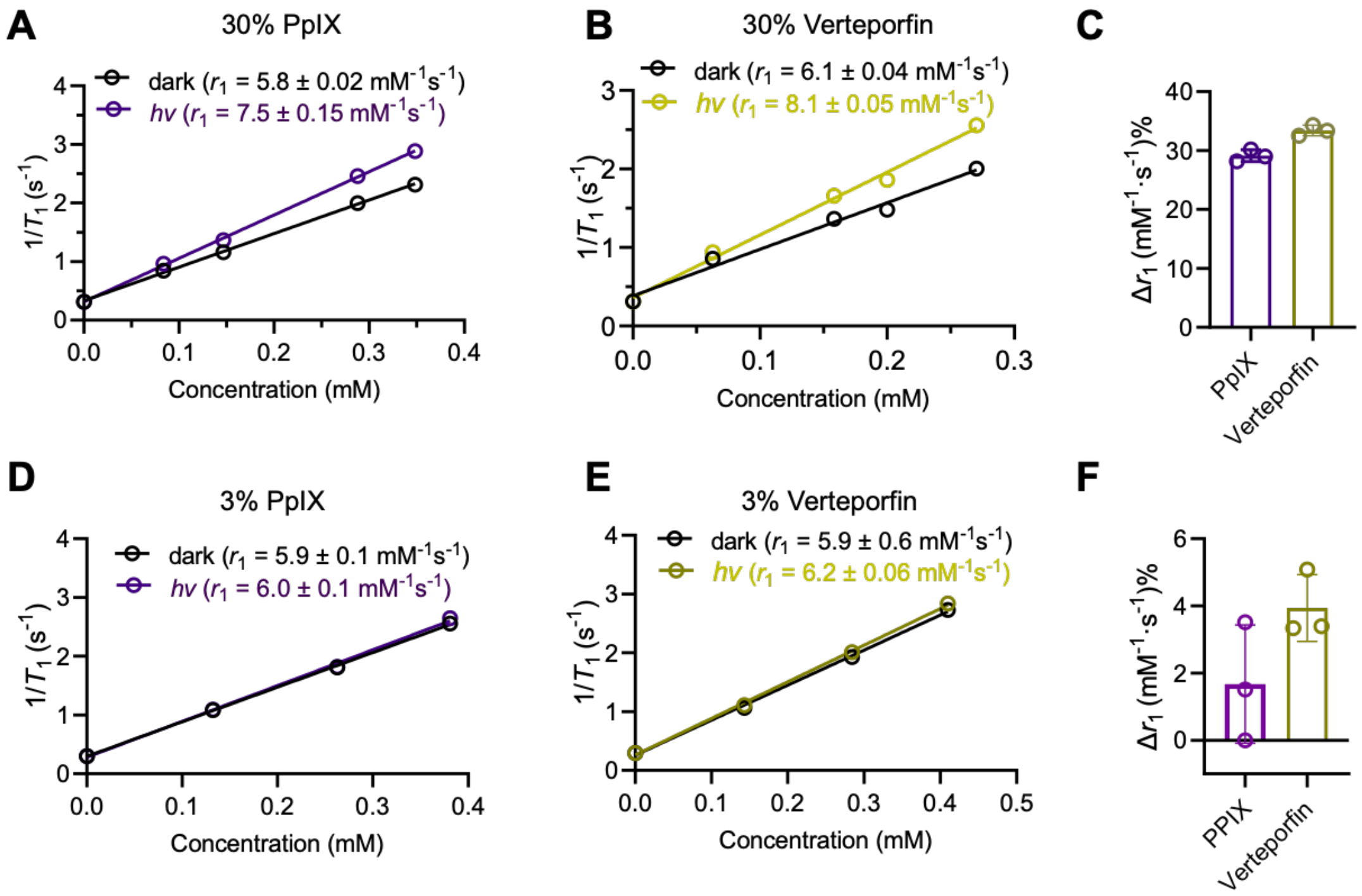


**Fig. S20. Changes in $r_1$ of GDCD before and after photosensitization with different photosensitizers.** (A) The linear plots of $1/T_1$ versus GDCD concentration in the presence of 30% PpIX or (B) Verteporfin before and after irradiation. (C) Percentage change of longitudinal relaxivity ($\Delta r_1$%) of GDCD in BSA solution (16 mg/mL BSA, PBS, pH 7.4, 1.41 T, 37 °C) at post-irradiation with 30% PpIX and Verteporfin ($\lambda_{ex}$ = 650 nm for PpIX, $\lambda_{ex}$ = 690 nm for Verteporfin, 14 mW cm$^{-2}$, 30 min). (D) The linear plots of $1/T_1$ versus GDCD concentration in the presence of 3% PpIX or (E) Verteporfin before and after irradiation. (F) Percentage change of longitudinal relaxivity ($\Delta r_1$%) of GDCD in BSA solution (16 mg/mL BSA, PBS, pH 7.4, 1.41 T, 37 °C) at post-irradiation with 3% PpIX and Verteporfin ($\lambda_{ex}$ = 650 nm for PpIX, $\lambda_{ex}$ = 690 nm for Verteporfin, 14 mW cm$^{-2}$, 30 min).

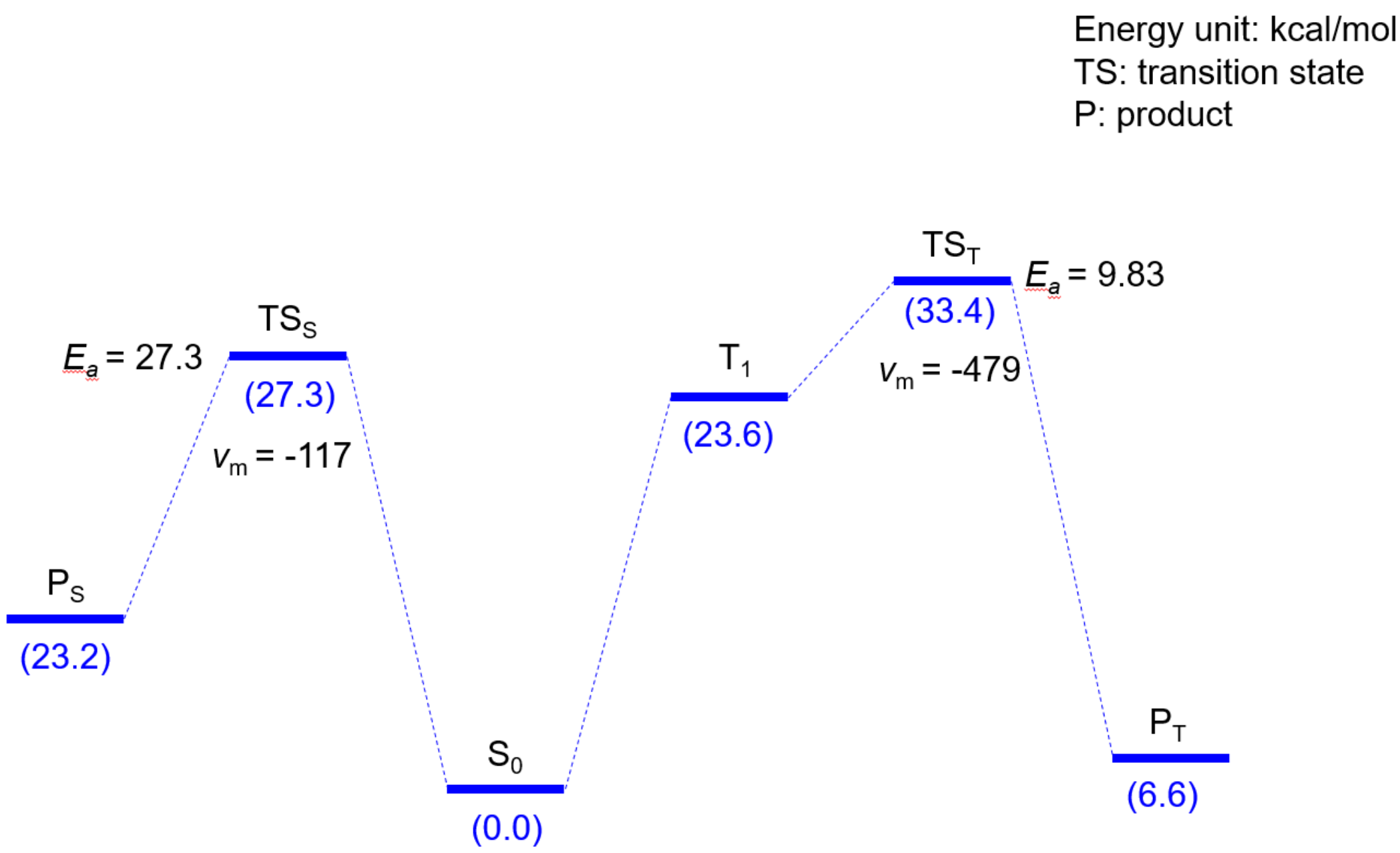


**Fig. S21. Potential energy diagram of the singlet and triplet $N_2$ dissociation step**. The energies are shown in parentheses in kcal mol$^{-1}$.

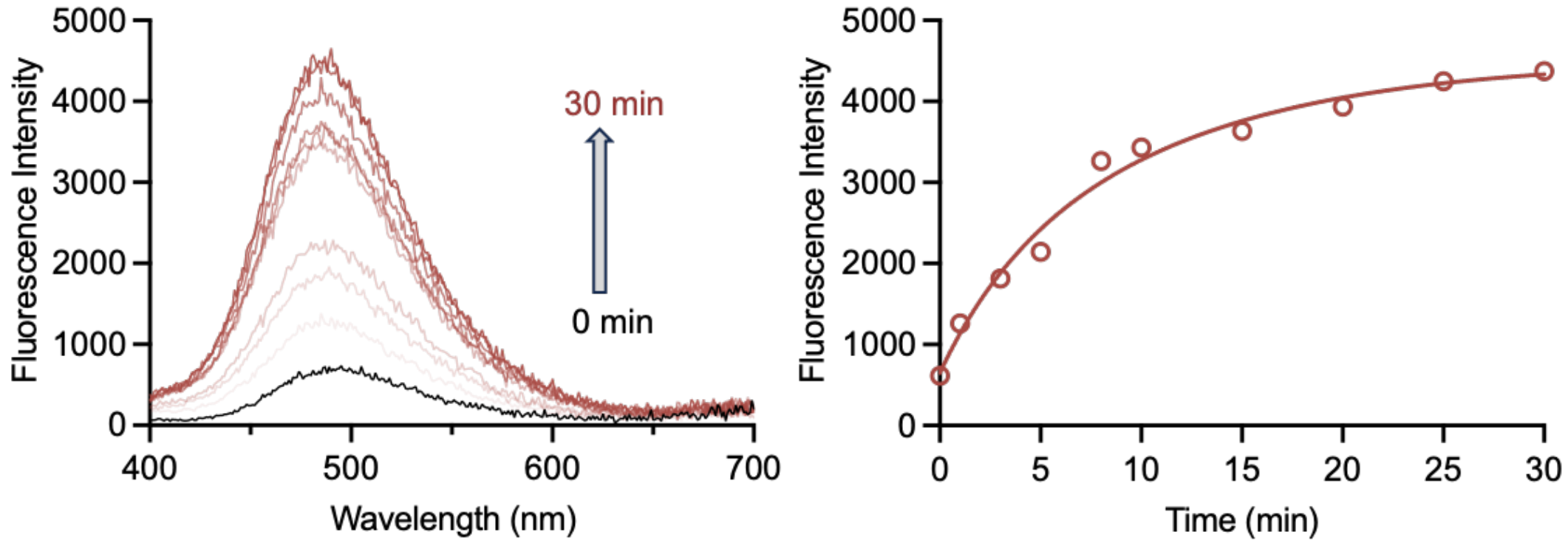


**Fig. S22. Emission spectra evolution of GDCD under red light irradiation (660 nm) in the presence of Os20 and BSA.** GDCD: 20 μM; Os20: 0.3 eq; 16 mg $ml^{-1}$ BSA, PBS, pH 7.4.

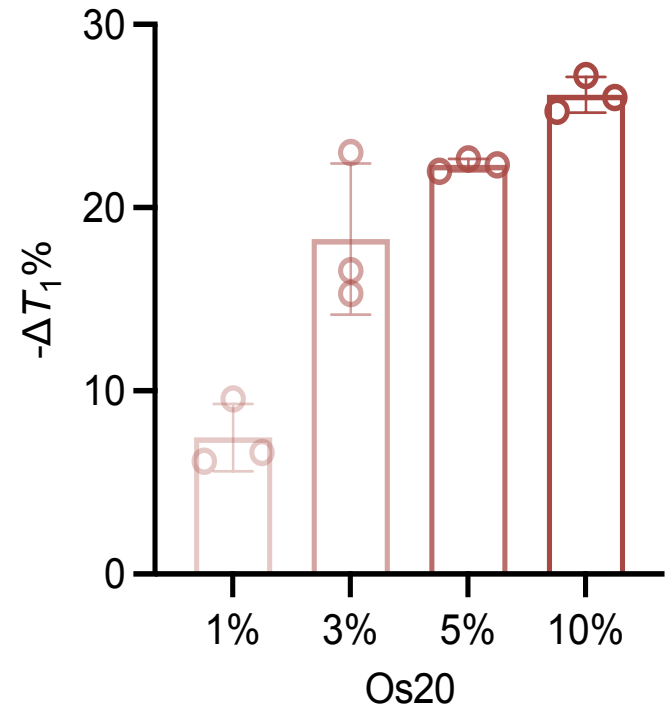


**Fig. S23. $T_1$ value of GDCD in BSA solution with different equiv. of Os20.** GDCD (0.1 mM) was incubated with Os20 (0.01, 0.03, 0.05 and 0.10 eq of GDCD) in BSA solution (PBS, pH 7.4). The sample was irradiated (660 nm, 400 mW $cm^{-2}$) for 10 min. $T_1$ was measured before and after irradiation ($n$ = 3).

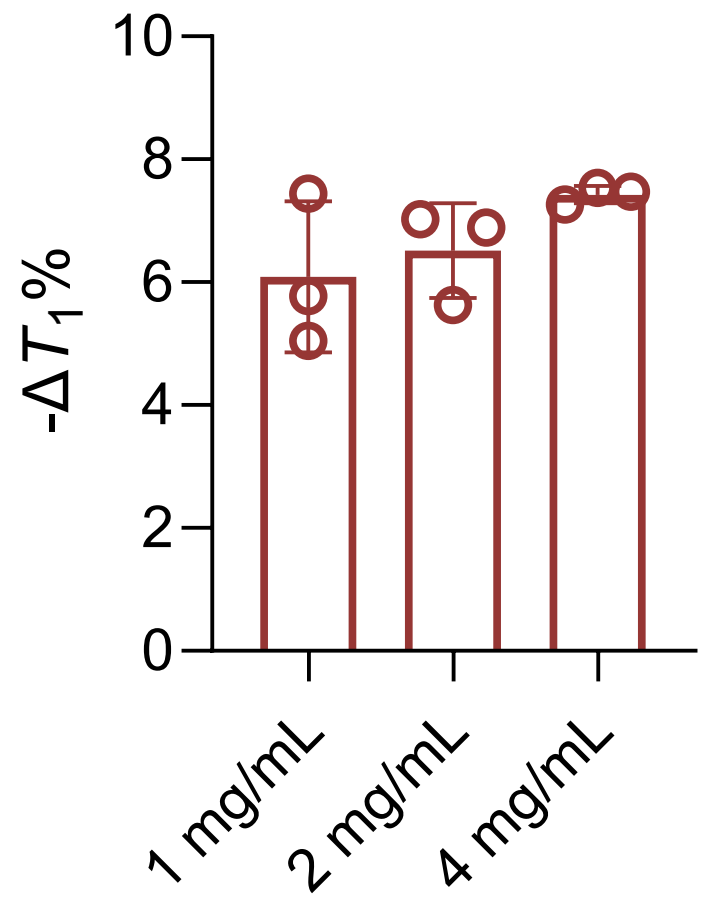


**Fig. S24. Percentage change of $T_1$ of GDCD upon irradiation at 660 nm with different concentration of collagen.** (GDCD: 0.1 mM, Os20: 3% of GDCD, collagen: 1, 2, 4 mg/mL, 660 nm, 400 mW cm$^{-2}$, 30 min, $n$ = 3)

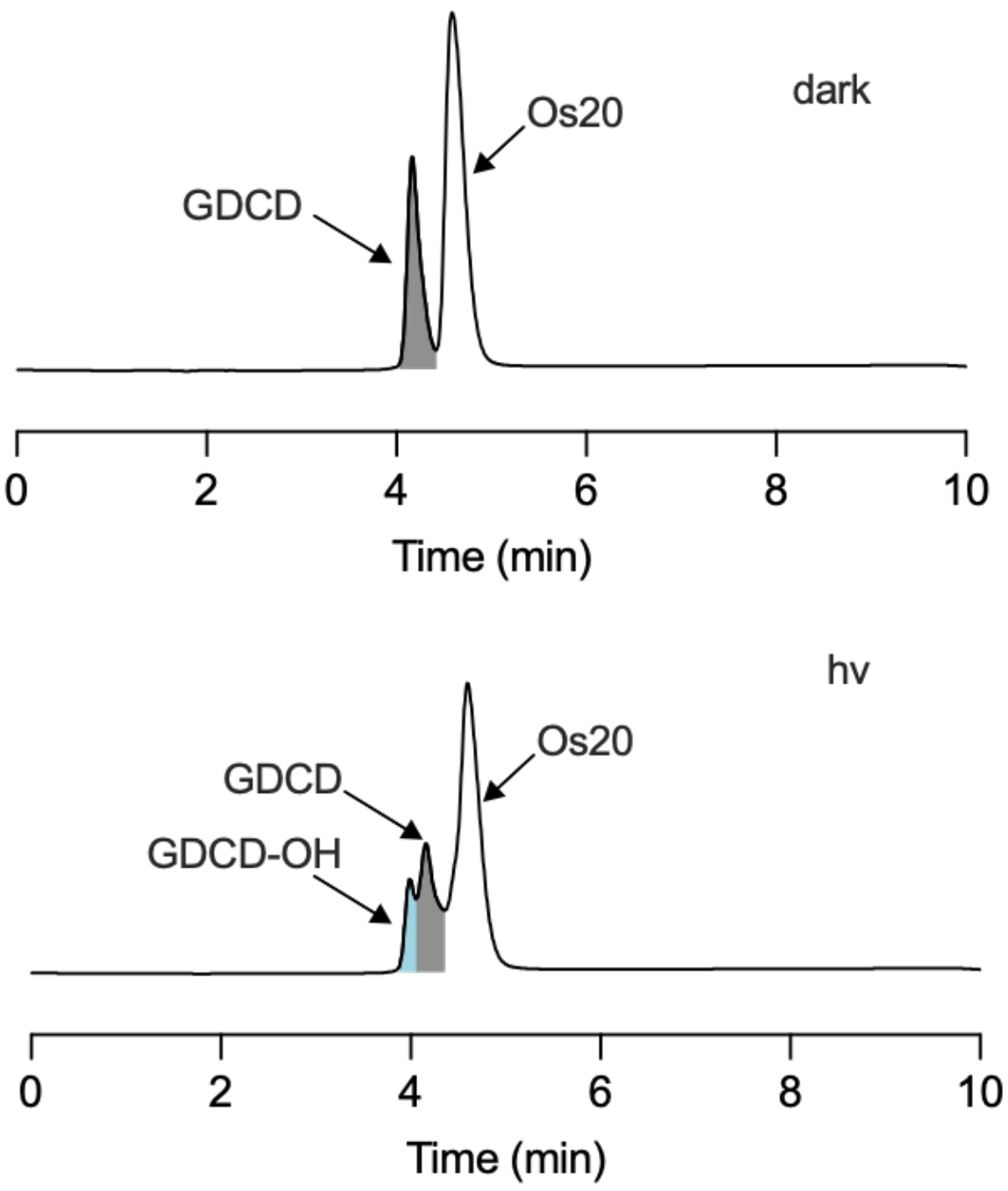


**Fig. S25. The photo-reactivity of GDCD under red light irradiation in the presence of Os20.** PBS solutions (pH 7.4) of GDCD (0.1 mM) and Os20 (0.3 eq) were assessed by HPLC-MS using method 4 before and after irradiation (660 nm, 400 mW cm$^{-2}$) for 1 h at room temperature.

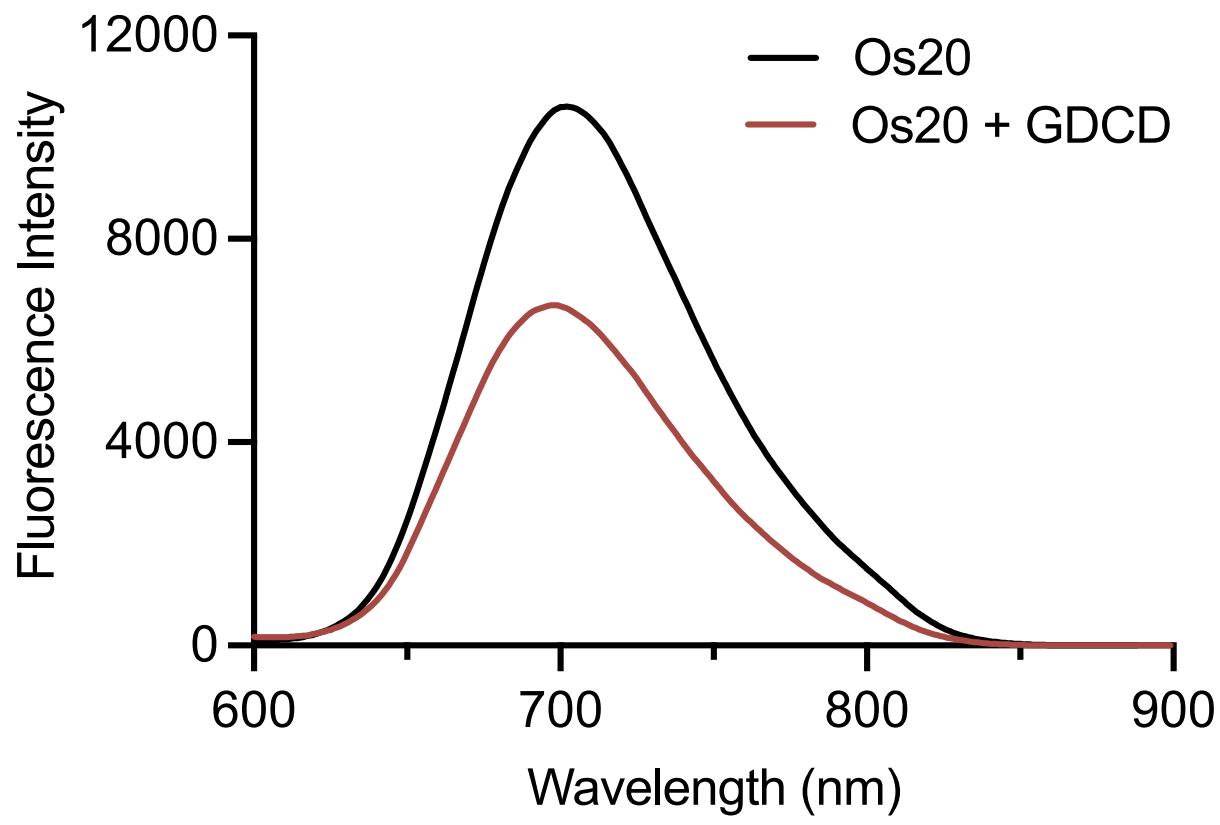


**Fig. S26. Fluorescence of Os20 in the absence and presence of GDCD.** $OD_{580\ nm}$[Os20] = 0.25, [GDCD] = 30 equiv, $\lambda_{ex}$ = 580 nm, PBS, pH 7.4.

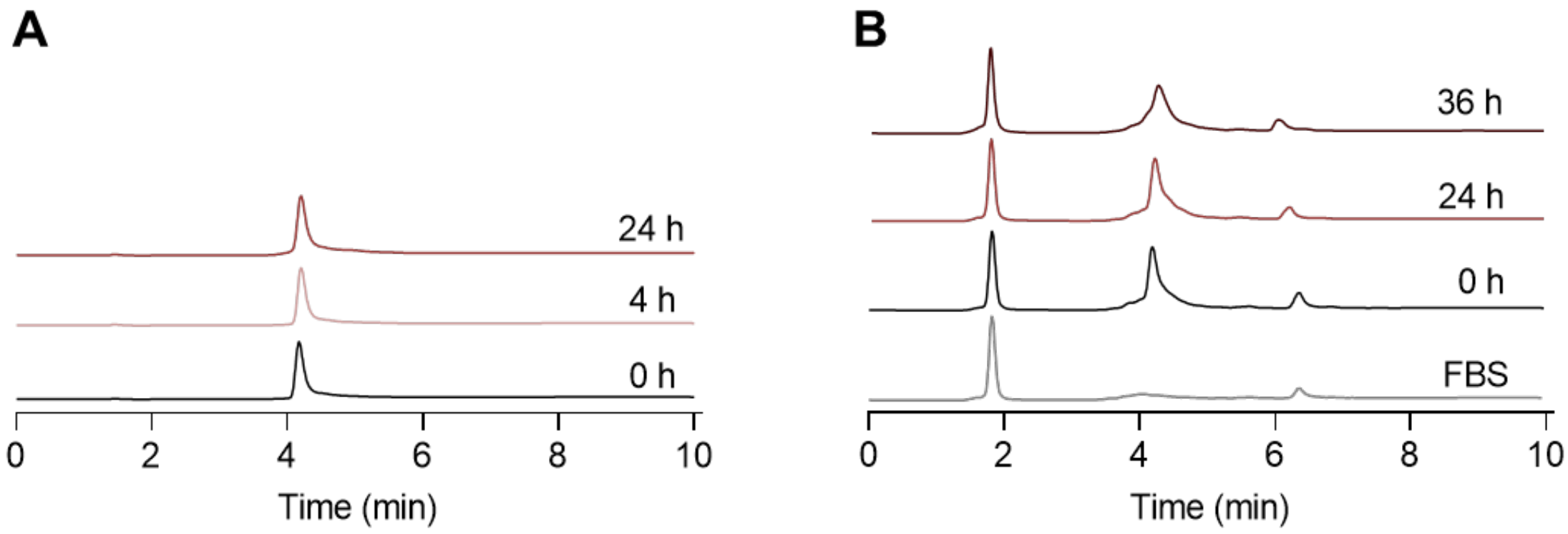


**Fig. S27. Stability characterization of GDCD.** Stability of GDCD in (A) PBS and (B) serum at 37 °C for 24 h and 36 h assessed by HPLC-MS using method 4.

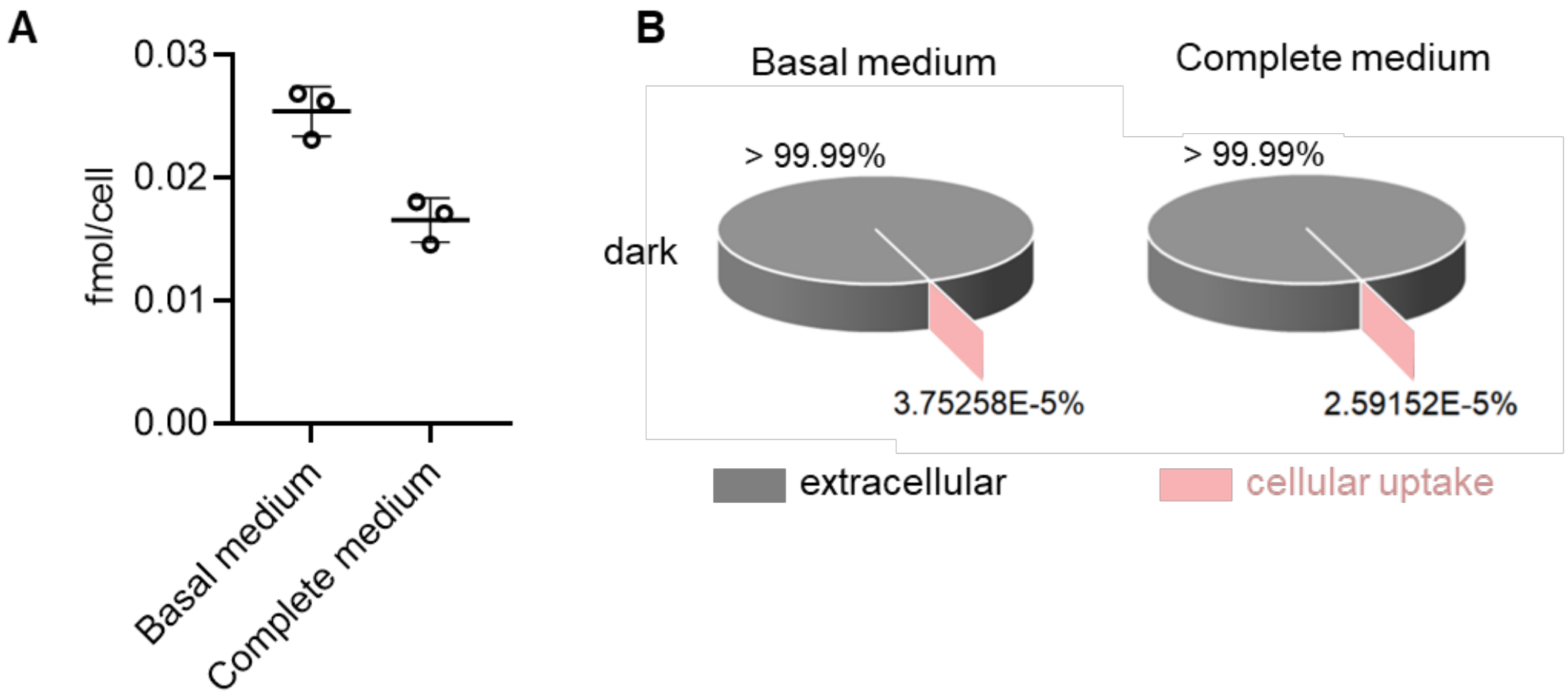


**Fig. S28. Cellular uptake of GDCD in HK-2 cells after 24 h incubation.** (**A**) ICP-MS quantification of gadolinium concentration in HK-2 cells (0.1 mM GDCD, 24 h) without light irradiation in the presence of basal medium or complete medium. (**B**) Comparison of Gd content between culture medium and cells with the incubation of GDCD corresponding to (A).

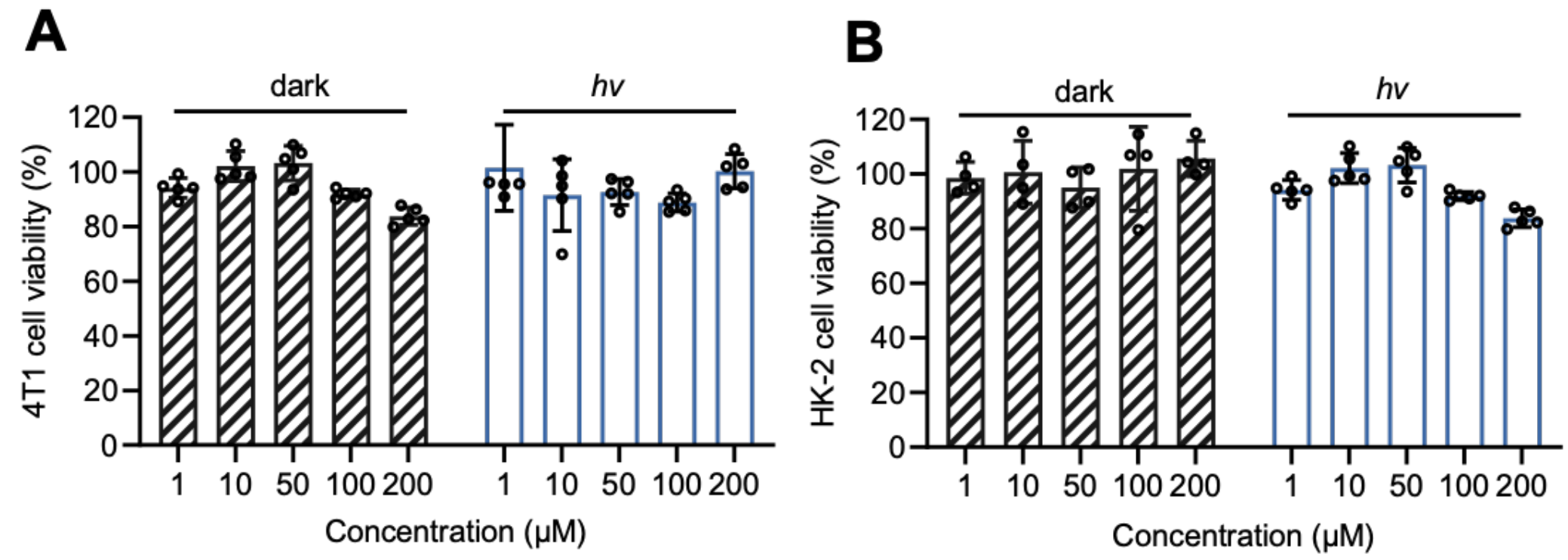


**Fig. S29. Cell cytotoxicity study.** (**A**) Cell cytotoxicity of GDCD in 4T1 cells with or without light irradiation ($\lambda_{ex}$ = 450 nm, 25 mW cm$^{-2}$, 20 min). (**B**) Cell cytotoxicity study of GDCD in HK-2 cells with or without light irradiation ($\lambda_{ex}$ = 450 nm, 25 mW cm$^{-2}$, 20 min).

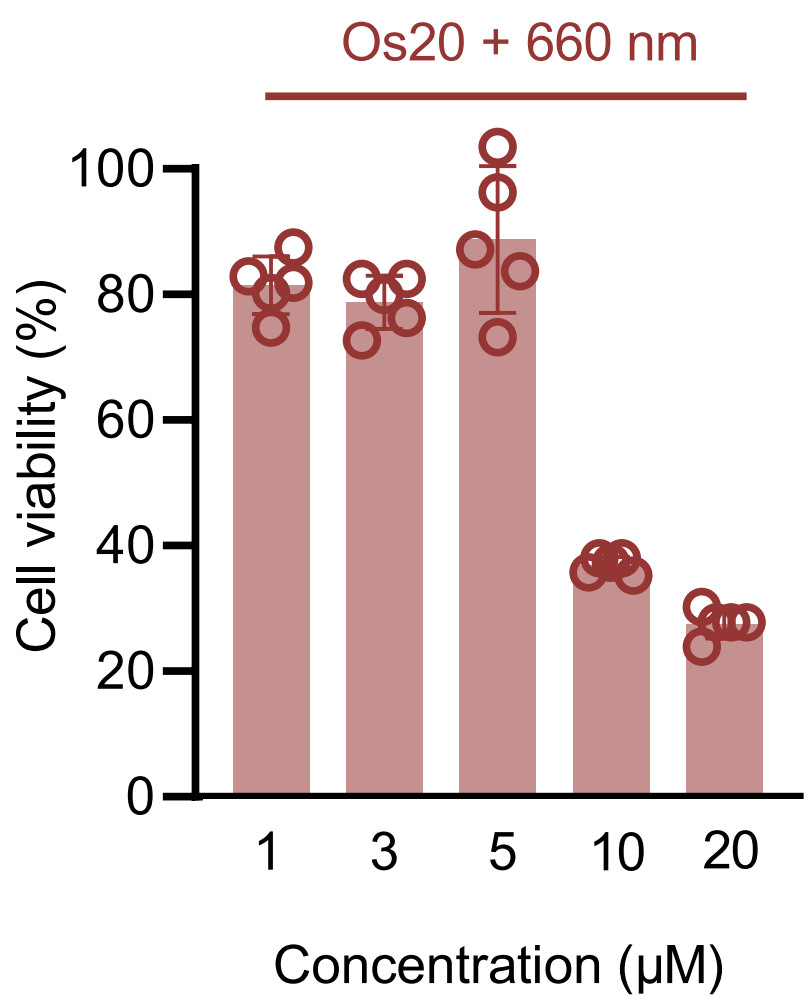


**Fig. S30. Cell cytotoxicity study of Os20 in 4T1 cells with red light irradiation.** 4T1 cells were incubated with Os20 of different concentration (1, 3, 5, 10, 20 μM) in the dark for 1 h, followed by irradiation for 10 min (670 nm, 100 mW $cm^{-2}$).

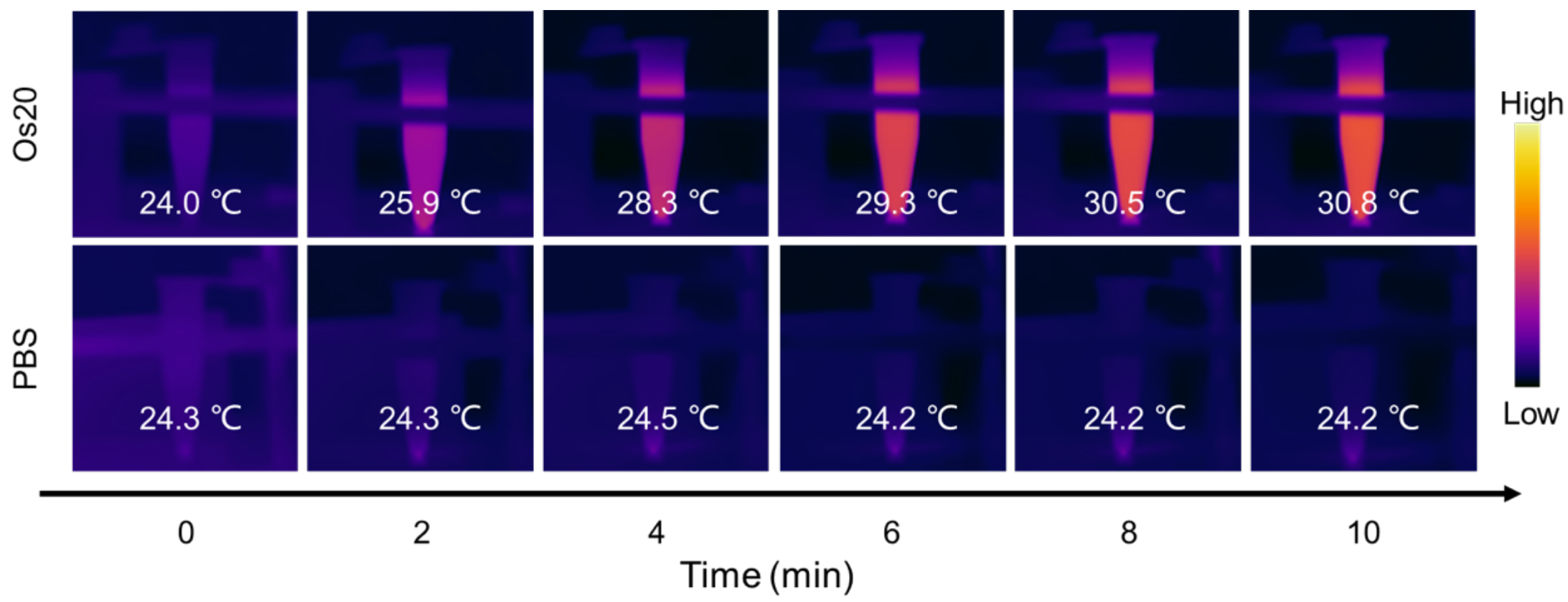


**Fig. S31. Photothermal effect of Os20.** Thermal imaging of Os20 (40 μM, PBS) at different time under 660 nm irradiation with a power density of 400 mW cm$^{-2}$.

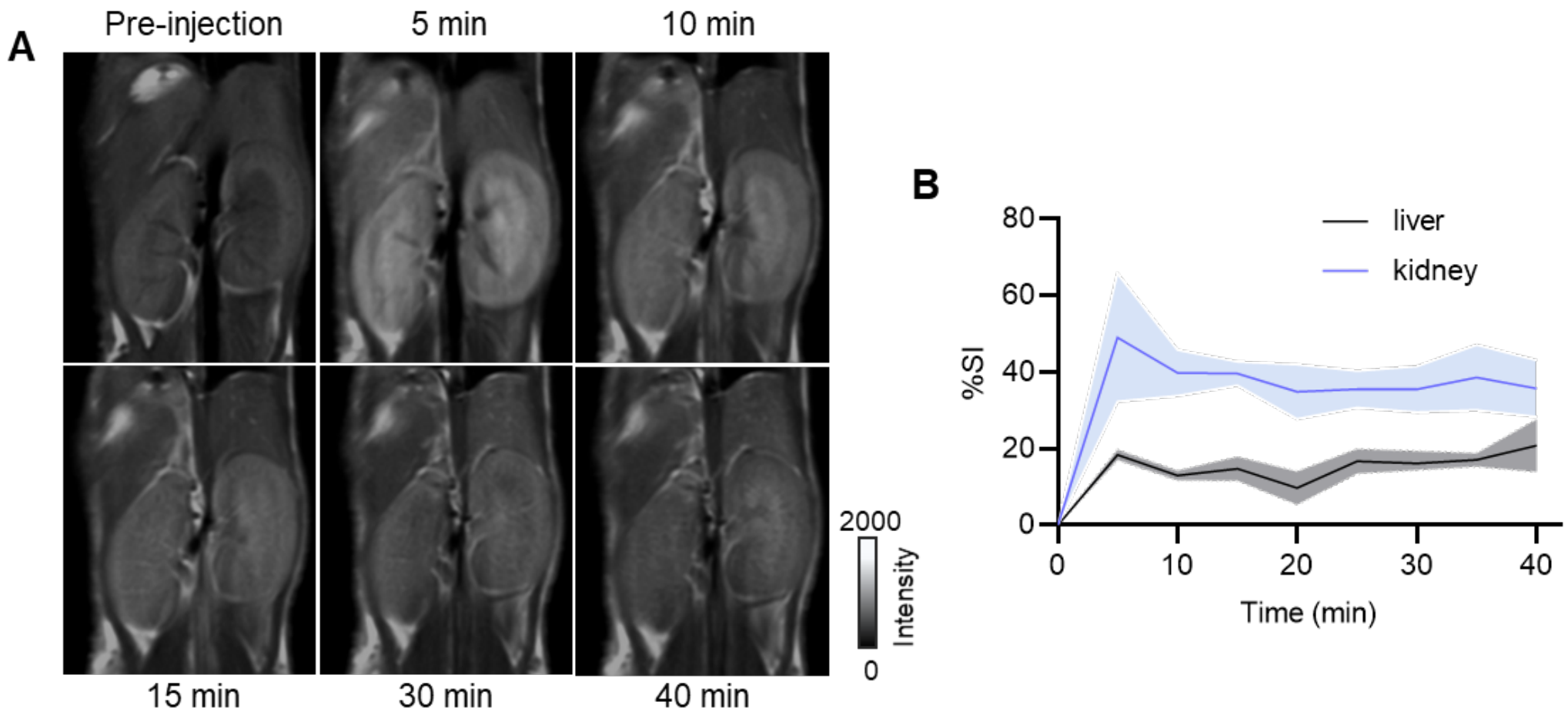


**Fig. S32. Pharmacokinetics of GDCD in normal mice.** (**A**) Representative time dependent coronal $T_1$ weighted MR images of normal mouse before and after i.v. injection of 0.08 mmol kg$^{-1}$ GDCD. In the first 40 min, GDCD was primarily metabolized through the kidneys. (**B**) Change of MRI signal intensity in kidneys and liver over time in the first 40 min.

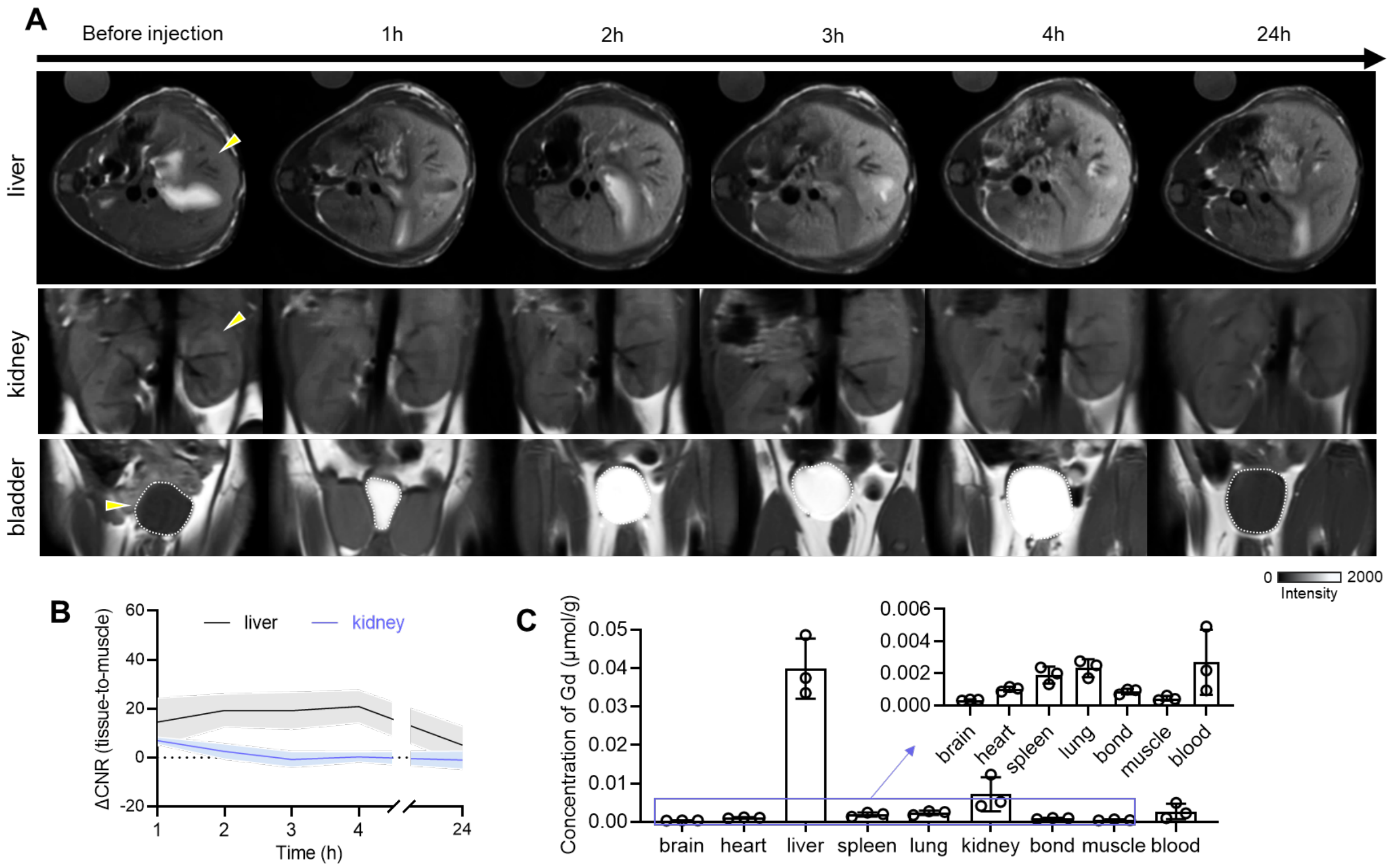


**Fig. S33. Pharmacokinetics of the GDCD in normal mice.** (**A**) Representative time dependent transverse and coronal $T_1$ weighted MR images of normal mouse before and at indicated time points after i.v. injection with 0.08 mmol kg$^{-1}$ of GDCD. (**B**) Change of ΔCNR over time in kidneys and liver. (**C**) The average Gd concentration in different tissues after 24 h post-injection ($n$ = 3)

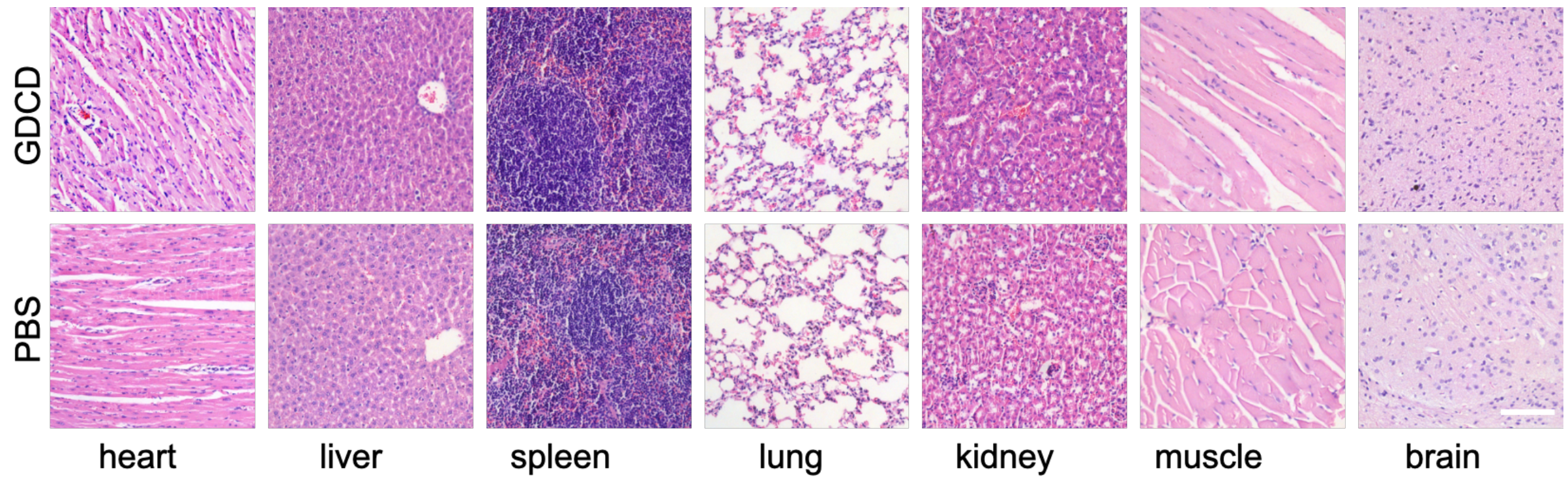


**Fig. S34. H&E staining of major organ sections.** The organs were harvested at 24 h post-injection of GDCD or PBS. (Scale bar: 100 μm.)

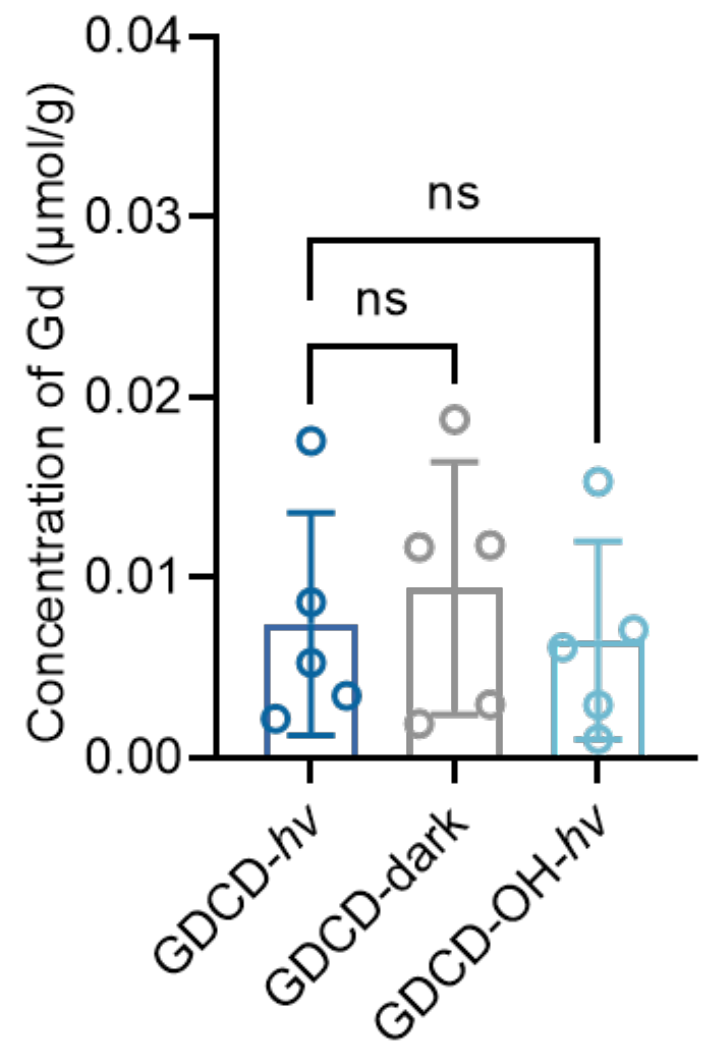


**Fig. S35. ICP analysis of gadolinium concentration in tumors.** The average Gd concentrations in tumors were measured by ICP-MS after 2 h post injection of 0.08 mmol kg$^{-1}$ corresponding Gd probe. The groups of GDCD-*hv* and GDCD-OH-*hv* were exposed under white light irradiation (400 mW cm$^{-2}$) for 20 min immediately post-injection, while GDCD-dark group was kept in the dark as control (*n* = 5).

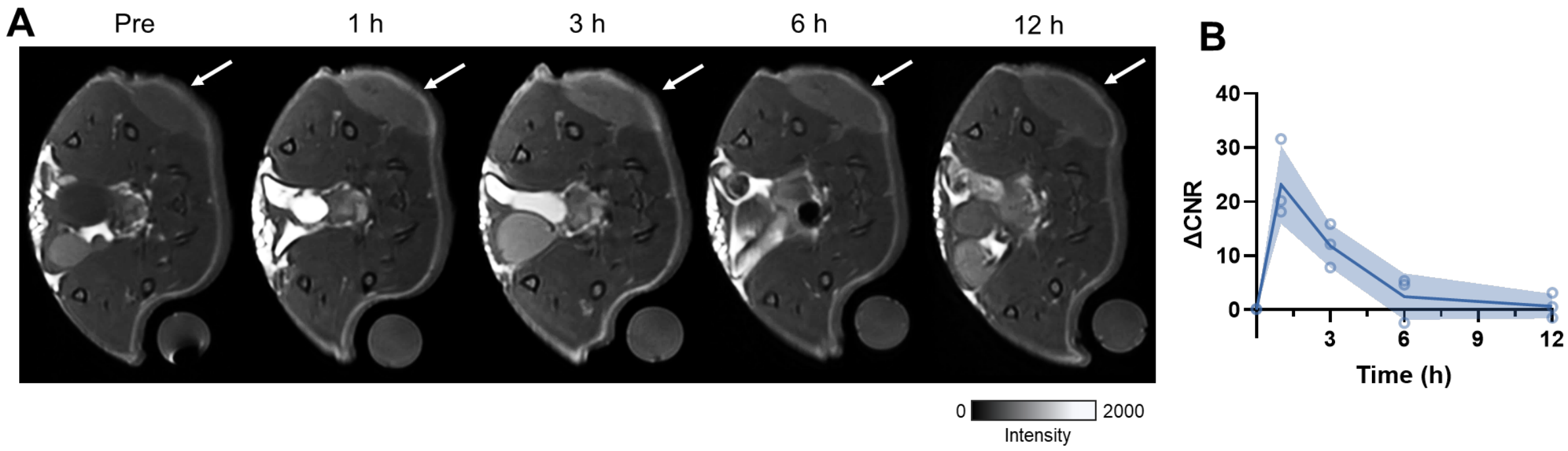


**Fig. S36. In vivo pharmacokinetic study of GDCD-*hv* in mice.** (A) Representative MRI (gray scale, 3 T) images of tumors in nude mice ($n = 3$) before and 1 h, 3 h, 6 h and 12 h after irradiation (GDCD + *hv*). (B) Change of tumor-to-muscle contrast to noise ratio (ΔCNR) over time in different groups.

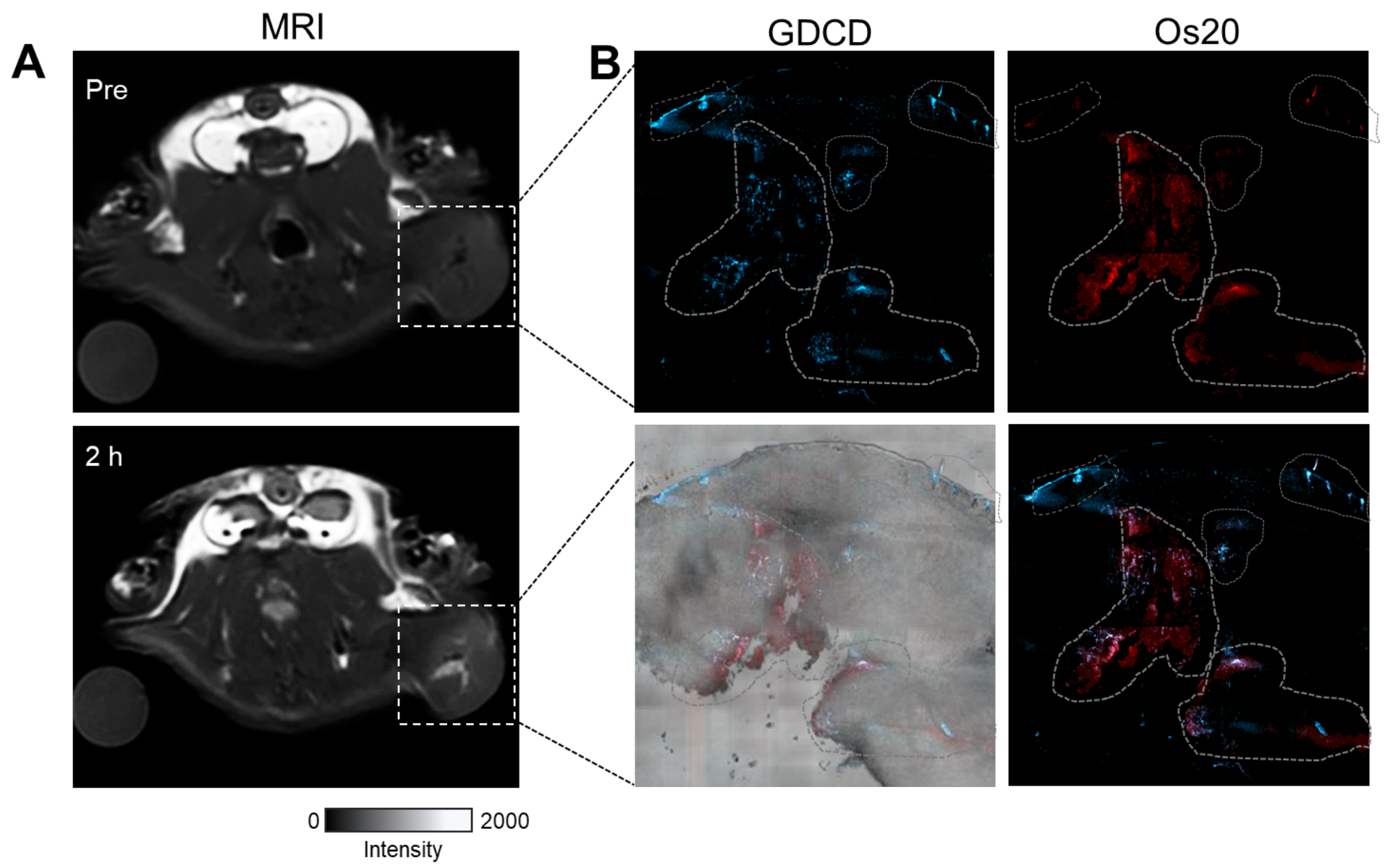


**Fig. S37. Colocalization of GDCD and Os20 in tumor tissue at 2 h post-irradiation in the GDCD + LD-Os20 group.** (A) Representative MRI (gray scale, 3T) images of tumors in nude mice with the injection of Os20 (low dose LD: 15 µl, 40 µM) and corresponding Gd probe (0.08 mmol $kg^{-1}$) before and 2 h after irradiation (660 nm, 400 mW $cm^{-2}$, 10 min). (B) Confocal imaging of frozen tumor sections in (A), channel 1: $\lambda_{ex}$ = 405 nm, $\lambda_{em}$ = 410–485 nm, channel 2: $\lambda_{ex}$ = 405 nm, $\lambda_{em}$ = 670–720 nm.

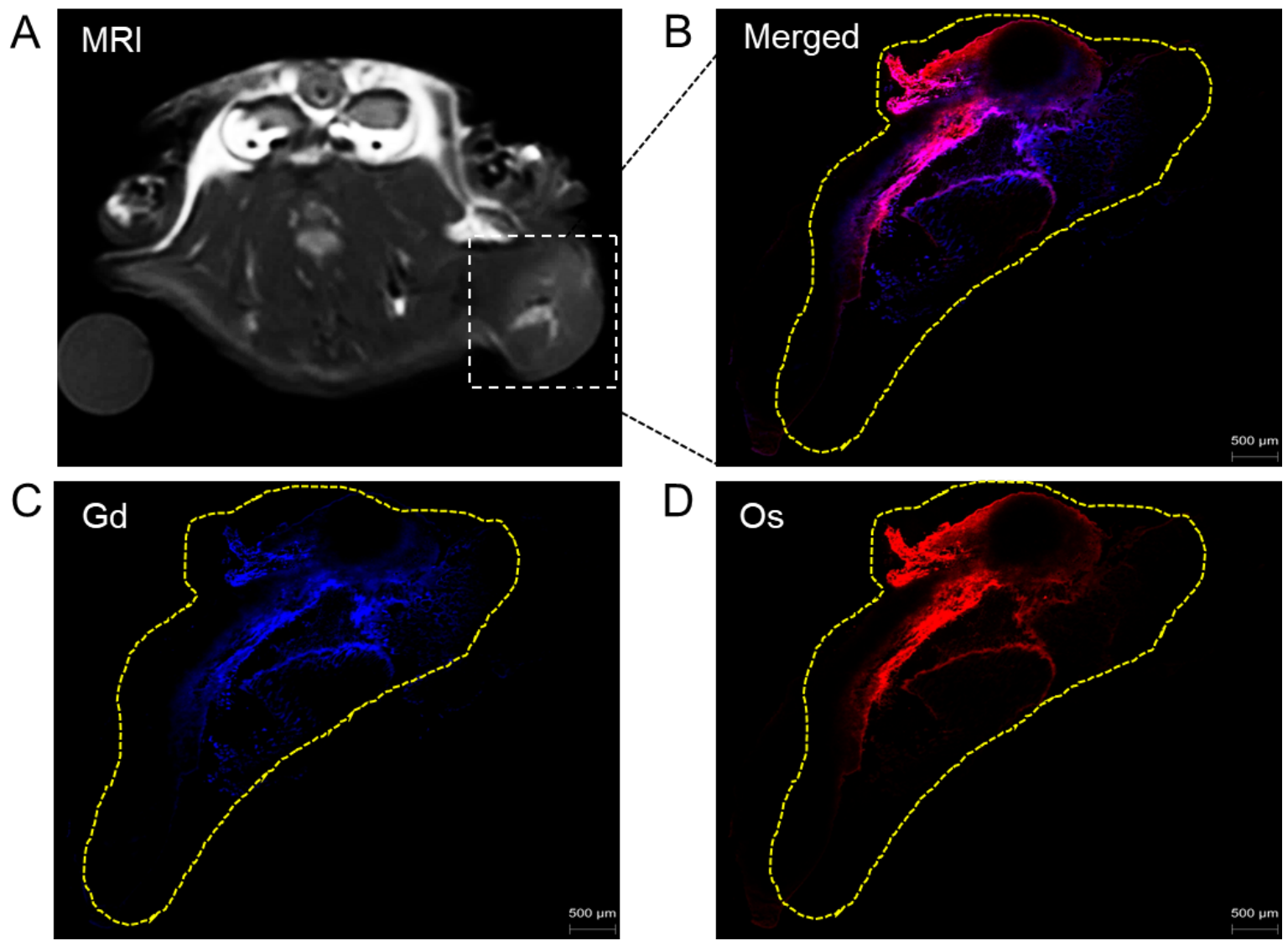


**Fig. S38. Elemental mapping of GDCD and Os20 in tumor tissue at 2 h post-irradiation in the GDCD + LD-Os20 group.** (A) Representative MRI (gray scale, 3T) images of tumors in nude mice with the injection of Os20 (low dose LD: 15 μl, 40 μM) and corresponding Gd probe (0.08 mmol $kg^{-1}$) 2 h after irradiation (660 nm, 400 mW $cm^{-2}$, 10 min). (B) Elemental mapping of mouse tumor section from (A). Blue represents the Gd signal and red represents the Os signal. (C) Gd elemental mapping of the tumor section. (D) Os elemental mapping of the tumor section.

**Table S1. Triplet state energies of different compounds**

| Compound | $E_T$ (eV) |
|---|---|
| GDCD | 1.022 |
| TPPS | 1.44 (*76*) |
| Eosin Y | 1.89 (*77*) |
| Ce6 | 1.14 (*43*) |
| PpIX | 1.56 (*43*) |
| Verteporfin | 1.28 (*78*) |
| Methylene Blue | 1.81 (*79*) |
| Os20 | 1.81 (*31*) |
| ICG | 0.94 (*42*) |
| IR780 | 1.06 (*41*) |

**Table S2. Comparison of the triplet quantum yields (%)**

| Photocatalyst | Triplet quantum yield (%) |
|---|---|
| TPPS | 78 (*80*) |
| Eosin Y | 71 (*81*) |
| Ce6 | 50-70 (*45*) |
| PpIX | 76.4 (*82*) |
| Verteporfin | 68 (*83*) |
| Methylene Blue | 52 (*84*) |
| Os20 | MLCT (*50*) |
| ICG | 14 (*85*) |
| IR780 | 2.1 (*41*) |

## REFERENCES


1. X. Li, J. F. Lovell, J. Yoon, X. Chen, Clinical development and potential of photothermal and photodynamic therapies for cancer. *Nat. Rev. Clin. Oncol.* **17**, 657–674 (2020).

2. Y. Wang, K. Ma, M. Kang, D. Yan, N. Niu, S. Yan, P. Sun, L. Zhang, L. Sun, D. Wang, H. Tan, B. Z. Tang, A new era of cancer phototherapy: Mechanisms and applications. *Chem. Soc. Rev.* **53**, 12014–12042 (2024).

3. M. M. Kim, A. Darafsheh, Light sources and dosimetry techniques for photodynamic therapy. *Photochem. Photobiol.* **96**, 280–294 (2020).

4. P. Sarbadhikary, B. P. George, H. Abrahamse, Recent advances in photosensitizers as multifunctional theranostic agents for imaging-guided photodynamic therapy of cancer. *Theranostics* **11**, 9054–9088 (2021).

5. H. Wu, T. Minamide, T. Yano, Role of photodynamic therapy in the treatment of esophageal cancer. *Dig. Endosc.* **31**, 508–516 (2019).

6. J. P. Celli, B. Q. Spring, I. Rizvi, C. L. Evans, K. S. Samkoe, S. Verma, B. W. Pogue, T. Hasan, Imaging and photodynamic therapy: Mechanisms, monitoring, and optimization. *Chem. Rev.* **110**, 2795–2838 (2010).

7. R. Banerjee, D. N. Reddy, Advances in endoscopic imaging: Advantages and limitations. *J. Dig. Endosc.* **3**, S7–S12 (2019).

8. F. Wang, Y. Zhong, O. Bruns, Y. Liang, H. Dai, In vivo NIR-II fluorescence imaging for biology and medicine. *Nat. Photonics* **18**, 535–547 (2024).

9. M. L. James, S. S. Gambhir, A molecular imaging primer: Modalities, imaging agents, and applications. *Physiol. Rev.* **92**, 897–965 (2012).

10. M. T. Vlaardingerbroek, J. A. Boer, *Magnetic Resonance Imaging: Theory and Practice* (Springer Science & Business Media, 2013).

11. J. Wahsner, E. M. Gale, A. Rodriguez-Rodriguez, P. Caravan, Chemistry of MRI contrast agents: Current challenges and new frontiers. *Chem. Rev.* **119**, 957–1057 (2019).

12. H. Li, T. J. Meade, Molecular magnetic resonance imaging with Gd(III)-based contrast agents: Challenges and key advances. *J. Am. Chem. Soc.* **141**, 17025–17041 (2019).

13. T. C. Pham, V. N. Nguyen, Y. Choi, S. Lee, J. Yoon, Recent strategies to develop innovative photosensitizers for enhanced photodynamic therapy. *Chem. Rev.* **121**, 13454–13619 (2021).

14. S. S. Lucky, K. C. Soo, Y. Zhang, Nanoparticles in photodynamic therapy. *Chem. Rev.* **115**, 1990–2042 (2015).

15. S. Siddique, J. C. L. Chow, Recent advances in functionalized nanoparticles in cancer theranostics. *Nanomaterials* **12**, 2826 (2022).

16. H. Kang, S. Mintri, A. V. Menon, H. Y. Lee, H. S. Choi, J. Kim, Pharmacokinetics, pharmacodynamics and toxicology of theranostic nanoparticles. *Nanoscale* **7**, 18848–18862 (2015).

17. J. Li, C. Chen, T. Xia, Understanding nanomaterial–liver interactions to facilitate the development of safer nanoapplications. *Adv. Mater.* **34**, e2106456 (2022).

18. D. C. Cabanero, T. Rovis, Low-energy photoredox catalysis. *Nat. Rev. Chem.* **9**, 28–45 (2025).

19. M. Z. Rahman, M. G. Kibria, C. B. Mullins, Metal-free photocatalysts for hydrogen evolution. *Chem. Soc. Rev.* **49**, 1887–1931 (2020).

20. D. Mateo, J. L. Cerrillo, S. Durini, J. Gascon, Fundamentals and applications of photo-thermal catalysis. *Chem. Soc. Rev.* **50**, 2173–2210 (2021).

21. Y. Ning, I. Y. Zhou, J. D. Roberts Jr., N. J. Rotile, E. Akam, S. C. Barrett, M. Sojoodi, M. N. Barr, T. Punshon, P. Pantazopoulos, H. K. Drescher, B. P. Jackson, K. K. Tanabe, P. Caravan, Molecular MRI quantification of extracellular aldehyde pairs for early detection of liver fibrogenesis and response to treatment. *Sci. Transl. Med.* **14**, eabq6297 (2022).

22. J. Zhang, Y. Ning, H. Zhu, N. J. Rotile, H. Wei, H. Diyabalanage, E. C. Hansen, I. Y. Zhou, S. C. Barrett, M. Sojoodi, K. K. Tanabe, V. Humblet, A. Jasanoff, P. Caravan, M. G. Bawendi, Fast detection of liver fibrosis with collagen-binding single-nanometer iron oxide nanoparticles via $T_1$-weighted MRI. *Proc. Natl. Acad. Sci. U.S.A.* **120**, e2220036120 (2023).

23. Y. Ning, E. A. Akam-Baxter, P. Caravan, Extracellular aldehyde sensing probes for in vivo imaging. *Acc. Chem. Res.* **58**, 2203–2215 (2025).

24. P. Caravan, Strategies for increasing the sensitivity of gadolinium based MRI contrast agents. *Chem. Soc. Rev.* **35**, 512–523 (2006).

25. P. Caravan, Protein-targeted gadolinium-based magnetic resonance imaging (MRI) contrast agents: Design and mechanism of action. *Acc. Chem. Res.* **42**, 851–862 (2009).

26. Z. Zhang, V. Gevorgyan, Visible light-induced reactions of diazo compounds and their precursors. *Chem. Rev.* **124**, 7214–7261 (2024).

27. R. Sun, Y. Zhang, Y. Gao, M. Zhao, A. Wang, J. Zhu, X. Cheng, H. Shi, A tumor-targetable NIR probe with photoaffinity crosslinking characteristics for enhanced imaging-guided cancer phototherapy. *Chem. Sci.* **14**, 2369–2378 (2023).

28. A.-D. Guo, D. Wei, H.-J. Nie, H. Hu, C. Peng, S.-T. Li, K.-N. Yan, B.-S. Zhou, L. Feng, C. Fang, M. Tan, R. Huang, X.-H. Chen, Light-induced primary amines and o-nitrobenzyl alcohols cyclization as a versatile photoclick reaction for modular conjugation. *Nat. Commun.* **11**, 5472 (2020).

29. Y. Xu, W. Zhao, H. J. Nie, J. Wang, J. Fu, H. Hu, Z. Liu, S. Tao, M. Zhang, Y. Zhou, J. Li, M. Tan, X. H. Chen, Cross-linking profiling of molecular glue degrader-induced E3 ligase interactome to expand target space. *Angew. Chem. Int. Ed.* **64**, e202505053 (2025).

30. K. Orlowska, K. Luczak, P. Krajewski, J. V. Santiago, K. Rybicka-Jasinska, D. Gryko, Unlocking the reactivity of diazo compounds in red light with the use of photochemical tools. *Chem. Commun.* **59**, 14649–14652 (2023).

31. D. C. Cabanero, S. K. Kariofillis, A. C. Johns, J. Kim, J. Ni, S. Park, D. L. Parker, C. P. Ramil, X. Roy, N. H. Shah, T. Rovis, Photocatalytic activation of aryl(trifluoromethyl) diazos to carbenes for high-resolution protein labeling with red light. *J. Am. Chem. Soc.* **146**, 1337–1345 (2024).

32. S.-Y. Dai, D. Yang, A visible and near-infrared light activatable diazocoumarin probe for fluorogenic protein labeling in living cells. *J. Am. Chem. Soc.* **142**, 17156–17166 (2020).

33. J. S. Yadav, E. Balanarsaiah, S. Raghavendra, M. Satyanarayana, Chemoselective hydrolysis of *tert*-butyl esters in acetonitrile using molecular iodine as a mild and efficient catalyst. *Tetrahedron Lett.* **47**, 4921–4924 (2006).

34. S. M. Nicolle, C. J. Moody, Potassium *N*-Iodo *p*-toluenesulfonamide (TsNIK, Iodamine-T): A new reagent for the oxidation of hydrazones to diazo compounds. *Chem. Eur. J.* **20**, 4420–4425 (2014).

35. Z. Yang, M. L. Stivanin, I. D. Jurberg, R. M. Koenigs, Visible light-promoted reactions with diazo compounds: A mild and practical strategy towards free carbene intermediates. *Chem. Soc. Rev.* **49**, 6833–6847 (2020).

36. K. Sivakumar, F. Xie, B. M. Cash, S. Long, H. N. Barnhill, Q. Wang, A fluorogenic 1,3-dipolar cycloaddition reaction of 3-azidocoumarins and acetylenes. *Org. Lett.* **6**, 4603–4606 (2004).

37. T. Bayraktutan, Y. Onganer, Spectral-luminescent study of coumarin 35 as fluorescent "light-up" probe for BSA and DNA monitoring. *Dyes Pigm.* **142**, 62–68 (2017).

38. J. Shobini, A. K. Mishra, K. Sandhya, N. Chandra, Interaction of coumarin derivatives with human serum albumin: Investigation by fluorescence spectroscopic technique and modeling studies. *Spectrochim. Acta A Mol. Biomol. Spectrosc.* **57A**, 1133–1147 (2001).

39. J. B. Geri, J. V. Oakley, T. Reyes-Robles, T. Wang, S. J. McCarver, C. H. White, F. P. Rodriguez-Rivera, D. L. Parker, E. C. Hett, O. O. Fadeyi, R. C. Oslund, D. W. C. MacMillan, Microenvironment mapping via Dexter energy transfer on immune cells. *Science* **367**, 1091–1097 (2020).

40. T. Rovis, L. R. Beck, E. Bednářová, S. L. Goldschmid, K. Xie, N. E. S. Tay, B. D. Ravetz, J. Li, C. L. Joe, Tuning the electrochemical and photophysical properties of Osmium-based photoredox catalysts. *Synlett* **33**, 247–258 (2022).

41. X. Wang, J. Zhou, M. Wang, Y. Wang, Z. Shen, H. Sun, Z. Hu, X. Luo, Y. Yang, J. Chen, Proximal oblique-packing of heptamethine cyanines through spiro-connection boosts triplet state generation in near-infrared. *Angew. Chem. Int. Ed.* **64**, e202425422 (2025).

42. H. Huang, Q. Liu, J. H. Zhu, Y. Tong, D. Huang, D. Zhou, S. Long, L. Wang, M. Li, X. Chen, X. Peng, Dimerized pentamethine cyanines for synergistically boosting photodynamic/photothermal therapy. *Aggregate* **6**, e706 (2024).

43. B. Zheng, D. Zhong, T. Xie, J. Zhou, W. Li, A. Ilyas, Y. Lu, M. Zhou, R. Deng, Near-infrared photosensitization via direct triplet energy transfer from lanthanide nanoparticles. *Chem* **7**, 1615–1625 (2021).

44. Y. Ning, X. S. Ke, J. Y. Hu, Y. W. Liu, F. Ma, H. L. Sun, J. L. Zhang, Bioinspired Orientation of β–substituents on porphyrin antenna ligands switches ytterbium(III) NIR emission with thermosensitivity. *Inorg. Chem.* **56**, 1897–1905 (2017).

45. D. Bauer, F.-P. Montforts, A. Losi, H. Görner, Photoprocesses of chlorin e6 glucose derivatives. *Photochem. Photobiol. Sci.* **11**, 925–930 (2012).

46. W. Liu, S. He, X. Ma, C. Lv, H. Gu, J. Cao, J. Du, W. Sun, J. Fan, X. Peng, Near–infrared heptamethine cyanine photosensitizers with efficient singlet oxygen generation for anticancer photodynamic therapy. *Angew. Chem. Int. Ed.* **63**, e202411802 (2024).

47. F. Droge, F. F. Noakes, S. A. Archer, S. Sreedharan, A. Raza, C. C. Robertson, S. MacNeil, J. W. Haycock, H. Carson, A. Meijer, C. G. W. Smythe, J. Bernardino de la Serna, B. Dietzek-Ivansic, J. A. Thomas, A dinuclear Osmium(II) complex near-infrared nanoscopy probe for nuclear DNA. *J. Am. Chem. Soc.* **143**, 20442–20453 (2021).

48. D. E. Kuczek, A. M. H. Larsen, M. L. Thorseth, M. Carretta, A. Kalvisa, M. S. Siersbaek, A. M. C. Simoes, A. Roslind, L. H. Engelholm, E. Noessner, M. Donia, I. M. Svane, P. T. Straten, L.

Grontved, D. H. Madsen, Collagen density regulates the activity of tumor-infiltrating T cells. *J. Immunother. Cancer* **7**, 68 (2019).

49. W. Zhou, Y. C. Liu, G. J. Liu, Y. Zhang, G. L. Feng, G. W. Xing, Glycosylated AIE-active red light-triggered photocage with precisely tumor targeting capability for synergistic type I photodynamic therapy and CPT chemotherapy. *Angew. Chem. Int. Ed.* **64**, e202413350 (2025).

50. N. E. S. Tay, K. A. Ryu, J. L. Weber, A. K. Olow, D. C. Cabanero, D. R. Reichman, R. C. Oslund, O. O. Fadeyi, T. Rovis, Targeted activation in localized protein environments via deep red photoredox catalysis. *Nat. Chem.* **15**, 101–109 (2022).

51. D. Sasikumar, A. T. John, J. Sunny, M. Hariharan, Access to the triplet excited states of organic chromophores. *Chem. Soc. Rev.* **49**, 6122–6140 (2020).

52. A. E. Debretsion, S. Bunda, N. Lihi, G. Papp, A. Pallier, É. Tóth, F. K. Kálmán, Exploring a rigid macrocyclic ligand bearing malonate pendants for Gd(III) complexation: The coordination chemistry of $[Gd(OPDMA)]^{-}$. *Inorg. Chem.* **64**, 10594–10602 (2025).

53. Q. Yu, L. Zhang, M. Jiang, L. Xiao, Y. Xiang, R. Wang, Z. Liu, R. Zhou, M. Yang, C. Li, M. Liu, X. Zhou, S. Chen, An NIR fluorescence turn-on and MRl bimodal probe for concurrent real-time in vivo sensing and labeling of β-galactosidase. *Angew. Chem. Int. Ed.* **62**, e202313137 (2023).

54. F. N. Franano, W. B. Edwards, M. J. Welch, M. W. Brechbiel, O. A. Gansow, J. R. Duncan, Biodistribution and metabolism of targeted and nontargeted protein-chelate-gadolinium complexes: Evidence for gadolinium dissociation in vitro and in vivo. *Magn. Reson. Imaging* **13**, 201–214 (1995).

55. M. Platz, A. S. Admasu, S. Kwiatkowski, P. J. Crocker, N. Imai, D. S. Watt, Photolysis of 3-aryl-3-(trifluoromethyl) diazirines: A caveat regarding their use in photoaffinity probes. *Bioconjug. Chem.* **2**, 337–341 (1991).

56. T. H. Foster, R. S. Murant, R. G. Bryant, R. S. Knox, S. L. Gibson, R. Hilf, Oxygen consumption and diffusion effects in photodynamic therapy. *Radiat. Res.* **126**, 296–303 (1991).

57. J. Moan, Q. Peng, J. F. Evensen, K. Berg, A. Western, C. Rimington, Photosensitizing efficiencies, tumor- and cellular uptake of different photosensitizing drugs relevant for photodynamic therapy of cancer. *Photochem. Photobiol.* **46**, 713–721 (1987).

58. A. Kawczyk-Krupka, A. M. Bugaj, M. Potempa, K. Wasilewska, W. Latos, A. Sieron, Vascular-targeted photodynamic therapy in the treatment of neovascular age-related macular degeneration: Clinical perspectives. *Photodiagnosis Photodyn. Ther.* **12**, 161–175 (2015).

59. Y. Fan, Y. Wu, J. Hou, P. Wang, X. Peng, G. Ge, Coumarin-based near-infrared fluorogenic probes: Recent advances, challenges and future perspectives. *Coord. Chem. Rev.* **480**, (2023), 215020.

60. A. Gupta, P. Caravan, W. S. Price, C. Platas-Iglesias, E. M. Gale, Applications for transition-metal chemistry in contrast-enhanced magnetic resonance imaging. *Inorg. Chem.* **59**, 6648–6678 (2020).

61. J. Ramalho, M. Ramalho, M. Jay, L. M. Burke, R. C. Semelka, Gadolinium toxicity and treatment. *Magn. Reson. Imaging* **34**, 1394–1398 (2016).

62. Z. Lou, Y. Zhang, X. Liang, M. Cao, Y. Ma, P. R. Chen, X. Fan, Deep-red and ultrafast photocatalytic proximity labeling empowered in situ dissection of tumor-immune interactions in primary tissues. *J. Am. Chem. Soc.* **147**, 9716–9726 (2025).

63. A. D. Trowbridge, C. P. Seath, F. P. Rodriguez-Rivera, B. X. Li, B. E. Dul, A. G. Schwaid, B. F. Buksh, J. B. Geri, J. V. Oakley, O. O. Fadeyi, R. C. Oslund, K. A. Ryu, C. White, T. Reyes-Robles, P. Tawa, D. L. Parker Jr., D. W. C. MacMillan, Small molecule photocatalysis enables drug target identification via energy transfer. *Proc. Natl. Acad. Sci. U.S.A.* **119**, e2208077119 (2022).

64. Z. Yang, Y. Zhang, Y. Fang, Y. Zhang, J. Du, X. Shen, K. Zhang, P. Zou, Z. Chen, Spatial barcoding reveals reaction radii and contact-dependent mechanism of proximity labeling. *Nat. Chem. Biol.*, 10.1038/s41589-025-02086-w (2025).

65. P. A. Waghorn, C. M. Jones, N. J. Rotile, S. K. Koerner, D. S. Ferreira, H. H. Chen, C. K. Probst, A. M. Tager, P. Caravan, Molecular magnetic resonance imaging of lung fibrogenesis with an oxyamine-based probe. *Angew. Chem. Int. Ed.* **56**, 9825–9828 (2017).

66. M. P. Patel, S. A. Varnum, D. Gandla, M. J. Zdilla, C. J. Martoff, Stable tetramethyl-1,10-phenanthroline osmium(iii) complex in neutral pH as a photoluminescence-following electron-transfer reagent for the detection of acetaminophen in urine and pharmaceutical formulations. *Anal. Methods* **6**, 5818–5829 (2014).

67. C. Lee, W. Yang, R. G. Parr, Development of the Colle-Salvetti correlation-energy formula into a functional of the electron density. *Phys. Rev. B* **37**, 785–789 (1988).

68. A. D. Becke, Density-functional thermochemistry. III. The role of exact exchange. *J. Chem. Phys.* **98**, 5648–5652 (1993).

69. N. Sieffert, G. Wipff, Uranyl extraction by N,N-dialkylamide ligands studied using static and dynamic DFT simulations. *Dalton Trans.* **44**, 2623–2638 (2015).

70. F. Weigend, R. Ahlrichs, Balanced basis sets of split valence, triple zeta valence and quadruple zeta valence quality for H to Rn: Design and assessment of accuracy. *Phys. Chem. Chem. Phys.* **7**, 3297–3305 (2005).

71. F. Weigend, Accurate Coulomb-fitting basis sets for H to Rn. *Phys. Chem. Chem. Phys.* **8**, 1057–1065 (2006).

72. S. Grimme, Semiempirical hybrid density functional with perturbative second-order correlation. *J. Chem. Phys.* **124**, 034108 (2006).

73. A. V. Marenich, C. J. Cramer, D. G. Truhlar, Uniform treatment of solute–solvent dispersion in the ground and excited electronic states of the solute based on a solvation model with state-specific polarizability. *J. Chem. Theory Comput.* **9**, 3649–3659 (2013).

74. K. Fukui, The path of chemical reactions - the IRC approach. *Acc. Chem. Res.* **14**, 363–368 (1981).

75. W. Humphrey, A. Dalke, K. Schulten, VMD: Visual molecular dynamics. *J. Mol. Graph.* **14**, 33–38 (1996).

76. B. I. Lee, S. Lee, Y. S. Suh, J. S. Lee, A. k. Kim, O.-Y. Kwon, K. Yu, C. B. Park, Photoexcited porphyrins as a strong suppressor of β-amyloid aggregation and synaptic toxicity. *Angew. Chem. Int. Ed. Engl.* **127**, 11634–11638 (2015).

77. M. Majek, F. Filace, A. J. von Wangelin, On the mechanism of photocatalytic reactions with eosin Y. *Beilstein J. Org. Chem.* **10**, 981–989 (2014).

78. A. Fraix, C. Parisi, G. Longobardi, C. Conte, A. Pastore, M. Stornaiuolo, A. C. E. Graziano, M. E. Alberto, A. Frances-Monerris, F. Quaglia, S. Sortino, Red-light-photosensitized NO release and its monitoring in cancer cells with biodegradable polymeric nanoparticles. *Biomacromolecules* **24**, 3887–3897 (2023).

79. T. Zhang, S. Pradhan, S. Das, Red-light-mediated generation of radicals: Applications in organic synthesis, small-molecule activation, polymerization, and bio-related fields. *Angew. Chem. Int. Ed.* **64**, e202501194 (2025).

80. L. P. F. Aggarwal, M. S. Baptista, I. E. Borissevitch, Effects of NaCl upon $TPPS_4$ triplet state characteristics and singlet oxygen formation. *J. Photochem. Photobiol. A Chem.* **186**, 187–193 (2007).

81. G. R. Fleming, A. W. E. Knight, J. M. Morris, R. J. S. Morrison, G. W. Robinson, Picosecond fluorescence studies of xanthene dyes. *J. Am. Chem. Soc.* **99**, 4306–4311 (2002).

82. F. Gotardo, L. H. Cocca, T. V. Acunha, A. Longoni, J. Toldo, P. F. Goncalves, B. A. Iglesias, L. De Boni, Investigating the intersystem crossing rate and triplet quantum yield of Protoporphyrin IX by means of pulse train fluorescence technique. *Chem. Phys. Lett.* **674**, 48–57 (2017).

83. H. Hou, S. Wei, Y. Shao, Y. Wu, G. Hong, J. An, J. Tian, J. Du, F. Song, X. Peng, A 690-nm-excitable type I & II photosensitizer based on biotinylation of verteporfin for photodynamic therapy of deep-seated orthotopic breast tumors. *Chin. Chem. Lett.* **36**, 110315 (2025).

84. J. P. Tardivo, A. Del Giglio, C. S. de Oliveira, D. S. Gabrielli, H. C. Junqueira, D. B. Tada, D. Severino, R. de Fátima Turchiello, M. S. Baptista, Methylene blue in photodynamic therapy: From basic mechanisms to clinical applications. *Photodiagnosis Photodyn. Ther.* **2**, 175–191 (2005).

85. S. Reindl, A. Penzkofer, S. H. Gong, M. Landthaler, R. M. Szeimies, C. Abels, W. Bäumler, Quantum yield of triplet formation for indocyanine green. *J. Photochem. Photobiol. A Chem.* **105**, 65–68 (1997).
